# Metabolic reprogramming in fibrosis-related diseases: underlying mechanisms and therapeutics

**DOI:** 10.1186/s43556-026-00490-9

**Published:** 2026-06-05

**Authors:** Yourong Feng, Xudong Zhang, Xin Joy Wang, Chen Chen

**Affiliations:** 1 Department of Obstetrics and Gynecology, Tongji Hospital, Tongji Medical College, Huazhong University of Science and Technology, Wuhan, 430030 China; 2 National Clinical Research Center for Obstetrical and Gynecological Diseases, Wuhan, 430030 China; 3 Division of Cardiology, Tongji Hospital, Tongji Medical College and State Key Laboratory for Diagnosis and Treatment of Severe Zoonotic Infectious Diseases, Huazhong University of Science and Technology, Wuhan, 430030 China; 4 Hubei Key Laboratory of Genetics and Molecular Mechanisms of Cardiological Disorders, Wuhan, 430030 China; 5 Faculty of Biology, Medicine and Health, University of Manchester, Manchester, UK

**Keywords:** Fibrosis, Metabolic reprogramming, PTMs, Clinical translation

## Abstract

Fibrosis, which is characterized by excessive extracellular matrix deposition and tissue stiffening, impairs organ function and particularly affects the heart, lungs, liver, and kidneys. The persistent activation of fibrosis-driving cells, such as myofibroblasts, lung epithelial cells, hepatic stellate cells and tubular epithelial cells, by various transcriptional cues and posttranslational modifications (PTMs) rewires cellular substrate metabolism, most notably glucose, amino acid and lipid flux, thereby recapitulating the Warburg effect originally described in cancer cells. Although the organ-specific mechanisms of fibrosis remain incompletely elucidated, different forms of fibrosis share common features of metabolic reprogramming. In this review, we first introduce the core metabolic pathways involved in fibrotic disorders including glycolysis, glutaminolysis and lipid metabolism. Next, we focus on organ-specific metabolic alterations and their regulatory mechanisms in fibrosis-related diseases, including those of the heart, lungs, liver, kidneys, skin, peritoneum and glands, and discuss their underlying mechanisms: altered enzyme expression, subcellular localization and PTMs, within each organ-specific context. In addition to their canonical catalytic functions, enzymes that participate in glucose and lipid metabolism influence fibrosis through nonenzymatic activities mediated by PTMs, including phosphorylation, acetylation, ubiquitination, and the newly recognized lactylation. We further summarize mechanistic insights across organs and metabolic crosstalk in fibrosis. Finally, we discuss the current clinical translation and applications of these pathways. This review provides a reference for further research on metabolic reprogramming, highlights the role of aberrant metabolism and its underlying mechanisms in fibrosis across organs, and identifies emerging therapeutic strategies targeting glycolysis and lipid metabolism.

## Introduction

Fibrosis is a chronic and progressive pathological response to various tissue insults or stresses, with related diseases affecting mainly the heart, lungs, liver, and kidneys and contributing to nearly 45% of deaths in industrialized nations [1]. It is recognized as one of the most important global health issues, with an annual incidence of approximately 1 in 20 [2]. A hallmark of fibrosis is the imbalance between the excessive accumulation and degradation of the extracellular matrix (ECM). Fibroblasts (Fbs), along with other mesenchymal cells, such as lung epithelial cells and hepatic stellate cells, are the major cell types responsible for maintaining ECM homeostasis [3]. Given the inherent heterogeneity in tissue structure and microenvironmental signalling pathways across organs, fibrogenesis occurs through complicated, context-dependent pathways, yielding an organ-specific phenotype. Despite numerous comprehensive advancements in understanding fibrosis, the underlying mechanisms remain incompletely elucidated, thereby retarding the development of precision antifibrotic therapies.

The activation and proliferation of these mesenchymal cells are regulated by various cytokines, such as transforming growth factor-β (TGF-β), interleukin-1 (IL-1), and tumor necrosis factor (TNF). TGF-β, which is recognized as the most potent fibrotic cytokine, is initially synthesized as a precursor that forms a latent complex with the latency-associated peptide (LAP) and latent TGF-β-binding protein (LTBP) before it is secreted into the ECM. Upon activation, TGF-β binds to its receptor, initiating both canonical and noncanonical signalling pathways. The canonical pathway involves the phosphorylation of the SMAD family member 2 (SMAD2) and SMAD3 and their nuclear translocation to induce profibrotic gene transcription. Noncanonical pathways, including the mitogen-activated protein kinase (MAPK), PI3K (phosphatidylinositol 3-kinase), and JUN amino-terminal kinase (JNK) pathways, also contribute to ECM deposition [4]. Recent evidence has established that in addition to the canonical TGF-β cascade, cellular energy metabolism—particularly glucose and lipid flux—is actively involved in the progression of various fibrosis-related diseases. Importantly, these metabolic alterations can promote fibrogenesis independently of TGF-β signalling under certain pathological conditions [5, 6]. Emerging evidence has indicated that post-translational modifications (PTMs) are a key mechanism through which metabolic reprogramming occurs. Modifications such as phosphorylation, acetylation, and ubiquitination dynamically regulate the activity, stability, and localization of metabolic enzymes, directly fine-tuning pathways such as glycolysis. Notably, metabolites such as lactate from the glycolysis pathway can induce PTMs through lactylation, which in turn increases the expression of profibrotic genes. PTMs are typically reversible and occur in various cellular compartments; thus, they serve as critical molecular switches that translate metabolic alterations into sustained fibrotic responses [7–9].

With this understanding of how metabolic reprogramming emerges in fibrosis, we now turn to a detailed examination of the specific pathways involved. We first elaborate on the key metabolic processes, including glycolysis, glutaminolysis and lipid metabolism, during fibrogenesis. We then focus on the aberrant transcriptional regulation and PTMs of metabolic enzymes involved in these pathways during fibrosis in the heart, lungs, liver, kidneys, skin, peritoneum and glands. Beyond organ-specific alterations, we further discuss common fibrotic mechanisms shared across organs, the crosstalk between metabolism and the immune system, and how mitochondrial dysfunction serves as a central hub driving metabolic reprogramming and fibrosis progression. Finally, we review current clinical trials targeting these metabolic pathways, as well as recent advances in metabolites for fibrosis diagnosis and therapy.

## Core metabolic pathways involved in fibrotic disorders

In response to tissue injury, mesenchymal cells, including Fbs, undergo significant metabolic adaptations, such as increased aerobic glycolysis, to meet the high energy demands of fibrogenic activation. In turn, increased levels of glycolytic enzymes and byproducts, such as lactate, act as key drivers contributing to the progression of fibrosis [10, 11]. In addition to glycolysis, mesenchymal cells also rewire amino acid metabolism by reprogramming glutaminolysis and lipid metabolism through the orchestration of fatty acid oxidation (FAO) and synthesis [5, 6, 12]. Then, we introduce the fundamental processes of the three metabolic pathways (including glycolysis, glutaminolysis and lipid metabolism) that are closely connected through key intermediates.

### The basic process of glycolysis

Glycolysis is a metabolic process that utilizes one glucose molecule to form two molecules of pyruvate and release two adenosine triphosphate (ATP) molecules. In addition to providing energy, glycolytic intermediates can serve as building blocks that are funnelled into multiple biosynthetic pathways, such as the pentose phosphate and hexosamine pathways, thereby facilitating ECM deposition.

**Fig. 1:**
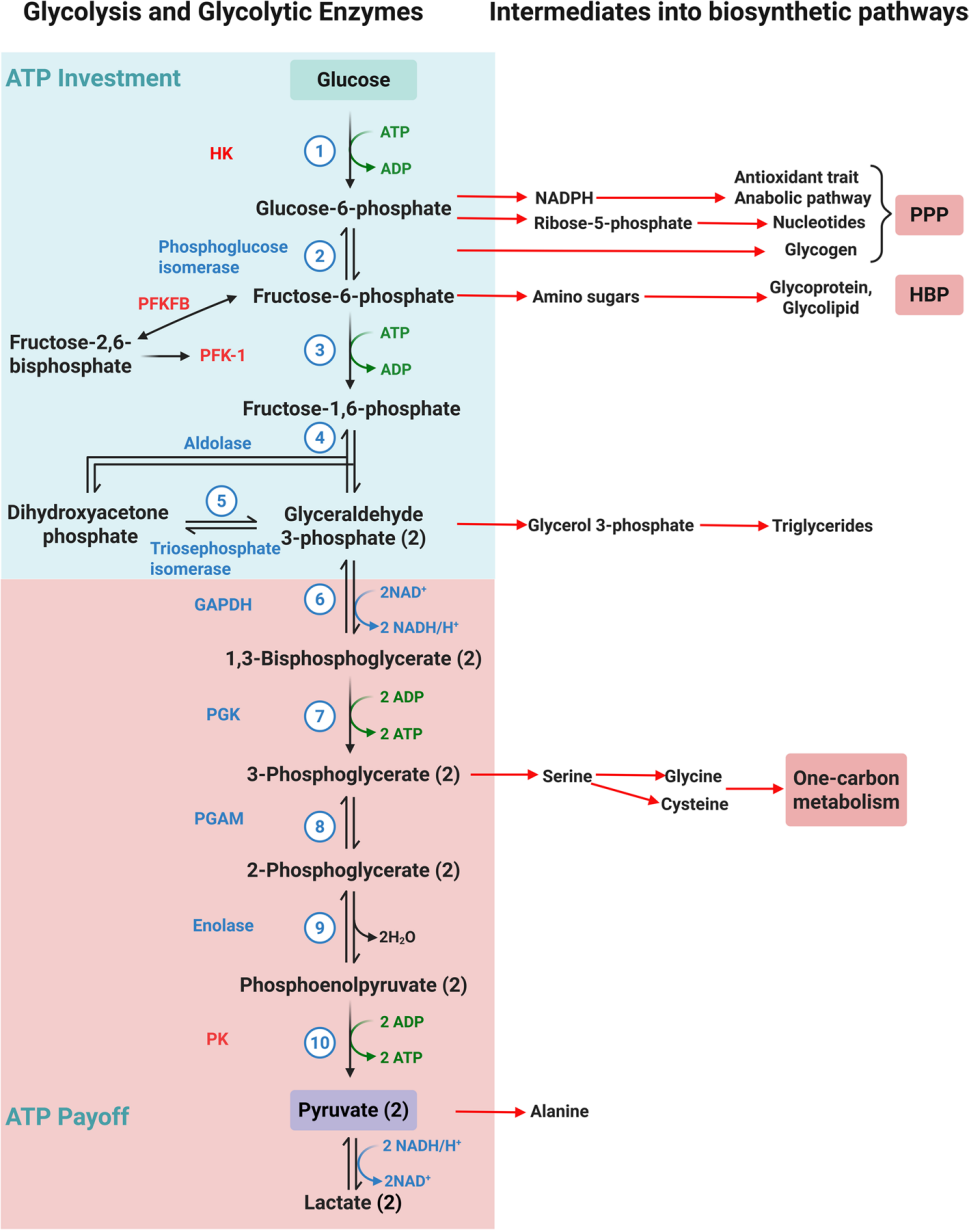
Overview of the basic process in glycolysis and intermediates into biosynthetic pathways. One molecule of glucose undergoes two phases to produce two molecules of ATP, two molecules of NADH, and two molecules of pyruvate. The intermediates of glycolysis can be precursors of multiple biosynthetic pathways. The critical enzymes of glycolysis are marked with red font. HK, hexokinase; PFK-1, phosphofructokinase; PFKFB, 6-phosphofructo-2-kinase/fructose-2,6-biphosphatase; GAPDH, glyceraldehyde 3-phosphate dehydrogenase; PGK, phosphoglycerate kinase; PGAM, phosphoglycerate mutase; PK, pyruvate kinase; PPP, pentose phosphate pathway; HBP, hexosamine biosynthetic pathway (Figure was created with Biorender.com)

Glycolysis, a cytosolic metabolic pathway, can be divided into two core phases, namely the preparatory phase (ATP investment) and the payoff phase (ATP generation), alongside complementary biosynthetic pathways that branch from its key intermediates [13] (Fig. 1). During the ATP investment phase, one glucose molecule is converted into two glyceraldehyde 3-phosphate (G3P) molecules through the sequential actions of hexokinase (HK), phosphoglucose isomerase, phosphofructokinase (PFK), aldolase and triosephosphate isomerase, consuming two ATP molecules. In the ATP payoff phase, these G3P molecules are further catalyzed by a series of enzymes, including G3P dehydrogenase (GAPDH), phosphoglycerate (PG) kinase (PGK), PG mutase (PGAM), enolase (ENO), and pyruvate kinase (PK), ultimately yielding two pyruvate molecules and four ATP molecules. Overall, the glycolytic breakdown of one glucose molecule through a collection of ten reactions results in a net yield of two pyruvate molecules, two ATP molecules and two nicotinamide adenine dinucleotide (NADH) molecules. Under aerobic conditions, pyruvate is preferentially imported into mitochondria and oxidatively decarboxylated to acetyl-coenzyme A (acetyl-CoA); in contrast, oxygen limitation drives its cytoplasmic reduction to lactate. Glycolysis ends with the conversion of pyruvate to lactate, catalyzed by lactate dehydrogenase A (LDHA), a reaction that regenerates NAD⁺ from NADH. HK, PFK and PK are the three rate-limiting enzymes of the glycolytic pathway [14].

In addition to generating ATP, the intermediates of these steps could also be fed into diverse biosynthetic pathways. For instance, glucose-6-phosphate (G6P) can enter the pentose phosphate pathway (PPP) to generate nicotinamide adenine dinucleotide phosphate (NADPH), which functions as an antioxidant and contributes to anabolic processes. It also contributes to glycogen synthesis by producing ribose-5-phosphate and supports nucleotide synthesis. Fructose 6-phosphate can be diverted into the hexosamine biosynthetic pathway (HBP) to produce amino sugars, which are essential for the synthesis of glycoproteins, glycolipids, and proteoglycans. Additionally, fructose 1,6-phosphate can be metabolized to dihydroxyacetone phosphate and glycerol 3-phosphate, which serve as precursors for lipid synthesis. 3-Phosphoglycerate can be used to synthesize serine (Ser), which can subsequently be utilized to produce cysteine and glycine. In addition, pyruvate can be utilized to produce the amino acid alanine [15].

However, this glycolytic switch does not operate in isolation; rather, it is intimately interconnected with other major metabolic pathways through complex crosstalk [16–18]. Notably, the heightened glycolytic flux provides not only rapid ATP generation but also key carbon intermediates that coordinate with fatty acid metabolism and glutamine metabolism. For instance, glycolytically derived pyruvate and acetyl CoA can be diverted toward de novo lipogenesis, supplying lipids for membrane synthesis and signaling, while the metabolism of glutamine via glutaminolysis generates proline, an essential amino acid for excessive collagen deposition in fibrosis. These interconnected metabolic adaptations, namely glycolysis, fatty acid metabolism and glutaminolysis, form a coordinated network that sustains the bioenergetic, biosynthetic and redox demands of activated myofibroblasts. Therefore, understanding this metabolic crosstalk is crucial for identifying novel antifibrotic therapeutic targets. In the following sections, we will elaborate on the specific roles of fatty acid metabolism and glutamine metabolism in driving fibrogenesis.

### Glutamine metabolism, the Krebs cycle and lipid metabolism

In a nutrient-poor microenvironment, in addition to glucose, glutamine serves as the principal metabolic fuel of vigorously proliferating cells, including cancer cells and myofibroblasts [19]. Glutamine is imported into cells through specific sodium-coupled neutral amino acid transporters such as SLC38A1 and SLC1A5/ASCT2. As the most abundant amino acid, glutamine serves not only as an energy source but also as a precursor for the production of building blocks for proteins, nucleotides and lipids [20]. Within mitochondria, it is first deamidated to glutamate by glutaminase 1 and 2 (GLS1/2), and the resulting glutamate is then oxidized to α-ketoglutarate (α-KG) by glutamate dehydrogenase (GDH) or aminotransferases. Mitochondrial glutamate can also be exported to the cytosol, where it acts as a metabolic intermediate for the synthesis of glutathione and nonessential amino acids. This two-step conversion of glutamine to glutamate and then to α-KG, termed glutaminolysis, supplies carbons to the tricarboxylic acid cycle (TCA or Krebs cycle) [20].

Functioning as the cell’s metabolic hub, the TCA cycle is initiated when acetyl-CoA—a product derived from pyruvate, FAs, or amino acids—condenses with oxaloacetate (OAA), to yield the six-carbon citrate. The cycle then proceeds through a series of eight enzymatic reactions. The second step involves the isomerization of citrate to isocitrate. This step is followed by two oxidative decarboxylation reactions: isocitrate is first converted to α-KG by isocitrate dehydrogenase (IDH1/2). The produced α-KG is transformed into succinyl-CoA, releasing two carbon dioxide molecules (CO_2_) and reducing two NAD^+^ molecules to NADH. The energy from the thioester bond in succinyl-CoA is then used to produce GTP (or ATP) as it is converted to succinate. Next, succinate is oxidized to fumarate by succinate dehydrogenase (SDH), transferring hydrogen atoms to flavin adenine dinucleotide (FAD) and generating FADH_2_. Notably, SDH also functions as Complex II and is embedded in the electron transport chain. Finally, fumarate is converted to malate, which is oxidized to regenerate OAA, thus completing the cycle [21–23].

Beyond glycolysis-derived pyruvate, another primary source of acetyl-CoA is the mitochondrial β-oxidation of FA. The cellular uptake of FAs is facilitated by specific transport proteins, such as CD36. Intracellular FAs are immediately activated in the cytosol to form acyl-CoA esters via fatty acyl-CoA synthetases (ACSs), representing the initial step of oxidation. Since the mitochondrial membrane is impermeable to CoA-bound substrates, a specialized carnitine shuttle system is required for import. This process is initiated by carnitine palmitoyl transferase I (CPT1) on the outer mitochondrial membrane, which converts acyl-CoA to acylcarnitine. Acylcarnitine is then shuttled across the inner membrane by the carnitine/acylcarnitine translocase (CACT) and subsequently reconverted to acyl-CoA in the matrix by carnitine palmitoyl transferase II (CPT2). Within the mitochondrial matrix, the acyl-CoA molecule undergoes a β-oxidation spiral, including four enzymatic reactions, ultimately yielding one molecule each of acetyl-CoA, NADH, and FADH_2_ per cycle. The cycle continues repetitively until the entire FA is fully degraded into multiple acetyl-CoA units [24, 25]. In addition, acetyl-CoA serves as the fundamental material for FA synthesis. Under conditions of an energy surplus, mitochondrial citrate is exported to the cytosol. Then, ATP-citrate lyase (ACLY) cleaves citrate to regenerate acetyl-CoA. This cytosolic acetyl-CoA is subsequently carboxylated by acetyl-CoA carboxylase (ACC) to form malonyl-CoA, a crucial building block. Finally, the fatty acid synthase (FASN) complex utilizes acetyl-CoA as a primer and malonyl-CoA as a two-carbon donor in a series of sequential condensation reactions to synthesize the saturated 16-carbon fatty acid palmitate [26, 27].

**Fig. 2:**
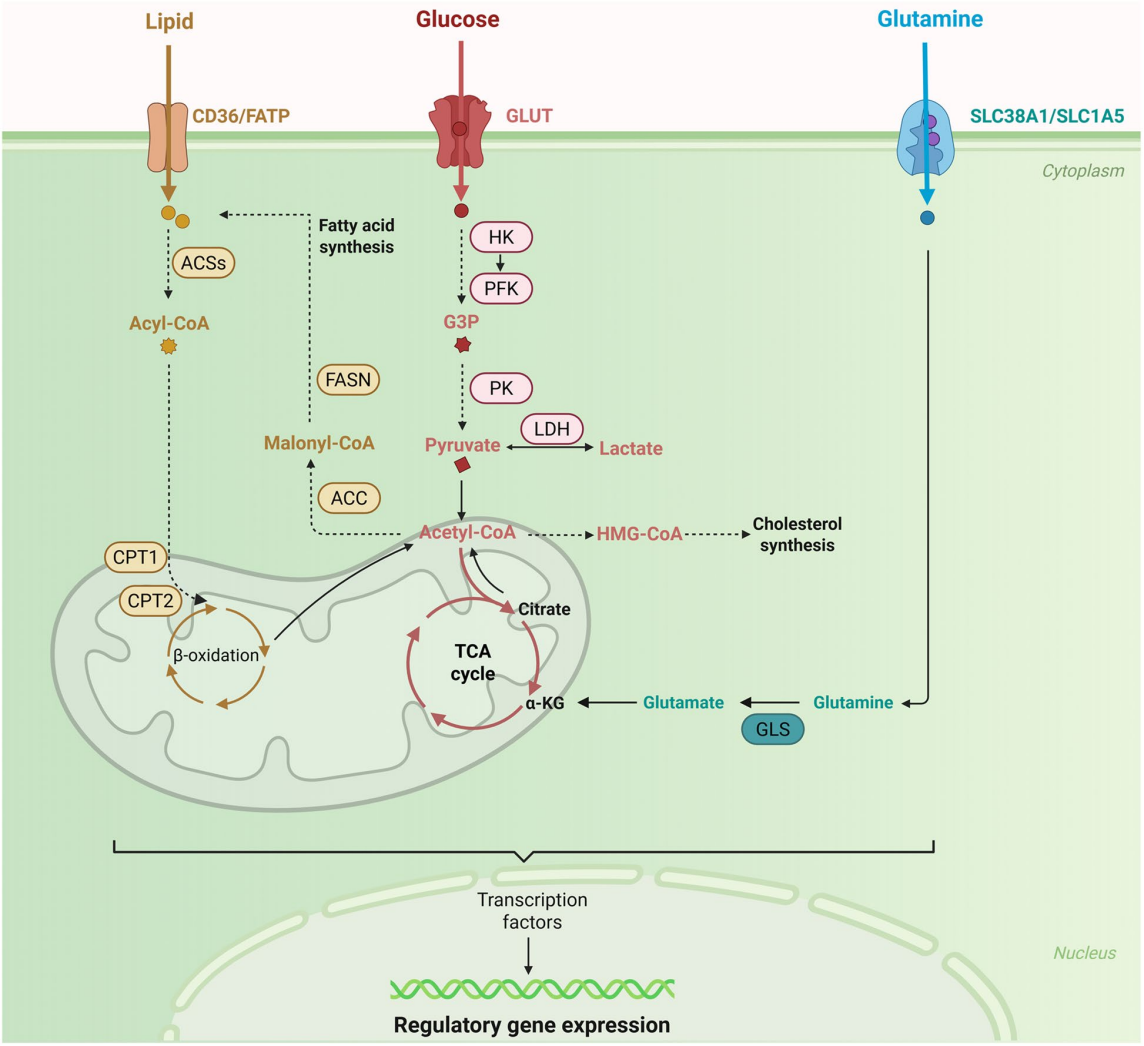
The crosstalk of lipid, glucose and glutamine metabolism, bridged by their key metabolic intermediates. Lipid metabolism fuels the TCA cycle by generating acetyl-CoA via beta-oxidation. Similarly, glycolysis contributes pyruvate for its conversion to acetyl-CoA. Glutamine metabolism replenishes depleted cycle intermediates by providing α-KG. This metabolic integration ensures a continuous supply of cellular energy and biosynthetic blocks. FATP, fatty acid transport protein; ACSs, Acyl-CoA synthetases; CPT1/2, carnitine palmitoyltransferase 1/2; FASN, fatty acid synthase; ACC, Acetyl-CoA carboxylase; GLS, glutaminase; α-KG, α-ketoglutarate (Figure was created with Biorender.com)

Metabolic reprogramming has been increasingly identified as a key characteristic of various fibrotic disorders. This reprogramming, which includes enhanced glycolysis, glutaminolysis, and bifurcated FAO, critically contributes to the activation of profibrotic pathways and the overproduction of ECM [6, 11]. A schematic overview of the interplay between the three metabolic pathways is shown in Fig. 2.

## Organ-specific metabolic alterations and their regulatory mechanisms in fibrosis-related diseases

Following the outline of core metabolic pathways, we discuss how these pathways are dysregulated in an organ-specific manner during fibrosis. While shared features such as increased glycolysis are observed across multiple organs, metabolic alterations also vary considerably between different organs and cell types. Notably, FAO exhibits considerable heterogeneity across different fibrotic conditions, with impaired FAO being a predominant finding in most fibrotic contexts, while opposite changes have been reported, such as in certain stages of liver fibrosis. Furthermore, accumulating evidence indicates that fibrotic processes in different organs are biologically interconnected, often amplifying one another through shared mechanisms such as circulating factors and inflammatory pathways. The following sections detail the distinct metabolic profiles in cardiac, pulmonary, hepatic, renal fibrosis, skin, peritoneum and glands while highlighting the systemic nature of fibrotic progression. We then summarize shared mechanistic features across different organs, discuss the interplay between metabolism and immune responses, and highlight how mitochondrial dysfunction fuels metabolic reprogramming in fibrosis.

### Cardiac fibrosis

Cardiac fibrosis is a common pathophysiological feature of most cardiovascular diseases and is primarily associated with conditions such as ischemic heart diseases (IHDs), non-ischemic cardiomyopathy (NICM), and heart failure (HF) [28]. Two forms of cardiac fibrosis have been identified: 1) reparative fibrosis, also referred to as replacement fibrosis, which functions to replace dead cardiomyocytes (CMs) with ECM and myofibroblasts (myoFbs) to preserve tissue integrity and typically occurs in IHDs, including myocardial infarction (MI) and ischemia/reperfusion (I/R) injury, and 2) reactive fibrosis, characterized by the enlargement of interstitial and perivascular areas without substantial CM depletion, which is usually observed in NICM [28].

**Fig. 3:**
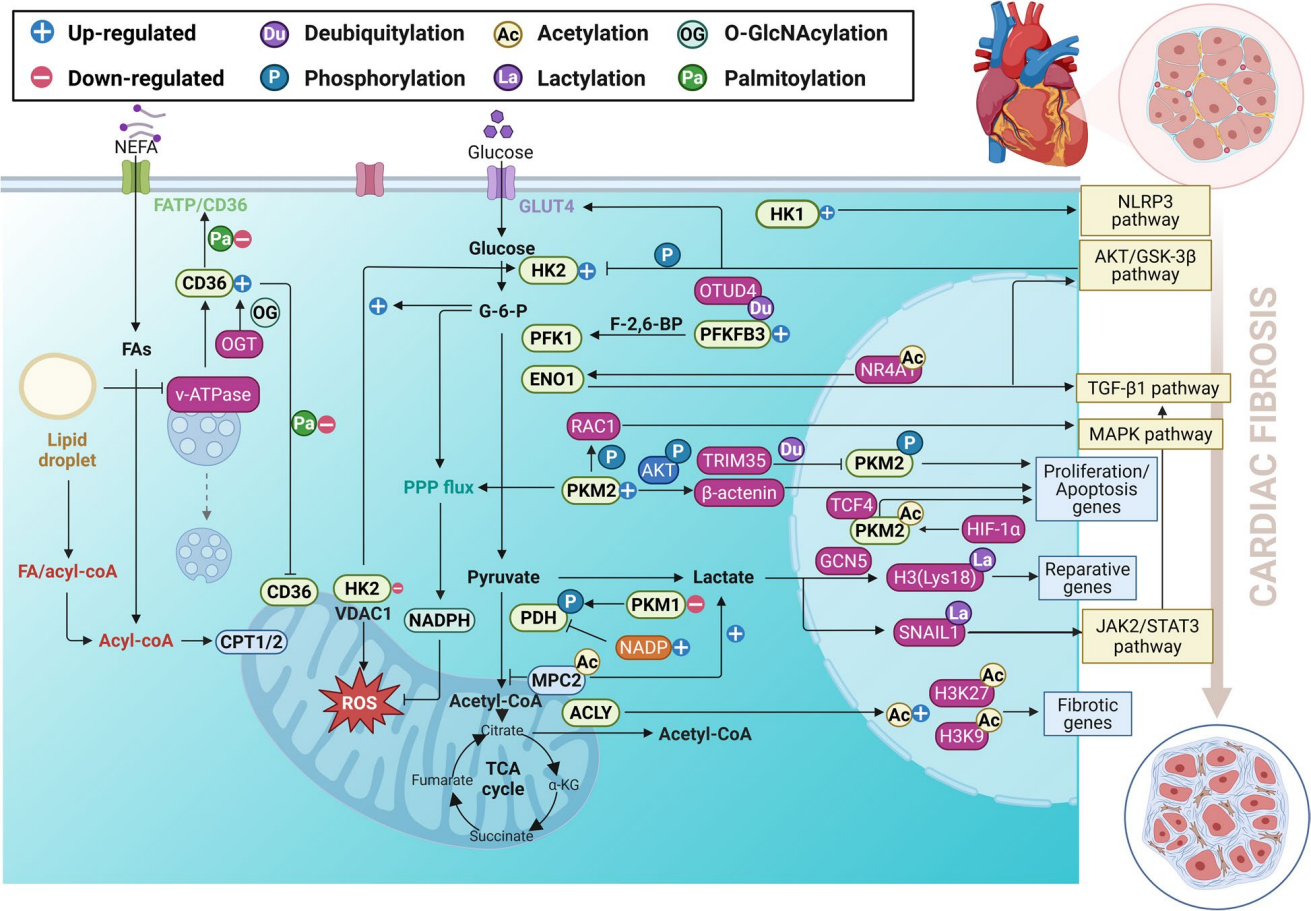
Regulation of enzymes orchestrates metabolic reprogramming in cardiac fibrosis: roles of transcription and PTMs. During cardiac fibrosis progression, glycolytic reprogramming is driven by HK2 dissociation from mitochondria, PKM2 nuclear translocation and PFKFB3 ubiquitination, along with transcriptional upregulation of HK, PKM, and PFK isoforms. CD36 PTMs and subcellular redistribution regulate lipid metabolism, while lactate contributes to fibrosis through extracellular signaling and histone lactylation. G6P, glucose 6-phosphate; VDAC1, voltage-dependent anion channel 1; GLUT4, glucose transporter 4; AKT, protein kinase B; GSK-3β, glycogen synthase kinase-3β; HIF-1α, hypoxia-inducible factor 1α; PFKFB3, phosphofructokinase-2/fructose-2,6-bisphosphatase 3; F-2,6-BP, fructose-2,6-bisphosphate; OTUD4, OTU deubiquitinase 4; LDHA/LDHB, lactate dehydrogenase A/B; MPC, mitochondrial pyruvate carrier; PDH, pyruvate dehydrogenase; TCA, tricarboxylic acid cycle; H3, histone H3; NLRP3, nucleotide-binding oligomerization domain-like receptor protein 3; TGF-β, transforming growth factor-β; RAC1, rho family, small GTP binding protein 1; MAPK, mitogen-activated protein kinase; α-MHC, α-myosin heavy chain; EndMT, endothelial-to-mesenchymal transition; OGT, O-GlcNAc transferase; ACLY, ATP-citrate lyase; NR4A1, nuclear receptor subfamily 4 group A member 1; NEFA, non‑esterified fatty acid; P, phosphorylation; Ac, acetylation; La, lactylation; Thr, threonine; Ser, serine; Lys, lysine (Figure was created with Biorender.com)

In the failing heart, a critical metabolic alteration is the shift of glucose metabolism away from efficient mitochondrial oxidation towards less efficient aerobic glycolysis. This glycolytic switch reduces ATP production and exacerbates cellular acidosis. Concurrently, neurohormonal activation of the sympathetic nervous system and the renin–angiotensin–aldosterone system (RAAS) promotes a paradoxical overreliance on FAO, significantly increasing its energy contribution. This shift is coupled with a corresponding suppression of mitochondrial glucose oxidation. When FA uptake increases, FA metabolism becomes less efficient. These coordinated dysfunctions, the glycolytic shift and impaired FAO, are not merely consequences of cardiac injury but also active drivers of pathology. They directly promote maladaptive cardiac remodelling and fibrosis, creating a vicious cycle [29, 30]. Next, we delineate the dysregulation of glycolysis and fatty acid metabolism in cardiac fibrosis, with a focus on the transcriptional control and PTMs of the key enzymes involved (Fig. 3).

#### Glycolysis in cardiac fibrosis

HK2, the predominant isoform expressed in the heart, resides either in the cytoplasm or at the outer mitochondrial membrane. Its expression and cellular localization change significantly during and after ischemia [31]. During I/R injury or prolonged cardiac ischemia, mitochondrial HK2 levels are significantly decreased, yet an increase in its levels is instrumental for stabilizing the mitochondrial membrane potential and reducing reactive oxygen species (ROS) levels through the coupling of glycolysis and glucose oxidation [31]. In acute ischemia, glycolysis increases, which might be the only source of ATP. Additionally, aerobic glycolysis results in a significant increase in G6P levels, which stimulates the dissociation of HK2 from the voltage-dependent anion channel 1 (VDAC1) and thereby triggers the permeabilization of the outer mitochondrial membrane and the release of cytochrome c, leading to the generation of ROS and an increased infarct size [32]. Alternatively, ischemic preconditioning has been shown to increase myocardial resilience through the phosphorylation of HK2 at threonine 473 (Thr473) via the protein kinase B (AKT)/glycogen synthase kinase-3β (GSK-3β) pathway. This process leads to the increased translocation of GLUT4 to the cell membrane and increased binding of HK2 to mitochondria, thus increasing resistance to ROS and reducing cell death postreperfusion [33]. In NICM, the effect of HK on fibrosis is predominantly contingent upon the specific isoform expressed. Inhibiting HK1 can alleviate isoprenaline-induced cardiac fibrosis through the suppression of NLR family pyrin domain containing 3 (NLRP3) signaling pathway [34]. Overexpression of HK2 can increase PPP bypass to mitigate ROS production and improve cardiac function, whereas the knockdown of HK2 increases mitochondrial permeability and exacerbates fibrosis induced by pressure overload [35, 36].

As a tetrameric enzyme, the expression of PFK-1 is intracellularly regulated by various substances, with fructose-2,6-bisphosphate (F-2,6-BP) being the most potent, while its level is controlled by fructose-2,6-bisphosphatase (PFKFB). In cardiac Fbs exposed to TGF-β1 and those from post-MI mice, the expression of PFKFB3 is notably increased [37]. Mechanistically, OTU deubiquitinase 4 (OTUD4) interacts with PFKFB3, impeding its ubiquitylation and degradation to maintain a highly glycolytic phenotype. Thus, targeting this interaction could be a strategy to reduce post-MI cardiac fibrosis [37]. Moreover, in NICM, the manipulation of cardiac myosin binding protein C3 (MYBPC3) in cardiac Fbs has been reported to stimulate the nuclear factor κB (NF-κB) signaling pathway and upregulate glycolysis-related genes, including PFK and LDHA. This upregulation subsequently enhances aerobic glycolysis in myocyte-derived cardiac fibroblasts (myoCFs), thus leading to hypertrophic cardiomyopathy [38]. Elevated levels of PFK-1 also reprogram cardiac glycolysis, thereby mitigating the interstitial fibrosis and diastolic dysfunction caused by transverse aortic constriction (TAC) or Angiotensin II (Ang II) [39, 40]. However, chronic and persistent activation of PFK-1 has been shown to drive fibrosis and cardiac remodeling by disrupting the balance between glycolysis and FAO [41].

PK, a rate-limiting enzyme, has four isoforms: the L-type (PKL), expressed in gluconeogenic tissues such as the liver; the R-type (PKR), expressed in erythrocytes and hematopoietic tissues; the M1-type (PKM1), expressed in cardiac and skeletal muscle as a tetramer; and the M2-type (PKM2), expressed in the lungs, spleen, kidneys, and testes. In addition to its glycolytic function, PKM2 can serve as a transcriptional regulator or protein kinase, regulating pathways such as apoptosis and proliferation upon nuclear translocation [42]. Following ischemic injury, the prolonged expression of hypoxia-inducible factor 1α (HIF-1α) promotes the expression of PKM2 over the typically predominant isoform PKM1, and the downstream effects appear to be controlled by the localization of PKM2. In the cytoplasm, PKM2 directly interacts with β-catenin, leading to its degradation by impeding AKT phosphorylation at Ser552 and Thr333 and suppressing the expression of proliferation-associated genes [43]. In contrast, nuclear-acetylated PKM2 binds to β-catenin/T-cell factor 4 (TCF4) complex, inducing the expression of genes that promote the cell cycle and inhibit apoptosis. Additionally, increased levels of PKM2 increase the activity of glucose-6-phosphate dehydrogenase (G6PDH), a rate-limiting enzyme of the PPP, thus accelerating glucose flux into the PPP anabolic pathway [44, 45]. In NICM, PKM1 is essential for maintaining the phosphorylation of Ser232 in pyruvate dehydrogenase (PDH), thereby mitigating TAC-induced cardiac remodeling and fibrosis [46]. In contrast to the cardioprotective role of PKM1, the role of PKM2 during pathological cardiac remodelling is seemingly controversial. Suppressing PKM2 can markedly alleviate the Ang II-induced cardiac Fb activation through the inhibition of the TGF-β/SMAD and Janus kinase 2 (JAK2)/signal transducer and activator of transcription 3 (STAT3) pathways. However, by phosphorylating RAC1 (rho family, small GTP binding protein) and suppressing the MAPK pathway [47], PKM2 acts as a protein kinase rather than a pyruvate kinase to alleviate cardiac function and fibrosis during the progression of TAC-induced HF [48]. Intriguingly, PKM2 plays opposing roles and is distinctly expressed in cardiac fibroblasts (CFs) and CMs during pressure overload-induced HF. In CFs, PKM2 is predominantly present in the cytoplasm, where elevated levels of this protein induce CF proliferation. Conversely, in CMs, PKM2 is phosphorylated at Ser37, leading to its preferential nuclear localization, and a decrease in the PKM2 level in CMs causes apoptosis through ubiquitination by the tripartite motif-containing protein TRIM35 [49].

In addition to key enzymes, other glycolytic enzymes also undergo PTMs, thereby participating in the regulation of cardiac fibrosis. ENO exists as a dimeric enzyme comprising three isoenzymes, ubiquitous α-enolase (ENO1), neural tissue-enriched γ-enolase (ENO2), and muscle-specific β-enolase (ENO3), each of which forms homodimers. Among these isoforms, ENO1 has been the most extensively investigated in terms of its role in glycolysis, cell growth regulation and fibrosis [46, 50]. During MI, the acetylation of nuclear receptor subfamily 4 group A member 1 (NR4A1) activates the transcription of ENO1, promoting glycolytic overload and fibrosis [51]. In addition, ENO1 contributes to fibrosis through both TGF-β1-dependent and -independent pathways [46]. In addition, ENO1 binds to cardiac troponin I autoantibody (cTnIAAb), an autoantibody whose levels are increased during acute MI (AMI), and myocardial collagen deposition and systolic dysfunction are promoted by ENO1–AKT signaling pathway [52].

LDH, a tetrameric enzyme that catalyzes the reversible redox reaction between pyruvate and L-lactate, is composed mainly of two subunits, LDHA and LDHB, which assemble into five distinct isoenzymes (LDH1–5) as homo or heterotetramers. A unique isoform, LDHC, forms LDH6 and is specific to the testes and spermatozoa [53]. LDHA is predominant in the skeletal muscle and liver, whereas LDHB is prevalent in the myocardium and is dependent upon aerobic metabolic pathways. LDHA is responsible for the conversion of pyruvate to lactate, whereas LDHB catalyzes the reverse reaction, converting lactate back to pyruvate [54]. The irreversible elimination of lactate is facilitated by PDH. During cell proliferation, PDH activity can be inhibited by the phosphorylation of its E1α subunit and the accumulation of NADH, which shifts cells towards aerobic glycolysis when the cellular requirement for NAD^+^ surpasses that for ATP [55].

Understanding lactate production and clearance provides insights into why cells may favor glycolysis over oxidative phosphorylation despite ample oxygen availability. Elevated circulating lactate levels can be transported between the liver and skeletal muscles for reconversion into glucose through the Cori cycle. Monocarboxylate transporters (MCTs) are instrumental in the transport of lactate among concentration gradients. Among the 14 recognized MCT isoforms, MCT1 and MCT4 have garnered significant attention because of their roles in the pathogenesis of various diseases [56]. MCT1 is widely expressed in various cell types and aids in lactate uptake or release, depending on the cellular metabolic needs, whereas MCT4 is predominantly expressed in cells with high glycolytic activity to export lactate. During acute ischemia, lactate accumulation decreases the pH, activating Na^+^/H^+^ exchange (NHE) proteins. This process leads to increased Ca^2+^–Na^+^ exchange and intracellular calcium overload [57]. Among patients with AMI, 24-h lactate levels and clearance rates are recognized as predictors of hospitalization-related mortality [58]. In patients with MI, elevated lactate levels stimulate the TGF-β pathway through the lactylation of the transcription factor SNAIL1, thereby enhancing the endothelial–mesenchymal transition (EndMT) and fibrosis [59]. Similarly, increased glycolysis and increased lactate levels in the early stages of MI can induce interleukin-1β/general control non-depressible 5 (GCN5)-mediated histone H3 lactylation at lysine 18 (Lys18), promoting the transcription of reparative genes [60].

In Ang II-induced cardiac remodelling, mitochondrial pyruvate uptake and mitochondrial respiration in CFs are disrupted because of the hyperacetylation of mitochondrial pyruvate carrier 2 (MPC2) at Lys19. This disruption leads to lactate accumulation and a metabolic shift from oxidative phosphorylation to aerobic glycolysis, which further increases lactate transport to CMs and aggravates CM hypertrophy [61]. Additionally, lactate exchange from CFs to CMs is regulated by CF-derived exosomes, which modulate glycolytic enzyme levels in CMs during age-induced HF [62]. Extracellular lactate has also been shown to stimulate TGF-β and induce the EndMT, leading to cardiac vascular fibrosis in a pH-independent manner [63]. On the other hand, lactate in CMs may play a protective role in HF. Decreased lactate production and reduced lactylation of the α-myosin heavy chain (α-MHC) at Lys1897 impair its interaction with titin, compromising cardiac structure and function. Increasing lactate levels in CMs through sodium lactate administration or by blocking the key lactate transporter has been shown to increase α-MHC lactylation, thereby alleviating HF [64].

In this section, we mainly summarized the role of crucial enzymes, including HK2, PFK-1 and PKM2, in cardiac fibrosis. Moreover, other glycolytic enzymes involved in fibrosis, such as the functional differences caused by the localization of ENO1, were investigated. Additionally, we discussed the lactate cycle, in which increased lactate levels can induce fibrosis through the lactylation of SNAIL1 and histone H3 during MI. The role of PKM2 in promoting fibrosis by activating CFs via the TGF-β/JAK2–STAT3 pathway and alleviating fibrosis by activating RAC1 in CMs in TAC-induced HF clearly differs. This difference may be attributed to cell type-specific functions or context-dependent signalling mechanisms. Future studies should focus on elucidating the context-dependent roles of glycolytic enzymes such as PKM2 and developing cell type-specific targeting strategies to achieve therapeutic benefits without off-target effects.

#### Lipid metabolism in cardiac fibrosis

Under pathological conditions such as obesity, diabetes, and myocardial ischemia, cardiac lipid metabolism becomes profoundly dysregulated. The uptake of FAs begins to surpass the capacity for β-oxidation, leading to detrimental intracellular lipid accumulation. This ectopic lipid deposition, known as lipotoxicity, is a key driver of cardiac fibrosis and remodelling. The mechanisms linking lipotoxicity to fibrosis are complex. Lipotoxicity extensively participates in the initiation and progression of cardiac fibrosis, contributing to worsened cardiac dysfunction [29, 65].

As a key transmembrane lipid transporter, CD36 orchestrates cardiac FA utilization. Its function is dynamically regulated by extensive PTMs, including phosphorylation, glycosylation, ubiquitination, and palmitoylation, as well as its reversible shuttling between the plasma membrane and intracellular compartments in response to specific signals [66]. O-GlcNAcylation has dual effects on cardiac function: acute increases are cardioprotective, whereas a chronic elevation induces myocardial injury [67]. In myocardial I/R injury, O-GlcNAc transferase (OGT) modifies CD36 at Ser195. This O-GlcNAcylation process stabilizes the CD36 protein and ultimately alleviates myocardial damage and fibrosis [68]. Additionally, the inhibition of CD36 palmitoylation can improve post-MI cardiac function and fibrosis through a dual mechanism. First, it reduces the activity of plasma membrane-localized CD36 to alleviate lipid metabolism disorders; second, it increases the activity of the mitochondrial CD36–phosphoglycerate mutase 5 (PGAM5) signalling axis, thereby increasing mitophagy efficiency and mitigating mitochondrial dysfunction [69]. Diabetic cardiomyopathy, a special form of non-ischemic cardiac dysfunction, is characterized by diastolic impairment, myocardial fibrosis, and frequent ventricular hypertrophy. These structural and functional alterations are driven primarily by core metabolic disturbances, including mitochondrial oxidative stress, insulin resistance, and aberrant lipid metabolism [70]. In the lipid-overloaded heart, CD36-mediated lipid uptake and accumulation are driven primarily by increased translocation rather than elevated expression. The core mechanism involves impaired endosomal acidification due to lipid-induced v-ATPase disassembly. This defect increases basal CD36 translocation to the membrane and concurrently renders it unresponsive to insulin [66]. The NAD^+^ precursor nicotinamide mononucleotide (NMN) can stimulate glycolytic enzymes to bind and reassemble v-ATPase. This activity preserves endosomal acidification, thereby suppressing CD36-mediated lipid accumulation and its deleterious interaction with Toll-like receptor 4 (TLR4). Consequently, NMN treatment prevents the downstream pathological cascade of inflammation, fibrosis, cardiac dysfunction, and systemic insulin resistance [71]. Furthermore, in diabetic hearts, the transcription factor forkhead box O1 (FoxO1) increases the expression of the zinc finger DHHC-type palmitoyltransferase 4 (zDHHC4), which in turn mediates CD36 S-acylation, thereby driving lipotoxicity and fibrosis [72].

In cardiac fibrosis, ACLY can couple metabolic flux to epigenetic regulation by mediating histone acetylation, thereby controlling myoFb differentiation. Studies have revealed that ACLY can translocate to the nucleus, where it functions as a crucial epigenetic coregulator by providing acetyl-CoA for histone 3 modification at lysine 27 (H3K27). This nuclear ACLY activity is indispensable for sustaining the expression of fibrotic genes and the persistent myoFb phenotype, making it a compelling therapeutic target [73]. Similarly, in Ang II-induced cardiac fibrosis, ACLY can promote H3K9 and H3K27 acetylation at fibrotic gene promoters, thereby driving their expression in CFs [74].

This section summarizes the functions of CD36 and ACLY in cardiac fibrosis, focusing particularly on how their PTMs and subcellular localization critically regulate lipid metabolism. The transition of O-GlcNAcylation from a cardioprotective role to a pathological role remains a central unresolved question in cardiac metabolism. Current evidence suggests that this functional shift is not merely due to a chronic increase in global O-GlcNAc levels but rather involves several sophisticated regulatory mechanisms, including site-specific modification and crosstalk with other PTMs. Future research should focus on mapping specific O-GlcNAcylated sites across various physiological and pathological conditions while developing technologies to precisely monitor and manipulate site-specific modifications in a temporally controlled manner. Moreover, ferroptosis is inseparable from lipid metabolism, as it results from the aggregation of labil iron and the peroxidation of membrane phospholipids enriched with polyunsaturated fatty acids (PUFAs). Notably, the link between this form of lipid metabolic abnormality and cardiac fibrosis has been well established in recent systematic reviews and thus will not be detailed in this context [29, 75].

### Pulmonary fibrosis

**Fig. 4:**
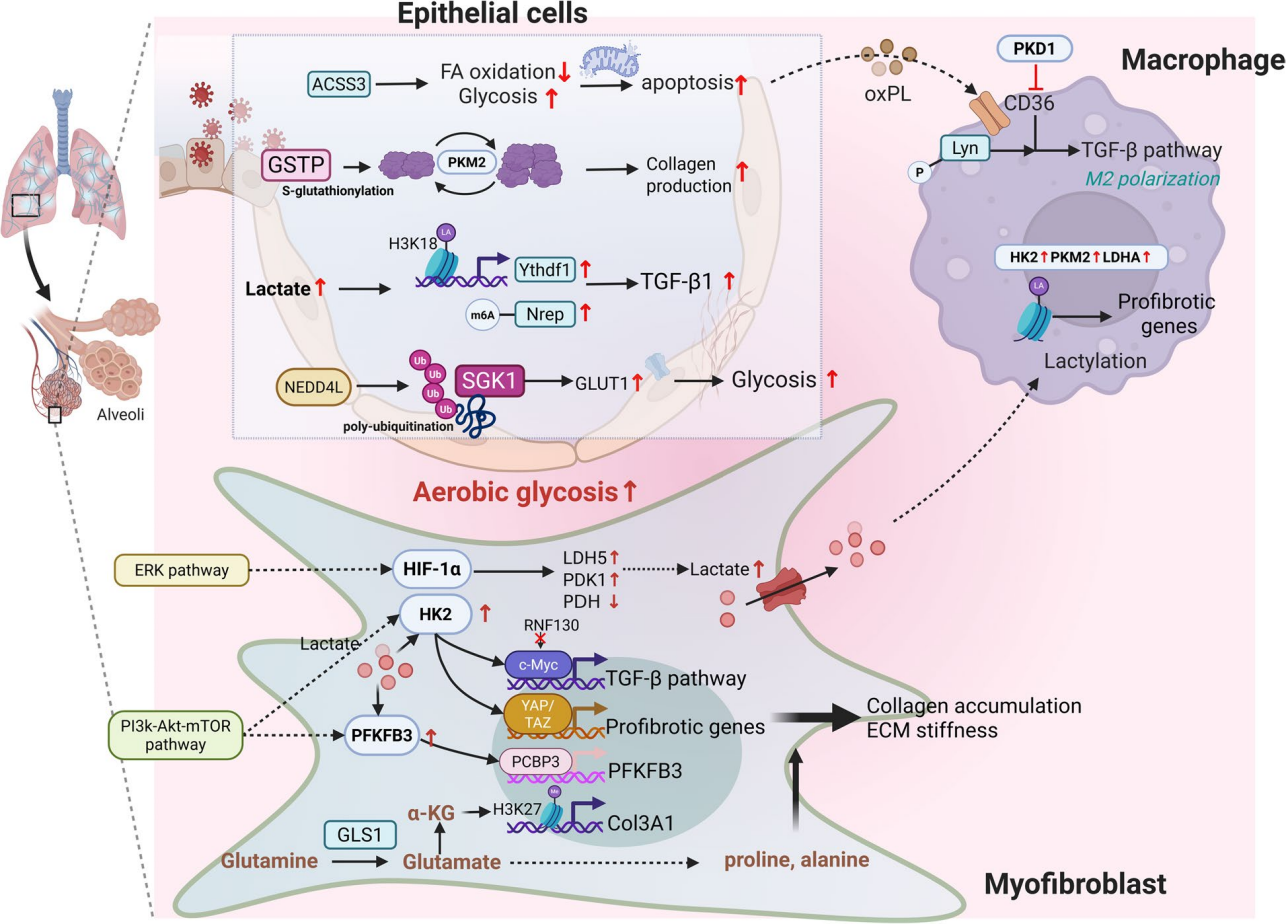
Enzymatic regulation drives metabolic reprogramming during pulmonary fibrosis. In pulmonary fibrosis, key glycolytic enzymes, including HK2, PKM2, LDH5, PDK1, PDH, and PFKFB3, are dysregulated, promoting a lactate-producing phenotype and fibroblast activation. This metabolic shift is governed by specific PTMs and signalling pathways. Concurrently, glutaminolysis and lipid metabolism are reprogramming in lung epithelial cells, fibroblasts, and macrophages. These three cell types form a metabolically coupled network through the exchange of intermediate metabolites, collectively driving ECM production and disease progression. PDK1, pyruvate dehydrogenase kinase 1; GSTP, glutathione S-transferase P; ERK, extracellular signal-regulated kinase; HIF-1α; hypoxia-inducible factor-1α; m6A, N6-methyladenosine; SGK1, serum/glucocorticoid-regulated kinase 1; RNF130, ring finger protein 130; YAP/TAZ, Yes-associated protein (YAP) and transcriptional co-activator with PDZ-binding motif (TAZ); PCBP3, Poly (RC) Binding Protein 3; GLS1, glutaminase-1; PKD1, protein kinase D1 (Figure was created with Biorender.com)

Pulmonary fibrosis is a major clinical outcome of various chronic respiratory diseases triggered by factors such as allergens, pathogenic microbes, and environmental particulates. It is characterized by scarring and thickening of the interstitial tissue of the lung, which impairs gas exchange (Fig. 4). Notably, idiopathic pulmonary fibrosis (IPF) is the most common form of interstitial pulmonary fibrosis with an unknown etiology [76]. Although the pathogenesis of IPF is highly complex, the dysfunction of Type II alveolar epithelial cells (AEC2s), which account for approximately 5% of the alveolar surface area and are highly metabolically active, is considered a central mechanism in IPF development [77]. The reprogramming of glucose, fatty acid, and amino acid metabolism in AECs during pulmonary fibrosis affects critical cellular processes, including energy generation and cell survival [78].

#### Glycolysis in pulmonary fibrosis

Fibrotic areas in the lungs of patients with IPF exhibit increased glycolytic activity, with the elevated glycolytic rates strongly correlated with reduced pulmonary function and increased mortality [79]. In addition, lactate mediates cell–cell interactions in IPF, promoting the activation of profibrotic signalling pathways and the progression of pulmonary fibrosis [80].

Studies have shown that compared with control cells, primary epithelial cells isolated from patients with IPF exhibit increased glycolysis [81]. ROS derived from glycolysis activate the NLRP3 inflammasome in lung epithelial cells, exacerbating lung injury and promoting the progression of lung injury [82]. Dysregulated lactate metabolism in AEC2s has been implicated in increased mitochondrial ROS generation and subsequent mitochondrial DNA damage [80]. Elevated lactate levels also promote the lactylation of H3K18 and regulate the transcription of the N6-methyladenosine (m6A) reader Ythdf1, which increases TGF-β1 secretion induced by neuronal protein 3.1 (NREP) and promotes the fibroblast-to-myofibroblast transition (FMT) [83]. The dimeric form of PKM2 has been shown to increase the shunting of glycolytic intermediates towards the serine synthesis pathway, leading to increased glycine and collagen production. Conversely, the tetrameric form of PKM2 inhibits this metabolic shift [84]. In lung epithelial cells, glutathione S-transferase P (GSTP) can disrupt the tetramer of PKM2 through the S-glutathionylation of cysteine to induce dimer formation, disrupting the canonical glycolytic function of PKM2 [85]. Moreover, the interplay between PKM2 and integrin β3 is pivotal for the metabolic reprogramming underlying aerobic glycolysis in pulmonary fibrosis [86]. In addition, serum/glucocorticoid-regulated kinase 1 (SGK1), which is highly homologous to a second messenger, upregulates glucose transporter 1 (GLUT1) and promotes glycolysis. Recent findings indicate that SGK1 degradation in epithelial cells or Fbs is mediated by NEDD4-Like E3 Ubiquitin Protein Ligase (NEDD4L) through Lys48-linked polyubiquitination and that this process can be inhibited by a high-fat diet (HFD) [87]. HFD-induced pulmonary fibrosis can be mitigated by the SGK1-specific inhibitor EMD638683.

Enhanced glycolysis is also observed in Fbs derived from the lungs of patients with IPF or in animal models of pulmonary fibrosis. The evidence suggests that TGF-β induces the expression of GLUT1 in Fbs and mesenchymal cells, both in vitro and in vivo, which in turn increases glucose uptake and the activation of glycolysis [88, 89]. The inhibition of glycolysis has been shown to suppress TGF-β1-induced Fb activation, as evidenced by reduced production of fibrotic markers and decreased cell proliferation, highlighting the role of dysregulated glycolysis in Fb activation [90]. HK2, the first catalytic enzyme involved in glucose metabolism, is enriched in primary human lung Fbs and upregulated in IPF patients. The transcription factor c-Myc has been shown to bind to HK2, thus mediating both the canonical and noncanonical TGF-β pathways. Moreover, the degradation of c-Myc, which is mediated by ring finger protein 130 (RNF130), suppresses glycolysis and Fb activation [91]. The kinase activity of HK2 also influences the expression of the paralogue Yes-associated protein (YAP) and its transcriptional coactivator PDZ-binding motif (TAZ), thereby increasing ECM stiffness and the expression of profibrotic genes [92]. In bleomycin-induced pulmonary fibrosis, HK2 has been identified as a downstream target of the PI3K/AKT/mTOR (mammalian target of rapamycin) pathway. Suppressing aberrant HK2 activity can stabilize energy metabolism and alleviate collagen deposition [93]. Intriguingly, the PI3K/AKT/mTOR signalling pathway also regulates PFKFB3, thus promoting collagen accumulation and accelerating pulmonary fibrosis [94]. Additionally, in bleomycin-induced pulmonary fibrosis, the PFKFB3 protein level is increased through its interaction with Poly (RC) Binding Protein 3 (PCBP3), which increases its translational efficiency [95]. During the progression of pulmonary fibrosis, lactate is thought to form a positive feedback loop, leading to increased expression of HK2 and PFKFB3, which further enhance aerobic glycolysis and Fb activation [96]. In addition, the disheveled-associated antagonist of β-catenin 2 (DACT2) facilitates lysosome-mediated LDHA degradation, thereby inhibiting glycolysis and the differentiation of myoFbs in pulmonary fibrosis [97]. HIF-1α has been proven to be a crucial factor mediating pulmonary fibrosis. The extracellular signal-regulated kinase (ERK) pathway can stabilize the HIF-1α protein and impede its degradation, thus upregulating glycolysis, fibroblast proliferation, and ECM production [98]. Furthermore, HIF-1α facilitates the differentiation of myoFbs by mediating the TGF-β1-induced production of LDH5 (entirely composed of LDHA), which subsequently increases lactate levels [80]. HIF-1α also induces pyruvate dehydrogenase kinase 1 (PDK1), which in turn inhibits the conversion of pyruvate to acetyl-CoA by phosphorylating PDH and redirects metabolism towards lactate [99]. Interestingly, in primary human lung Fbs, aerobic glycolysis and fibroblast activation are not always congruent, suggesting context-dependent mechanisms that remain to be elucidated. Compound 408, an inhibitor of LDH5, can significantly reduce the metabolic shift towards aerobic glycolysis without affecting the production of fibrosis-associated markers such as fibronectin and collagen. These findings imply that glycolytic dysregulation may not be the sole driver of fibrosis, highlighting the need for further investigations into metabolic reprogramming in diverse cell types within the IPF microenvironment to identify additional therapeutic targets [100].

In fibrotic lungs, alveolar macrophages typically exhibit predominantly profibrotic M2-like characteristics, with increased glycolysis and upregulated the expression of key glycolytic enzymes, including HK2, PKM2 and LDHA [101]. Recent evidence further reveals that lactate plays a critical role in this process. Specifically, PM2.5 exposure induces intracellular lactate accumulation in macrophages, which in turn promotes H3K18 lactylation at the promoter of the ubiquitin ligase CHIP (carboxyl terminus of Hsp70-interacting protein), thereby suppressing CHIP expression. Reduced CHIP impairs the ubiquitination and degradation of TGF-β1, leading to increased TGF-β1 secretion from macrophages and ultimately exacerbating pulmonary fibrosis [102]. This study uncovers a novel mechanism linking macrophage glycolysis-derived lactate to post-translational regulation of a master fibrotic cytokine through epigenetic crosstalk. Lactate secreted by myoFbs enters the extracellular milieu and is transported into macrophages, where it induces p300-mediated histone lactylation, a process that affects chromatin structure. This modification further upregulates fibrosis-related gene expression in macrophages, thereby promoting the progression of pulmonary fibrosis [103].

In this section, we have reviewed the role of glycolysis in lung epithelial cells, Fbs, and macrophages in fibrosis. Generally, increased glycolysis or elevated lactate levels can promote the progression of pulmonary fibrosis. However, the field relies heavily on a bleomycin-induced mouse model, which reflects acute lung injury rather than the chronic progression of human IPF, limiting the translational relevance of the existing findings. A key unresolved question remains the precise characterization of metabolic dependencies and crosstalk among diverse cell types within the authentic human IPF microenvironment. Therefore, future research should focus on using human IPF tissues and coculture systems to elucidate cell-specific metabolic pathways and identify promising therapeutic targets.

#### Lipid metabolism in pulmonary fibrosis

In addition to their basic physiological functions in the formation of cell membranes, a distinct circulating lipid metabolome has been identified in pulmonary fibrosis. Elevated levels of non-esterified FAs, acylcarnitines, and ceramides indicate a heightened catabolic state that promotes lipid mobilization and breakdown [104].

In pulmonary fibrosis, AEC2s exhibit a decrease in lipid homeostasis, characterized by a decrease in the transcription of the genes involved in key lipid metabolic pathways, such as fatty acid synthesis and β-oxidation [105]. In addition, damaged AEC2s are considered the primary source of the characteristic lipid abnormalities in the serum of patients and mouse IPF models, and these released lipids directly participate in driving the fibrotic process [106]. Apoptotic AEC2s can release oxidized phospholipids (oxPL), which is scavenged by CD36 on the surface of macrophages. This uptake of oxPL by CD36 directly induces its interaction with the Src kinase Lyn, leading to the phosphorylation and activation of Lyn. This CD36–Lyn kinase complex is a critical signalling node, as the pharmacological inhibition of Lyn with bafetinib specifically ablates the subsequent upregulation of TGF-β1 in macrophages, thereby disrupting the fibrotic cascade [107]. Subsequent studies have confirmed that CD36 promotes pulmonary fibrosis by directly binding to TGF-β1 and driving M2 macrophage polarization. However, a key regulatory mechanism involving the G protein-coupled receptor 40 (GPR40)/protein kinase D1 (PKD1) signalling axis has been identified. Activation of the fatty acid-sensitive kinase PKD1 initiates a downstream cascade that suppresses CD36 expression, thereby effectively alleviating the fibrotic process [108]. Research has revealed that the interplay between glycolysis and fatty acid metabolism is centrally involved in pulmonary fibrosis. The downregulation of acetyl-CoA synthetase short-chain family member 3 (ACSS3) promotes a shift towards glycolysis while suppressing CPT1A-mediated FAO. This metabolic imbalance disrupts mitochondrial homeostasis, leading to dysfunctional dynamics, oxidative stress, impaired mitophagy, and AEC2 apoptosis, which collectively drive fibrosis progression [109].

In summary, pulmonary fibrosis is driven by impaired lipid metabolism in AEC2s and macrophages. Damaged AEC2s release lipids, including oxidized phospholipids, which are internalized via CD36 on macrophages. This process triggers a TGF-β-mediated profibrotic cascade. Conversely, the GPR40–PKD1 axis suppresses CD36, mitigating fibrosis. Concurrently, a metabolic shift towards glycolysis, driven by ACSS3 downregulation, further disrupts mitochondrial function and promotes AEC2 apoptosis. Research has indicated that CD36 promotes pulmonary fibrosis through at least two distinct signalling pathways: one initiated by the uptake of oxidized phospholipids that activate the Lyn/TGF-β1 axis in macrophages and another involving direct binding to TGF-β1 to drive M2 macrophage polarization. However, whether these two profibrotic mechanisms are interconnected, synergistic, or context dependent remains unclear. The key methodological limitation in the field is the reliance mostly on pharmacological inhibitors, such as bafetinib, to establish the role of these pathways. The potential off-target effects of these compounds and the cell-type-specific functions of CD36 remain inadequately explored. An in-depth understanding of these processes is a prerequisite for developing highly specific CD36-targeted therapies that can inhibit its pathological functions without disrupting its essential physiological role in lipid metabolism. In addition, the convergent effects of ER stress, mitochondrial injury, and autophagy on AEC2 lipid metabolism are well established [77]. Therefore, this manuscript does not elaborate on their essential functions in maintaining homeostasis and limiting fibrotic remodelling.

#### Glutamine metabolism in pulmonary fibrosis

Glutamine metabolism is now recognized as a key contributor to pulmonary fibrosis. Metabolic intermediates of glutamine orchestrate critical profibrotic processes, including Fb activation and collagen production [110]. Concurrently, glutamine-dependent folate metabolism, optimized by polyglutamylation, secures the nucleotide supply and redox balance, thereby collectively enabling the persistent activation of fibrogenic cells.

In pulmonary Fbs, glutamine-derived glutamate fuels collagen synthesis by generating proline and alanine. While inhibiting glutamate production by decreasing the expression of GLS1 suppresses this process, alternative metabolic pathways involving glutamic-pyruvic transaminase 2 (GPT2) can bypass this blockade by regenerating glutamate and alanine, thereby sustaining collagen deposition [111]. In addition to Fbs, glutamine is crucial for the repair of AEC2s in response to lung injury [112]. GLS1 expression is regulated not only by TGF-β but also by Caveolin-1 (CAV1), and its mimetic peptide CSP7 exerts an antifibrotic effect [113]. Studies have indicated that the context-dependent effects of glutamine metabolism are partly mediated by α-KG. This metabolite not only partially rescues the deficits caused by glutamine deprivation but also directly regulates the fibroblast phenotype through the epigenetic modification of H3K27me3 at specific gene loci, such as the alpha 1 chain of type III collagen (Col3A1) [114]. Likewise, through its metabolite α-KG, glutamine also prevents apoptosis by sustaining the Jumonji domain-containing protein-3 (JMJD3) histone demethylase activity to repress the expression of H3K27me3-marked survival genes and augment cytoprotection by improving mitochondrial respiration [115].

In pulmonary fibrosis, glutamine metabolism orchestrates a range of pleiotropic effects, including Fb activation, collagen deposition, and epigenetic modulation. A crucial finding is that the inhibition of the primary enzyme GLS1 can be bypassed via the GPT2 pathway, sustaining collagen production. This result highlights a major challenge: the presence of redundant metabolic routes that can compensate for single-enzyme inhibition, potentially leading to therapeutic resistance. Future therapeutic strategies must advance beyond single-pathway inhibition, and systematic mapping of the metabolic network to identify nonredundant nodes is essential. In addition, as previously detailed in the literature, amino acids, including arginine and proline, play critical roles in pulmonary fibrosis; therefore, their functions are not elaborated upon in this discussion [110].

### Liver fibrosis

**Fig. 5:**
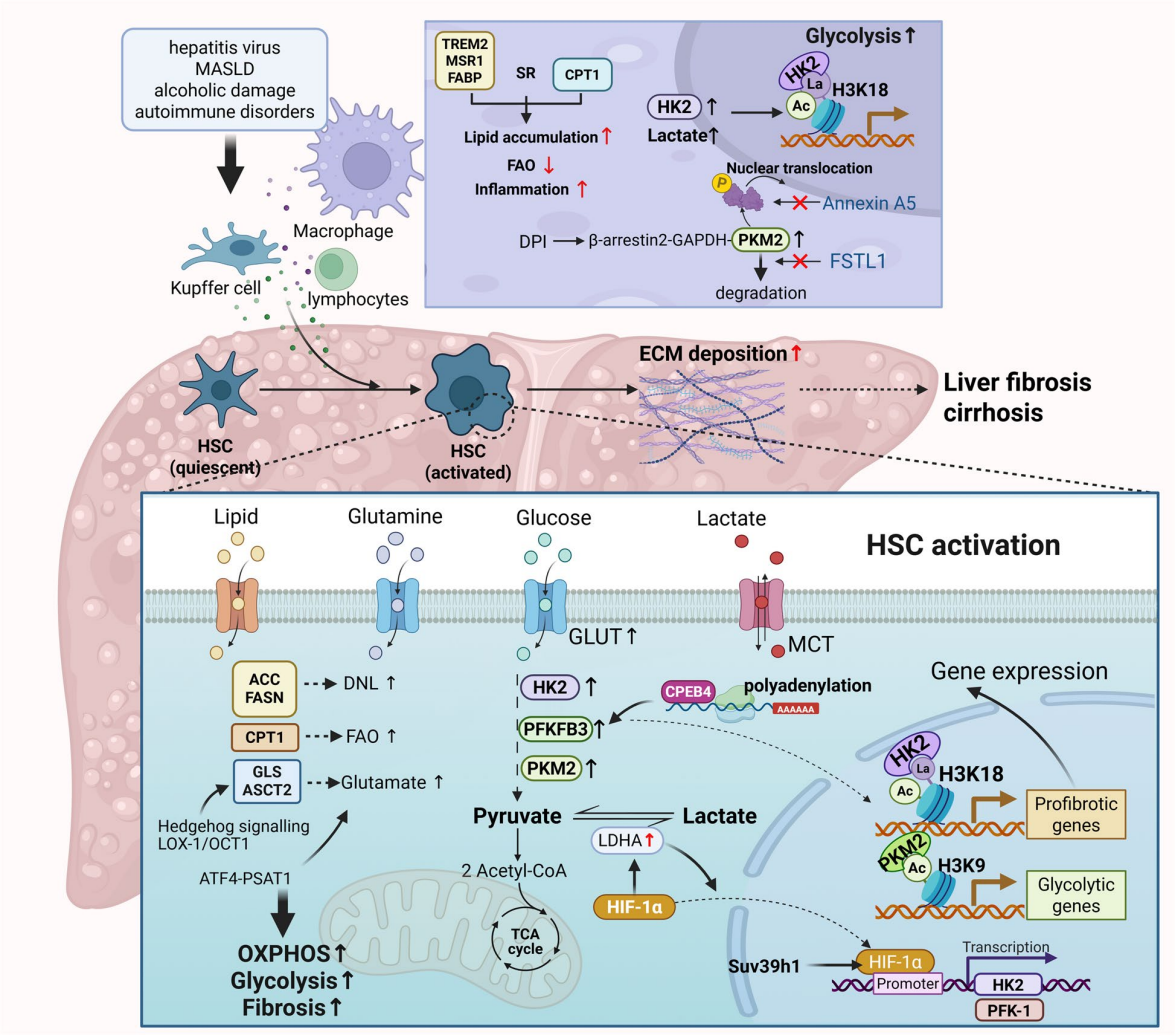
The Enzymatic regulation drives metabolic reprogramming in liver fibrosis. Upon activation, hepatic stellate cells (HSCs) exhibit concurrent upregulation of glycolysis and FAO. Glycolytic enzymes HK2, PFKFB3, and PKM2 drive a metabolic shift, with HK2 promoting histone lactylation at lysine 18 and PKM2’s dimeric form enhancing H3K9ac to sustain profibrotic signaling. Simultaneously, increased CPT1A-mediated FAO provides energy and biosynthetic precursors, supporting the activated, proliferative HSC phenotype. In macrophages, follistatin-like protein 1 (FSTL1) stabilizes PKM2 to amplify fibrotic responses, while lipid uptake via receptors like TREM2 and MSR1 leads to lipid accumulation and proinflammatory polarization. Annexin A5 counteracts HSC activation by promoting the tetrameric form of PKM2, underscoring the interplay between glycolytic and lipid pathways in driving liver fibrosis. MASLD, metabolic dysfunction-associated steatotic liver disease; MCT, monocarboxylate transporters; Suv39h1, suppressor of variegation 3–9 homolog 1; TREM2, triggering receptor expressed on myeloid cells-2; MSR1, macrophage scavenger receptor 1; DNL, de novo lipogenesis (Figure was created with Biorender.com)

Chronic liver diseases resulting from sustained injury, such as exposure to hepatitis virus, hepatotoxins, and autoimmune disorders, lead to a superfluous wound-healing response, resulting in ECM accumulation and liver fibrosis [116]. In response to signals from liver injury and immune cells, hepatic stellate cells (HSCs) are activated and transdifferentiated into a myofibroblast-like phenotype characterized by increased proliferation, migration, and ECM production. Persistent accumulation and disorganized crosslinking of ECM components disrupt cellular niches and exacerbate liver dysfunction, leading to liver cirrhosis, chronic liver failure, and an increased risk of hepatocellular carcinoma (Fig. 5). Under homeostatic conditions, quiescent HSCs function as non-parenchymal pericytes to regulate sinusoidal blood flow. Moreover, as the primary fibrogenic cell population in the liver, HSCs exhibit remarkable plasticity, enabling them to undergo metabolic remodelling, including increased glycolysis, lipid droplet mobilization, and glutaminolysis [117].

#### Glycolysis in liver fibrosis

Upon activation, HSCs upregulate enzymes associated with glycolysis, such as HK2, PFKFB3 and PKM2, further sustaining their profibrotic phenotype and accelerating fibrosis progression [16, 118]. Recently, HK2 has been demonstrated to induce histone lactylation at lys18 of histone 3 (H3K18la), thus mediating the persistent activation of HSCs in liver fibrosis [119]. The deletion of HK2 reduces lactate production and H3K18la levels, thus leading to HSC inactivation. A competitive dynamic has been observed between lactylation and acetylation at the H3K18 residue, which may influence chromatin accessibility and gene expression. This competition provides additional insights into how class I histone deacetylases (HDAC) inhibitors suppress HSC activation by increasing histone acetylation levels [120]. Additionally, the suppressor of variegation 3–9 homolog 1 (Suv39h1), a lysine methyltransferase, has been reported to facilitate HIF-1α binding to the promoter of HK2, thus accelerating glycolysis and subsequent HSC activation [121]. In both mouse and human fibrosis models, the protein levels, but not the mRNA levels, of PFKFB3 are increased in activated HSCs, indicating the presence of PTMs. Indeed, the 3'-untranslated region of the PFKFB3 mRNA binds to the cytoplasmic polyadenylation element binding protein 4 (CPEB4). This interaction facilitates cytoplasmic polyadenylation, which in turn induces the translation of PFKFB3 [122]. The ability of PKM2 to activate HSCs is also subject to its conformational state. Specifically, the dimeric form, rather than the tetrameric form, increases the histone H3 acetylation at lysine 9 (H3K9ac), which facilitates glycolysis and aggravates liver fibrosis [123]. In addition to HSCs, recent single-cell omics studies have revealed pivotal roles for Kupffer cells (KCs) and monocyte-derived macrophages in liver fibrosis. These cells exhibit extensive heterogeneity, with distinct subpopulations orchestrating various stages of fibrogenesis [124]. PKM2 expressed in monocyte-derived macrophages also plays a pivotal role in liver fibrosis. Follistatin-like protein 1 (FSTL1) can directly interact with PKM2, which inhibits its ubiquitin-mediated degradation. This interaction promotes the phosphorylation and subsequent nuclear translocation of PKM2, thereby amplifying its role in the fibrotic response [125]. Furthermore, the phosphorylation of monomeric/dimeric PKM2 at tyrosine 105 (Tyr105) is critical for its nuclear translocation and glycolytic reprogramming activity. Annexin A5 can interact with surrounding amino acid residues, such as aspartic acid 101 (Asp101), leucine 104 (Leu104), and arginine 106 (Arg106), thereby inhibiting phosphorylation and inducing the formation of PKM2 tetramers [126]. In addition to PKM2, in metabolic dysfunction-associated steatotic liver disease (MASLD), macrophages exhibit a vicious positive feedback loop driven by HK2 and histone lactylation (H3K18la). Elevated HK2 expression increases glycolysis and lactate production, which in turn promotes the H3K18la modification. This lactate-derived histone mark activates the transcription of glycolytic genes, further amplifying glycolytic flux and driving the pro-inflammatory M1 polarization of macrophages. The resulting HK2/glycolysis/H3K18la cycle perpetuates metabolic dysregulation and inflammatory activation in macrophages. Myeloid-specific HK2 deletion or pharmacological inhibition of HIF-1α can disrupt this loop and alleviate fibrotic progression in individuals with MASLD models [127]. KCs, liver-resident macrophages, also play crucial roles in maintaining hepatic homeostasis and shaping inflammatory responses. Recent studies have shown that the activation of the G-protein coupled receptor 3 (GPR3)/β-arrestin2/PKM2 axis can trigger metabolic reprogramming in KCs, rapidly shifting their energy metabolism from oxidative phosphorylation to glycolysis. This transition involves the formation of a β-arrestin2/GAPDH/PKM2 supercomplex and the nuclear translocation of PKM2, increasing glycolytic gene expression. In vivo, enhancing this pathway can suppress inflammation in KCs and ameliorate HFD-induced obesity and fibrosis in mice. Disease-associated macrophages in human fatty liver biopsies also exhibit a shift towards glycolytic gene expression and reduced inflammatory gene transcription in response to GPR3 activation [128].

In a healthy liver, lactate is predominantly taken up for gluconeogenesis, with the liver accounting for approximately 70% of systemic lactate clearance [129]. Recently, LDHA has emerged as a critical regulator of lactate metabolism. The N-terminal glycine 28 (Gly28) residue of LDHA can interact with HIF-1α, aiding its nuclear translocation and promoting the transactivation of glycolytic enzymes, such as HK2 and PFK-1. LDHA could also be upregulated by HIF-1α, suggesting a potential positive feedback loop involving the Wnt/β-catenin pathway [130]. In addition to HIF-1α, Kruppel-like factor 5 (KLF5) has been shown to form a self-reinforcing feedback loop with LDHA, and disrupting this loop alleviates liver fibrosis [131]. Oroxylin A, a natural flavonoid, has been demonstrated to hinder glycolysis-mediated contraction in HSCs by suppressing LDHA expression [132].

The liver is a vital organ for energy metabolism and is the largest lactate-clearing organ in the body, and liver fibrosis involves the glycolytic reprogramming of HSCs, macrophages and KCs, thereby contributing to the development of profibrotic cell niches. Nevertheless, glycolytic remodeling appears to be cell-type specific, posing challenges for its therapeutic translation. The “competitive dynamic” between H3K18 lactylation and acetylation represents a fascinating regulatory mechanism. However, which specific genes are differentially regulated by this competition and how the balance ultimately directs profibrotic versus antifibrotic transcriptional regulation remain unclear. Future studies should focus on multiomics approaches to identify both intracellular and intercellular metabolic fluxes while also pinpointing key regulatory molecules that control these specific PTMs. In the following section, we briefly outline the remodelling of lipid and glutamine metabolism. Given that these pathways have been extensively discussed in the recent literature, they will not be examined in detail here [16].

#### Lipid metabolism in liver fibrosis

In their quiescent state, HSCs function as pericytes by storing retinoids in cytoplasmic lipid droplets to maintain tissue homeostasis. Upon liver injury, activated HSCs progressively deplete their retinoid reserves and instead accumulate polyunsaturated triglycerides. This alteration in the lipid profile facilitates the transformation of cells into myoFbs, with new lipid droplets providing metabolic support for fibrogenic activities [133]. Concurrently, activated HSCs also present enhanced de novo lipogenesis (DNL). The inhibition of DNL enzymes such as ACC and FASN can decrease fibrogenic gene expression and reduce oxidative phosphorylation and glycolysis in both rodent and human HSCs [134]. CPT1A, the rate-limiting enzyme for mitochondrial FAO, is upregulated in activated HSCs from patients with MASH and animal models and is correlated with fibrosis severity. Its expression is also elevated in TGF-β1- or serum-stimulated primary human HSCs in vitro. Both pharmacological and genetic knockdown of CPT1A suppress HSC activation by reducing mitochondrial metabolic activity, whereas CPT1A upregulation by saturated FAs and oxidative stress promotes the expression of fibrogenic genes [135]. In addition to HSCs, immune cells also undergo lipid metabolic reprogramming that drives fibrosis in individuals with MASLD. Macrophages upregulate lipid-sensing receptors, including triggering receptor expressed on myeloid cells-2 (TREM2) and macrophage scavenger receptor 1 (MSR1) to increase lipid uptake, initially alleviating hepatocyte steatosis [136]. However, this adaptation leads to intracellular lipid accumulation, driving proinflammatory polarization. Specifically, MSR1 facilitates saturated FA uptake, exacerbating inflammatory cytokine release, whereas fatty acid-binding protein 4 (FABP4) mediates FA absorption and promotes macrophage polarization via ceramide accumulation and mitochondrial dysfunction. These lipid-handling mechanisms underscore the dual roles of macrophages in early protection and later inflammation [137]. Moreover, macrophages present increased uptake of cholesterol and oxidized lipoproteins through scavenger receptors, leading to lysosomal cholesterol accumulation, foam cell formation, and inflammasome activation. Mitochondrial dysfunction exacerbates inflammation, as palmitic acid induces mitochondrial DNA leakage and suppresses FAO, whereas CPT1A inhibition alleviates ER stress. Lipid-metabolizing enzymes such as monoacylglycerol lipase regulate prostaglandin synthesis and macrophage polarization; the inhibition of these enzymes reduces inflammation but may impair liver regeneration [124]. TREM2^+^ lipid-associated macrophages (LAMs) initially increase lipid clearance but shift to a proinflammatory phenotype following chronic injury, resulting in the production of ROS and lipid peroxidation products that influence polarization and disease progression towards fibrosis. Macrophages undergo metabolic reprogramming in MASLD, creating a proinflammatory loop via lipid accumulation, oxidative stress, and mitochondrial damage, which collectively promote steatohepatitis, fibrosis and advanced liver disease [138, 139].

Macrophages play a dual role in liver fibrosis. They initially clear lipids via TREM2 and MSR1 to maintain homeostasis; however, upon chronic lipid exposure, these same pathways drive proinflammatory and profibrotic responses. The molecular switch governing this transition remains unknown. Future studies can focus on lineage-tracing methods to track lipid-accumulating cells throughout disease progression, aiming to identify key regulators that shift lipid metabolism from protective to pathogenic. Interestingly, FAO is suppressed in lipid-laden hepatocytes but is increased in activated HSCs, which utilize it for activation. This fundamental dichotomy necessitates the development of cell type-specific medicines that can precisely modulate FAO to suppress fibrosis without harming parenchymal cells.

#### Glutamine metabolism in liver fibrosis

Similar to cancer cells, activated HSCs also show glutamine addiction, relying on glutamine catabolism to generate α-KG and fuel the TCA cycle for biosynthesis. Glutamine metabolism drives HSC activation and liver fibrosis through key enzymes and transporters. Activated HSCs can upregulate GLS expression via Hedgehog signalling, converting glutamine to glutamate; its inhibition alleviates carbon tetrachloride (CCl₄)-induced liver fibrosis in mice [140]. Knockdown of the glutamine transporter ASCT2 in human HSCs reduces glutamate and α-KG levels, suppresses oxidative phosphorylation, and impedes activation. In a murine model of CCl₄-induced fibrosis, ASCT2 knockdown or pharmacological inhibition attenuates collagen deposition and HSC activation, whereas its overexpression exacerbates fibrosis [141]. As a master transcriptional regulator of amino acid metabolism, activating transcription factor 4 (ATF4) critically controls serine/glycine biosynthesis by upregulating key enzymes such as phosphoserine aminotransferase 1 (PSAT1). PSAT1 not only functions in serine synthesis but also acts as a glutamine transaminase, thereby bridging glutaminolysis to glycine production. This ATF4–PSAT1 axis forms a metabolic network where glutamine catabolism fuels serine/glycine anabolism, ultimately supporting collagen synthesis in HSCs [142]. Lipid and glutamine metabolism are closely related in liver fibrosis. For instance, the scavenger receptor lectin-like oxidized LDL receptor 1(LOX-1), which is upregulated in HSCs, promotes cholesterol accumulation and the recruitment of the transcription factor organic cation transporter 1 (OCT1), also known as SLC22A1. This LOX-1/OCT1 complex transcriptionally activates GLS1, thereby linking cholesterol overload to glutaminolysis. The disruption of this axis reduces GLS1 expression, impedes HSC activation, and alleviates fibrosis, thereby establishing the LOX-1/OCT1/GLS1 pathway as a potential therapeutic target in liver fibrosis [143].

During liver fibrosis, glycolytic remodeling is markedly increased in both HSCs and immune cells, serving as a central driver of fibrogenesis. This shift is intertwined with dysregulated lipid and glutamine metabolism, where intermediate metabolites reciprocally influence activation and inflammatory responses. A major limitation of current research is the predominant reliance on the CCl_4_-induced mouse model, which mostly reflects acute toxic liver injury rather than slow, metabolically driven MASH-related fibrosis in humans. This discrepancy may compromise the translational relevance of the reported findings. Moreover, therapeutic agents targeting these pathways must account for cell-specific effects and the temporal context, as metabolic dependencies evolve with disease progression.

### Renal fibrosis

**Fig. 6:**
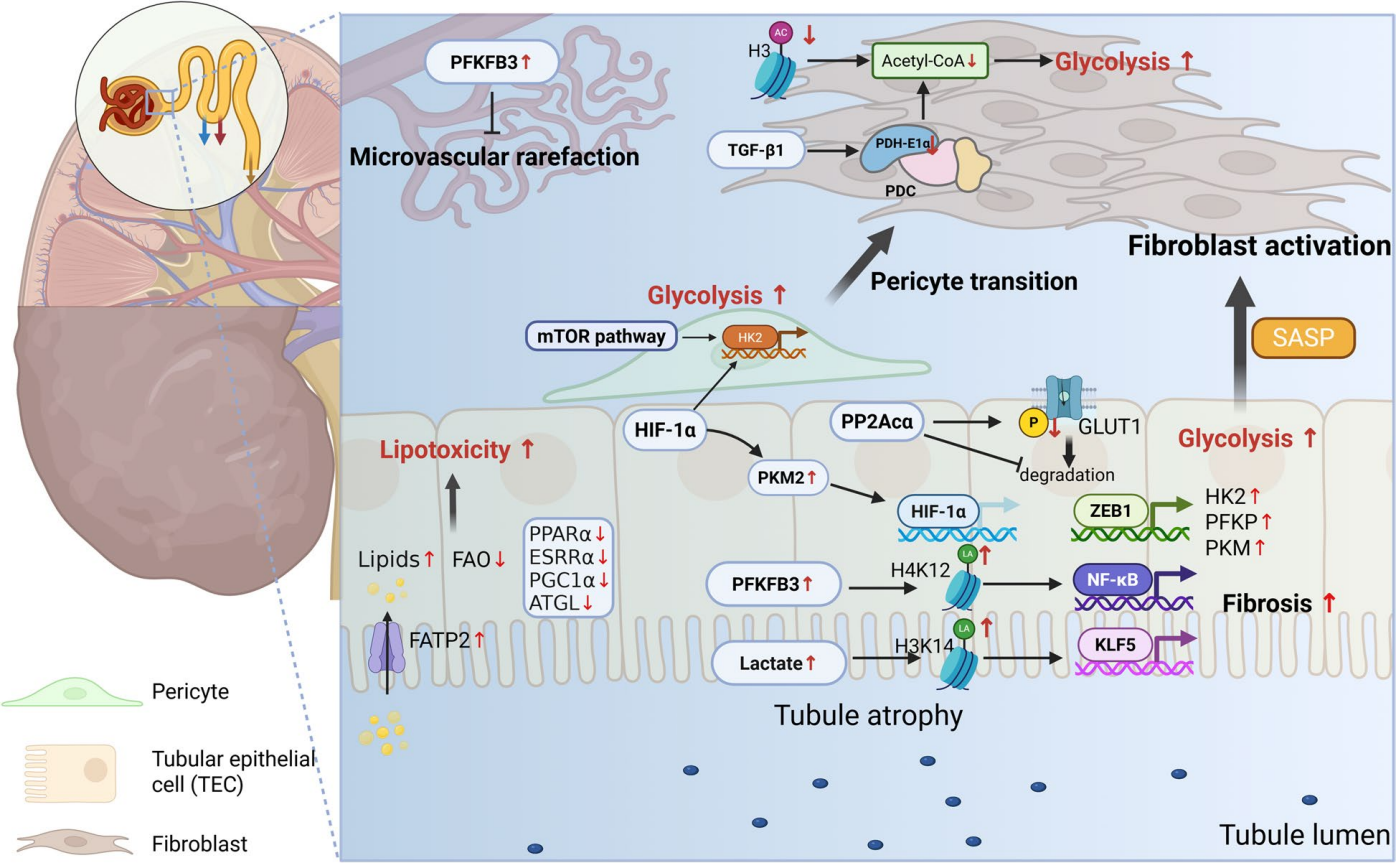
The Metabolic reprogramming in renal fibrosis is driven by enzyme regulation through transcription and PTMs. Tubular epithelial cells (TECs) exhibit increased aerobic glycolysis, driven by the upregulation of key glycolytic enzymes such as PKM2 and PFKFB3. These enzymes further lead to histone lactylation, contributing to the activation of the NF-kB cascade, HIF-1α, and KLF5 expression, all of which promote fibrotic progression. Concurrently, impaired FAO and enhanced lipid uptake in TECs lead to intracellular lipid accumulation, resulting in lipotoxicity that exacerbates cellular injury. Persistent TEC injury exhibits a senescent phenotype, accompanied by the secretion of profibrotic factors and a senescence-associated secretory phenotype (SASP), which contribute to fibroblast activation. In myofibroblasts (myoFbs), reduced histone H3 acetylation and diminished acetyl-CoA levels favor aerobic glycolysis over FAO. Additionally, activation of either the mTOR pathway or HIF-1ɑ enhances glycolysis by promoting HK2 expression, which leads to pericyte-myofibroblast transition (PMT). PDC, pyruvate dehydrogenase complex; KLF5, Kruppel-like factor 5; ZEB1, zinc finger E-box binding homeodomain 1; PPARα, peroxisome proliferator-activated receptor α; ESRRα, estrogen-related receptor α; PGC1α, the peroxisome proliferator-activated receptor γ coactivator 1α; ATGL, adipose triglyceride lipase (Figure was created with Biorender.com)

Following a series of insults, local kidney cells such as Fbs and pericytes are activated, which increases their contractility, promotes the secretion of inflammatory cytokines, and stimulates the production of ECM components, thereby initiating the wound-healing response. Within this context, fibrosis emerges as a consequence of dysregulated wound healing [144]. In particular, this process triggers a cascade of events that establish a distinct tissue microenvironment characterized by qualitative and quantitative changes in the ECM and the phenotypic transformation of surrounding cells, resulting in the formation of a fibrogenic niche [145]. Interestingly, these fibrotic areas usually exhibit well-defined boundaries containing mesenchymal cells, immune cells, and specific types of tubular epithelial cells (TECs) [146]. Fibrogenesis in the kidney is a stepwise process involving a series of intricate and dynamic cellular changes, including renal cell injury, inflammatory cell influx, myoFb activation, tubular atrophy, and a reduction in the microvasculature (Fig. 6).

Although the kidney is not traditionally considered a metabolic organ, it is among the body’s most energetically demanding organs, requiring substantial energy to filter metabolic waste, reabsorb essential nutrients, and maintain acid–base and fluid balance. In the kidney, ATP production is tailored to the specific energy demands of specific cell types. For instance, TECs, which are crucial for the energetically intensive process of metabolite reabsorption, primarily utilize FAs, lactic acid, and glutamine to generate ATP. In contrast, endothelial cells, myoFbs, and podocytes predominantly rely on glucose [147]. Spatiotemporal metabolic reprogramming acts as a central driver of renal fibrosis by coordinating profibrotic effector functions and interactions across diverse kidney cell types, from TECs and endothelial cells to stromal and immune cells, resulting in increased aerobic glycolysis and decreased FAO [148].

#### Glycolysis in renal fibrosis

In proximal TECs, elevated levels of PFKFB3 lead to a notable increase in histone lactylation, particularly on histone H4 at lysine 12 (H4K12la), which subsequently activates the NF-kB cascade and exacerbates fibrosis [149]. In addition to TECs, elevated PFKFB3 levels in endothelial and myeloid cells also promote glycolysis and contribute to renal fibrosis progression [150, 151]. However, a recent study revealed that increased PFKFB3 expression exerts a protective effect on peritubular endothelial cells. The overexpression of PFKFB3 can alleviate reduced glycolytic activity, thereby reversing microvascular rarefaction and mitigating fibrosis [152]. PFK-1 exists in three distinct isoforms: the muscle-specific form (PFKM), the liver-specific form (PFKL), and the ubiquitous form (PFKP). Suppressing the expression of PFKM or PFKP in TECs can prevent fibrosis induced by TGF-β [153, 154]. Additionally, in damaged tubular cells, protein phosphatase 2 A catalytic subunit α (PP2Acα) plays a key role in glucose metabolism by targeting GLUT1. In particular, PP2Acα dephosphorylates the critical Thr478 site on GLUT1, which in turn suppresses the TRIM21-mediated ubiquitination and degradation of GLUT1. This process facilitates glucose uptake and glycolysis [155]. In fibrotic kidney models, the phosphorylation of PKM2 is increased, and suppressing the expression of glycolysis-associated enzymes can significantly mitigate renal fibrosis [156, 157]. Canonical pathways, such as the mTOR and HIF-1α pathways, have also been shown to promote aerobic glycolysis in renal fibrosis. Rapamycin, an inhibitor of mTOR complex 1 (mTORC1), can suppress the proliferation of TECs and thereby ameliorate interstitial fibrosis [158]. Similarly, empagliflozin, an inhibitor of sodium glucose cotransporter 2 (SGLT2i), can inhibit HIF-1α-induced PKM2 dimer formation, thus normalizing aberrant glycolysis and inhibiting the EndMT in proximal TECs [159]. Interestingly, after its nuclear translocation, PKM2 also interacts with HIF-1α to shift cellular metabolism towards glycolysis, hence contributing to tubular damage [160]. Activation of either the mTOR pathway or HIF-1α increases glycolysis by promoting the expression of HK2, which in turn leads to pericyte–myofibroblast transition (PMT) [161, 162]. Specific transcriptional factors, such as zinc finger E-box binding homeodomain 1 (ZEB1), can regulate the expression of multiple glycolytic enzymes, including HK2, PKM2, and PFKP, thus inducing glycolytic reprogramming and exacerbating renal fibrosis [163]. In Fbs, histone H3 acetylation is significantly reduced, thereby decreasing the availability of the acetyl donor acetyl-CoA. This alteration ultimately preferentially triggers Fbs towards aerobic glycolysis [164].

The kidney also plays a critical role in lactate catabolism, which is essential for maintaining lactate homeostasis. Indeed, elevated urinary lactate levels and increased expression of LDHA in human kidneys are indicators of advanced renal fibrosis and are correlated with a poor prognosis [165]. Increased levels of lactate can upregulate histone H3 lysine 14 lactylation (H3K14la), subsequently facilitating Kruppel-like factor 5 (KLF5) expression and accelerating renal fibrosis [166]. Upon injury, the level of the PDH-E1α subunit of the pyruvate dehydrogenase complex (PDC) is reduced in Fbs, increasing the susceptibility of the PDC to phosphorylation and subsequent inactivation. This alteration results in decreased acetyl-CoA production and a metabolic shift towards glycolysis [167]. In addition, hyperacetylation of Lys385 in PDH-E1α in TECs also reduces PDC activity. Restoring sirtuin3 activity could counteract this effect by inhibiting glycolysis and alleviating fibrosis [168]. In the context of renal fibrosis, myoFbs exhibit elevated levels of WW domain-containing E3 ubiquitin protein ligase 2 (WWP2). The deletion of WWP2 reduces the reliance on glycolysis, prompting cells to utilize FAs as alternative energy sources. This metabolic rewiring can also upregulate the PPP, enhancing G6PDH activity and accelerating PPP flux [169].

In this section, we mainly summarized the regulation of glycolysis in the cellular components of the fibrotic niche in kidneys, including TECs and myoFbs. The intriguing dual role of PFKFB3 poses a key challenge for therapy: it promotes fibrosis in tubular and myeloid cells but protects endothelial cells. Future strategies should either adopt cell-specific targeting or identify upstream master regulators of its context-dependent functions. Although the kidney is not a primary organ for energy metabolism, it plays a crucial role in lactate metabolism. Increased glycolysis in TECs or other factors that increase lactate levels can lead to histone lactylation, promoting renal fibrosis. In addition, reduced acetylation and increased ubiquitination in myoFbs can also induce their proliferation.

#### Lipid metabolism in renal fibrosis

FAO is critically important for the proliferative regeneration of renal TECs following injury and serves as a key energy source. Research using murine models has shown that FAO is transiently upregulated during the initial phase of ischemia, a response that is strongly associated with successful cellular repair and has been corroborated in human data. Conversely, the impairment of FAO is an established pathogenic hallmark of renal fibrosis. In patients with chronic kidney disease (CKD), impaired FAO causes energy depletion and suppresses the expression of key transcription factors that govern mitochondrial metabolism, whereas increased lipid uptake and synthesis lead to toxic lipid accumulation. This imbalance, which is worsened by hyperlipidemia and proteinuria, triggers oxidative stress, mitochondrial damage, and fibrosis [170]. Recent reviews have separately addressed lipid metabolism in podocytes and tubule cells in individuals with CKD [171, 172]. Here, we briefly summarize the roles of lipid-related enzymes in the development of renal fibrosis.

Under glomerular proteinuria conditions, the process of lipid uptake by TECs becomes considerably more intricate. Albumin, which serves as the main transporter of circulating lipids, is endocytosed by megalin when it is excessively filtered. Following internalization, albumin and its bound FAs dissociate. The liberated lipids subsequently enter TECs either through specific transporter-mediated mechanisms or diffusion [173]. While cardiac CD36 upregulation is closely related to lipotoxicity, its role in the kidney may differ; TEC-specific CD36 overexpression can increase lipid uptake but concurrently stimulate FAO without inducing overt renal damage [174]. In contrast, fatty acid transport protein 2 (FATP2) has emerged as a pivotal fatty acid transporter in proximal tubule cells. Its genetic deletion mitigated lipid accumulation and lipotoxicity in experimental models of renal fibrogenesis [175]. Astragaloside IV, a key bioactive component derived from *Astragalus membranaceus*, protects against diabetic kidney disease (DKD)-associated renal fibrosis by targeting FATP2. By blocking this fatty acid transporter, it reduces lipid accumulation in tubules, which mitigates lipotoxicity and produces antioxidant and anti-inflammatory benefits [176].

Gene expression analyses in human, murine, and single-cell studies have consistently demonstrated that kidney disease and fibrosis are associated with the systemic downregulation of FAO [170, 177]. Key FAO-related genes, including peroxisome proliferator-activated receptor α (PPARα), estrogen-related receptor α (ESRRα) and the peroxisome proliferator-activated receptor γ coactivator 1α (PGC-1α) are significantly compromised in injured kidneys, particularly within TECs. These findings are further corroborated by the elevated levels of acyl carnitines observed in patients, indicating a functional metabolic blockade of FAO [178]. Adipose triglyceride lipase (ATGL), a key lipid hydrolase, is downregulated in TECs during the transition from acute kidney injury (AKI) to CKD. Functionally, pharmacological inhibition of ATGL with atglistatin can exacerbate lipid accumulation and suppress FAO, ultimately aggravating renal fibrosis. Conversely, ATGL overexpression reduced lipid deposition by upregulating the activity of the PPARα/PGC-1α pathway both in vitro and in vivo [179]. Cannabinoid Receptor 2 (CB2), an important receptor of the endocannabinoid system, can initiate a signalling cascade through the β-arrestin 1/Src pathway, leading to β-catenin activation. This process subsequently suppresses the PPARα/PGC-1α transcriptional axis, resulting in impaired FAO in TECs and ultimately driving the progression of renal fibrosis [180]. Psoriasin, also called S100 calcium-binding protein A7 (S100A7), an epithelial-specific S100 protein, is highly upregulated in TECs from both CKD patients and mouse fibrotic models. Its levels are correlated with renal function decline and fibrosis progression. Mechanistically, S100A7 overexpression stabilizes β-catenin by preventing its ubiquitination and degradation, leading to the downstream suppression of PGC-1α. Crucially, the tubule-specific deletion of S100A7 can increase FAO, reduce lipid peroxidation and ameliorate renal fibrosis and dysfunction in mouse injury models, establishing S100A7 as a previously unrecognized driver of fibrosis [181]. In DKD, impaired FAO is also a key pathological feature. Natural compounds such as resveratrol, quercetin and berberine have been shown to restore FAO function by activating the PPARα signaling axis, upregulating downstream targets such as CPT1A, thereby reducing lipid accumulation, improving mitochondrial homeostasis and alleviating renal fibrosis [182]. Berberine also promotes FAO by activating the AMP-activated protein kinase (AMPK)/PGC-1α pathway and reducing lipid deposition in renal tubular epithelial cells [182]. The cystine-glutamate antiporter SLC7A11 and the glutathione-dependent peroxidase 4 (GPX4) play critical roles in maintaining cellular redox homeostasis and limiting lipid peroxidation, which is closely associated with renal fibrosis. Hederagenin (HDG) was found to inhibit SMAD3 phosphorylation, thereby reducing NADPH Oxidase 4 (NOX4) expression, restoring GPX4 and SLC7A11 levels, ultimately ameliorating lipid peroxidation and renal fibrosis in DKD [183]. Recently, a "TWA signaling network" (TGF‑β, Wnt and Ang II) has been proposed as a self‑amplifying loop driving progressive renal fibrosis, in which Klotho acts as an endogenous suppressor that physically interacts with TGF‑β receptor II and Wnt ligands to simultaneously block multiple pro‑fibrotic pathways [184]. This multi-target perspective complements the metabolic strategies discussed above, suggesting that combining metabolic modulation with signal pathway inhibition may offer a more effective approach to combat renal fibrosis.

In this section, we have summarized the alterations in glucose and lipid metabolism in various renal cell types during CKD, with a particular focus on tubular epithelial cells. The predominant metabolic shifts observed involve increased glycolysis, increased FA uptake and impaired FAO, collectively promoting lipotoxicity. Furthermore, intermediate metabolites serve as critical signals connecting glycolysis and lipid metabolic pathways. The predominant limitation is the inability of current animal models to replicate the chronic, multifactorial progression of human CKD, as they predominantly capture acute injury phases. We should pay more attention to human kidney organoids and multiomics approaches to identify the protective mechanisms through which renal TECs handle CD36-mediated lipid influx. Additionally, while amino acid metabolism also undergoes remodelling in CKD, this aspect has been systematically elucidated elsewhere and falls beyond the scope of the current discussion [185].

### Fibrosis in skin, peritoneum and glands

#### Metabolic reprogramming in skin fibrosis

Cutaneous fibrosis, as exemplified by systemic sclerosis (SSc), keloids and hypertrophic scars, is driven by a complex interplay of fibroblast activation, immune dysregulation and ECM remodeling, all of which are underpinned by profound alterations in cellular metabolism. While the shift from FAO to aerobic glycolysis has been widely observed as a metabolic feature of activated Fbs in various fibrotic diseases, an equally critical but less explored dimension involves the expression changes and PTMs of the enzymes that govern these metabolic pathways [186, 187].

In fibrotic skin, key glycolytic enzymes exhibit marked upregulation and altered activity. HK2, PFKFB3 and LDHA are consistently elevated in SSc and keloid Fbs, supporting the enhanced glycolytic flux required for sustained proliferation and collagen synthesis [187]. PKM2 is consistently upregulated in keloid Fbs. Beyond its canonical role in glycolysis, PKM2 undergoes nuclear translocation, where it functions as a transcriptional co-activator to promote cell proliferation and collagen gene expression. This subcellular redistribution is regulated by PTMs, notably phosphorylation at specific residues such as Ser37 and Tyr105, which dictate its tetramerization status and nuclear localization [188]. Similarly, in the context of lipid metabolism, the expression of CPT1A, the rate-limiting enzyme of FAO, is reduced in SSc Fbs, contributing to lipid accumulation and lipotoxicity. Conversely, the expression of PPARγ defines a metabolically distinct subset of Fbs localized in the deep dermis and perivascular regions, characterized by anti-inflammatory properties and a gene expression profile associated with lipid metabolism [189].

Importantly, PTMs also orchestrate the signaling pathways that drive fibrosis. The canonical TGF-β/SMAD pathway is modulated by phosphorylation of SMAD2/3, which controls their nuclear translocation and transcriptional activity, while acetylation of SMAD3 influences its stability and interaction with transcriptional co-activators [188]. JAK2 is activated by phosphorylation at Tyr1007/1008 in response to TGF-β stimulation in SSc Fbs, leading to subsequent activation of signal transducer and activator of STAT3. This JAK2-STAT3 axis promotes FMT and enhances collagen synthesis. Notably, STAT3 integrates signals from multiple profibrotic kinases, including SMAD3 and JNK, thereby linking cytokine signalling directly to metabolic reprogramming. Pharmacological inhibition of JAK2 or STAT3 attenuates fibroblast activation and experimental fibrosis, highlighting the therapeutic potential of targeting these PTM-regulated pathways [190]. Emerging evidence further highlights the role of lactate-derived histone lactylation in epigenetically reprogramming Fbs and immune cells to sustain chronic inflammation and fibrosis [191]. The accumulation of lactate, driven by enhanced glycolysis, directly fuels this modification, creating a positive feedback loop linking metabolic flux to epigenetic regulation.

In summary, the metabolic reprogramming of skin fibrosis is not solely defined by pathway-level shifts but is critically governed by changes in the expression and PTMs of metabolic enzymes, as well as downstream signaling proteins. The phosphorylation, acetylation, and lactylation of these key regulators integrate metabolic status with fibrotic gene expression, creating a self-sustaining cycle. Targeting these PTMs and the enzymes represents a promising strategy for developing more precise therapies for fibrotic skin diseases.

#### Metabolic changes in peritoneum and glands fibrosis

Building on the central role of metabolic reprogramming and PTMs in fibrotic diseases across multiple organs, emerging evidence highlights similar mechanisms in peritoneal and glandular fibrosis.

In peritoneal fibrosis associated with peritoneal dialysis (PD), continuous exposure to high-glucose-based dialysis solutions drives marked changes in the expression and PTM status of key metabolic enzymes. HK2 is consistently upregulated in peritoneal mesothelial cells (PMCs) and its activity is enhanced by AKT-mediated phosphorylation at Thr473, promoting glucose retention and glycolytic flux. In addition, PFKFB3 undergoes phosphorylation at Ser461 by AMPK, which stabilizes the enzyme and increases its product, F-2,6-BP, thereby allosterically activating glycolysis [192]. LDHA can be acetylated at Lys5, a modification that enhances its enzymatic activity and promotes lactate production. Elevated lactate subsequently drives histone H3K18 lactylation, which activates pro-fibrotic gene transcription [193].

Lipid metabolism is also profoundly altered. CPT1A exhibits reduced expression in fibrotic PMCs, accompanied by decreased acetylation at Lys residues that normally stabilize the enzyme. This hypoacetylation mediated by sirtuin 3 correlates with impaired FAO, lipid accumulation and lipotoxicity [192]. Conversely, pharmacological activation of CPT1A restores FAO and attenuates fibrosis, underscoring the therapeutic potential of targeting this PTM-regulated enzyme.

In the pancreas, activated pancreatic stellate cells (PSCs) exhibit marked alterations in the PTM status of metabolic enzymes. PKM2 is upregulated and undergoes phosphorylation at Tyr105 by fibroblast growth factor receptor (FGFR) signaling, which promotes its tetramer-to-dimer transition and subsequent nuclear translocation. Nuclear PKM2 functions as a transcriptional co-activator, binding to HIF-1α and STAT3 to induce the expression of glycolytic enzymes and pro-fibrotic genes [194]. GLS is also elevated in activated PSCs, with its activity regulated by acetylation at Lys311, a modification that enhances both stability and catalytic efficiency. Inhibition of GLS or blockade of its acetylation reduces collagen synthesis and attenuates fibrosis, linking amino acid metabolism directly to ECM production [194].

Across peritoneal and pancreatic fibrosis, the PTM status of metabolic enzymes critically determines enzyme activity, subcellular localization and downstream fibrotic outcomes. Key modifications include phosphorylation of HK2 at Thr473, PFKFB3 at Ser461 and PKM2 at Tyr105, as well as acetylation of LDHA at Lys5, CPT1A at Lys residues and GLS at Lys311. Targeting these specific PTM nodes represents a promising precision strategy for mitigating fibrosis in these tissues.

### Mechanistic insights across organs and metabolic crosstalk

#### Shared mechanisms and distinct features

Upon injury, the body activates complex biological programs that try to confine the affected area and ultimately lead to the recovery of physiological function. The epithelium functions as a protective layer that is composed of highly polarized cells and is tightly interconnected via intercellular junctions. Consequently, the epithelium constitutes the most common site where initial injury occurs. Regardless of the degree of injury, these stressed epithelial cells release key fibrotic mediators such as TGF-β. These cytokines activate local Fbs and immune cells, initiating the formation of fibrotic tissue defined by ECM deposition. While the ECM normally stabilizes tissue and provides a scaffold for cell migration during healing, its dysregulated turnover in pathological fibrosis leads to persistent accumulation of components such as collagen. The progressive accumulation of ECM disrupts tissue architecture, ultimately causing organ dysfunction [195].

While fibrosis in various organs shares common signalling pathways, such as those involving TGF-β and FGF, targeting TGF-β has thus far shown clinically meaningful efficacy largely in pulmonary fibrosis [196]. Here, we summarize key metabolic alterations during the progression of fibrotic diseases, including increased glycolysis, upregulated glutamine metabolism, and impaired FAO. Furthermore, fibrotic processes in different organs, such as the heart, kidneys and liver, are closely interrelated. Clinical and autopsy studies have consistently demonstrated a significant association between CKD and myocardial fibrosis, independent of macroscopic left ventricular hypertrophy [197]. In the context of CKD, the nutrient-sensing pathways mTOR and AMPK play opposing roles in cardiac remodeling: mTOR activation promotes cardiac fibrosis, whereas AMPK activation supports mitochondrial function and substrate oxidation, resulting in cardioprotection. Targeting these metabolic pathways holds therapeutic potential. Certain drugs, such as SGLT2i and glucagon-like peptide-1 receptor (GLP-1R) agonists, exert cardioprotective effects on HF and may benefit uremic cardiomyopathy, although their mechanisms in CKD remain underexplored. These agents might act through systemic metabolic improvements, such as weight loss, glycemic control, and blood pressure reduction, as well as direct effects on cardiac inflammation and energy metabolism [198]. MASLD is becoming increasingly prevalent and represents the leading cause of end-stage liver disease. A high prevalence of MASLD is observed in patients with HF, yet the synergistic effects of combined metabolic and congestive stress on liver disease progression remains unclear [199]. Liver fibrosis is a strong predictor of adverse hepatic and cardiovascular outcomes [200]. The frequent co-occurrence of MASLD, metabolic syndrome and cardiovascular diseases reflect shared pathophysiological mechanisms centered on metabolic dysfunction. These processes promote ectopic fat deposition in the liver and epicardium, driving endothelial dysfunction, systemic inflammation, and subsequent complications, including atherogenic dyslipidemia, diastolic dysfunction and fibrosis [201]. Given the pathophysiological overlap between MASLD and cardiometabolic diseases, dual-targeting agents (for instance, GLP-1R agonists, PPAR agonists and SGLT2i) represent a rational therapeutic strategy. These drugs produce multisystem benefits, improving cardiovascular risk factors and directly ameliorating hepatic steatosis and fibrosis [202].

Fibrosis is a common pathological outcome in multiple organs, yet its development and functional impact vary significantly by tissue context. Cardiac fibrosis, for example, arises independently of epithelial injury and primarily presents as interstitial or replacement scarring. This process compromises ventricular compliance and disrupts electrical conduction—functional consequences distinct from those in other organs. In contrast, pulmonary, hepatic, and renal fibrosis frequently originate from epithelial damage, engaging mechanisms such as the epithelial–mesenchymal transition and activation of organ-specific stromal cells like HSCs or renal TECs. These differences in cellular origin and functional sequelae highlight the organ-specific nature of fibrotic diseases [203].

#### Crosstalk between metabolism and immune system in fibrosis

Fibrosis represents a common final pathway leading from chronic organ injury to failure. Long viewed as a fibroblast-driven repair disorder, fibrosis is now recognized as a dynamic, bidirectional crosstalk with the immune system. While the immune-fibroblast interface has been extensively reviewed elsewhere [116, 148, 204, 205], this section briefly highlights several recent studies that exemplify key mechanistic advances.

Immune cells, particularly macrophages, act as central hubs. In the liver, recruited monocyte-derived macrophages (MoMFs) and scar-associated macrophages (SAMs) secrete profibrotic cytokines such as TGF-β, directly activating HSCs [116]. Crucially, macrophage function is highly plastic. The mechanosensitive ion channel Piezo1, for instance, enhances macrophage efferocytosis in response to increasing liver stiffness, thereby promoting fibrosis resolution [206]. Similarly, in the heart, C–C chemokine receptor type 2 (CCR2^+^) macrophages drive the differentiation of the pathogenic matrifibrocytes expressing fibroblast activator protein (FAP) and periostin (POSTN) via IL-1β signaling, exacerbating post-infarct remodeling [207]. In the lung, the solute carrier SLC15A3 regulates macrophage ROS via the p62-nuclear factor erythroid 2-related factor 2 (NRF2) axis, thus driving fibrotic progression [208]. These findings underscore the macrophage's role as a key rheostat, integrating mechanical, metabolic and inflammatory cues.

Fbs, in turn, are not passive targets. Activated HSCs secrete chemokines including C–C motif chemokine ligand 2 (CCL2) and CCL5 that recruit monocytes and T cells, perpetuating a pro-fibrotic feed-forward loop [116]. Single-cell profiling has further refined this view, revealing that CFs are heterogeneous. Specifically, an FAP^+^/POSTN^+^ matrifibrocyte lineage, distinct from alpha-smooth muscle actin-positive (ACTA2^+^) myoFbs, is selectively regulated by macrophage-derived IL-1β [207]. This discovery highlights how targeting precise immune–fibroblast communication pathways, such as the IL-1β/IL-1R axis, may yield effective anti-fibrotic therapies.

Finally, metabolic reprogramming provides a critical link between immunity and fibrosis. In the kidney, impaired FAO in proximal TECs leads to lipotoxicity and mitochondrial damage, releasing damage-associated molecular patterns (DAMPs) that activate macrophages, while activated myoFbs shift to aerobic glycolysis to support their synthetic functions [148]. In pulmonary fibrosis, macrophage metabolism dictates function: pro-inflammatory M1 cells rely on glycolysis, while pro-resolving M2 cells utilize FAO, and metabolites like itaconate can actively suppress fibrosis [205]. In metabolic dysfunction-associated steatohepatitis (MASH), accumulation of toxic lipid intermediates such as free cholesterol and ceramides within hepatocytes triggers NLRP3 inflammasome activation, driving both pyroptosis and paracrine activation of KCs and HSCs [12]. Thus, metabolites are not merely energy substrates but serve as critical signaling molecules that orchestrate intercellular communication in the fibrotic niche.

In conclusion, across the organs including heart, lungs, liver, and kidneys, fibrosis is driven by a conserved immunologic and metabolic logic. This axis is defined by specific immune subsets (such as CCR2 ^+^ macrophages), fibroblast lineages (like FAP ^+ ^matrifibrocytes), core signaling pathways (such as IL-1β, TGF-β), and metabolic switches (like glycolysis and FAO). A deeper understanding of this integrated network is paving the way for precision therapies including chimeric antigen receptor-macrophage (CAR-M) cells targeting FAP-positive fibroblasts cells [209], Piezo1 agonists and SGLT2 inhibitors, aiming at reversing organ fibrosis.

### Mitochondrial dysfunction: the core driver of metabolic reprogramming in fibrosis

Emerging evidence across multiple organs, including the kidney, liver, lung, and heart, positions mitochondrial dysfunction not as a mere consequence but as a central driver of the metabolic reprogramming that fuels fibrogenesis. While metabolic reprogramming, such as the shift from FAO to glycolysis, is a well-recognized feature of activated myoFbs, an accumulating body of studies have indicated that these metabolic changes are fundamentally governed by the functional and structural integrity of mitochondria. Thus, mitochondrial dysfunction is increasingly understood as the upstream hub that orchestrates the profibrotic cascade [204, 210].

A key mechanism linking mitochondrial failure to fibrosis is the disruption of mitochondrial quality control (MQC) systems. In healthy cells, a dynamic balance of mitophagy, biogenesis, and fusion/fission maintains mitochondrial homeostasis. In fibrotic diseases across organs, this balance is profoundly disturbed. The failure of PTEN-induced putative kinase 1 (PINK1)/Parkin-mediated mitophagy, for instance, leads to the accumulation of dysfunctional, ROS-overproducing mitochondria in alveolar epithelial cells in pulmonary fibrosis and in tubular cells in CKD [211, 212]. This accumulation not only creates a bioenergetic deficit but also transforms mitochondria into signaling hubs that perpetuate injury. Similarly, an imbalance in mitochondrial dynamics, characterized by excessive fission via dynamin-related protein 1 (DRP1) or impaired fusion via mitofusin 2 (MFN2)/optic atrophy 1 (OPA1), has been directly implicated in fibroblast activation and epithelial cell apoptosis across hepatic, cardiac, and renal fibrosis models [30, 210, 211].

Beyond MQC failure, damaged mitochondria act as potent instigators of inflammation and fibrogenesis through the release of mitochondrial DAMPs. When mitochondrial DNA (mtDNA) is released from mitochondria into the cytosol, it initiates the cyclic GMP-AMP synthase (cGAS)—stimulator of interferon genes (STING) signaling cascade, a key driver of sterile inflammation that promotes HSCs activation and renal interstitial fibrosis [204, 213]. Concurrently, excessive generation of mitochondrial ROS (mtROS) from a compromised electron transport chain serves as a critical signaling node, activating pro-fibrotic pathways such as TGF-β and triggering the NLRP3 inflammasome across multiple organ systems [214, 215]. This oxidative burden further amplifies mitochondrial damage, creating a self-reinforcing cycle of dysfunction and fibrosis.

Importantly, mitochondrial dysfunction is also intimately linked to cellular senescence, a hallmark of aging that drives chronic lung diseases such as IPF. Accumulation of senescent cells, characterized by dysfunctional mitochondria and a pro-inflammatory secretory phenotype (SASP), perpetuates tissue remodeling and impairs regenerative capacity [211]. In the heart, impaired mitophagy and excessive mitochondrial fission similarly promote fibroblast activation and pathological remodeling under pressure overload or ischemic stress [30].

Collectively, these findings establish a new paradigm: mitochondrial dysfunction is not a secondary phenomenon but a core pathogenic mechanism across fibrotic diseases. While shifts in metabolic pathways such as glycolysis are critical for supporting the biosynthetic demands of activated myoFbs, they are intrinsically linked to and driven by underlying mitochondrial failure. The intricate interplay between mtROS production, mtDNA release, and MQC collapse creates a self-sustaining cycle that drives myofibroblast activation and extracellular matrix accumulation. Therefore, future therapeutic strategies may benefit from moving beyond merely targeting downstream metabolic pathways toward approaches aimed at restoring mitochondrial homeostasis, such as mitophagy inducers, mitochondrial-targeted antioxidants, and agents that rescue electron transport chain function [214, 216].

The preceding discussion has highlighted that fibrogenesis across multiple organs, including heart, liver, lung, kidney, skin, peritoneum and glands, involves a complex metabolic network in which enhanced glycolysis is closely intertwined with fatty acid and glutamine metabolism. However, the expression and activity of individual metabolic enzymes, as well as the accumulation of specific metabolites, can vary considerably depending on the organ, cell type, and disease stage. To provide a clear and concise overview of the common findings that transcend these differences, we have summarized the most consistently reported metabolic players in two tables before discussing their therapeutic targeting below. We outline the key enzymes implicated in fibrosis-related metabolic reprogramming, including their common expression or activity changes and the core pathogenic mechanisms they mediate (Table 1). In addition, we compile the critical metabolites that accumulate or become metabolically rerouted during fibrogenesis, along with their downstream signaling or epigenetic effects (Table 2).

**Table 1:** Key enzymes involved in metabolic reprogramming across multiple organ fibrosis

No	Metabolic pathway	Enzyme	Common change in fibrosis	Mechanism	References
1	Glycolysis	HK2	Upregulated	Promotes glycolytic flux and generation of ROS;	[16, 33]
2	Glycolysis	PFKFB3	Upregulated	Promotes glycolytic flux and histone lactylation;	[37, 149]
3	Glycolysis	PKM2	Variable in cell type	Dimerization and nuclear translocation promote fibrogenic gene expression;	[42, 123]
4	Glycolysis	LDHA	Upregulated	Converts pyruvate to lactate; Interacts with HIF-1α to drive glycolytic gene expression;	[56, 130]
5	FA uptake	CD36	Variable (PTMs and membrane translocation)	Mediates lipid uptake and lipotoxicity; interacts with TLR4 to drive inflammation and fibrosis;	[66, 71]
6	Glutaminolysis	GLS1	Upregulated	Provides proline for collagen synthesis; drives HSC activation and ECM deposition;	[111, 143]
7	Lipogenesis	ACC	Upregulated	Converts acetyl CoA to malonyl CoA; drives HSC activation and fibrogenesis;	[134]

*PFKFB3* fructose-2,6-bisphosphatase, *ACC* acetyl-coenzyme A carboxylase, *TLR4* Toll-like receptor 4, *GLS1* glutaminase 1

**Table 2:** Key metabolites driving fibrogenesis across multiple organ fibrosis

No	Metabolite	Metabolic origin/fate	Change in fibrosis	Mechanism	References
1	Lactate	Glycolysis	Elevated in most fibrotic tissues; decreased in failing CMs	Stimulates TGF-β induced EndMT; drives H3K14la mediated KLF5 expression;	[63, 166]
2	Ceramides	Sphingolipid synthesis	Elevated	Promotes inflammatory macrophage polarization; induces mitochondrial dysfunction	[104, 138]
3	Proline	Glutaminolysis	Elevated	Essential substrate for collagen; glutamine-derived proline drives ECM deposition	[110]
4	Alanine	Glycolysis and glutaminolysis	Elevated	GPT2 bypasses GLS1 blockade to sustain collagen via alanine/glutamate regeneration;	[111]

*EndMT* endothelial-to-mesenchymal transition, *KLF5* Kruppel-like factor 5, *GPT2* glutamic-pyruvic transaminase 2

## Therapies targeting metabolic reprogramming in fibrosis

Building on the mechanistic insights into metabolic reprogramming in fibrosis, we turn to therapeutic strategies targeting these pathways. U.S. Food and Drug Administration (FDA)-approved antifibrotic drugs, such as pirfenidone, which targets TGF-β, and nintedanib, which targets receptor tyrosine kinase, have been extensively studied [28]. Both face challenges, including high liver toxicity, the need for high dosages, gastrointestinal side effects and the risk of photoallergic reactions. Therefore, their long-term tolerability requires further investigation. Given the diversity and complexity of metabolic regulation in fibrosis, targeting metabolic pathways such as glycolysis, lipid and glutamine metabolism represents a promising therapeutic direction. Although many studies remain at preclinical stages, these metabolism-centric strategies offer potential opportunities for treating fibrosis-related diseases.

### Treatments targeting glycolysis in fibrosis

MCT inhibitors, including phloretin, lonidamine, simvastatin, quercetin, and rotenone, have shown efficacy in preclinical models of various cancer types, such as breast, colorectal, cervical, and prostate cancers [217]. Similarly, competitive inhibitors of LDH (e.g., oxalate), selective LDHA inhibitors (e.g., gossypol), and other compounds, including phthalimides and dibenzofuran derivatives, have shown the potential to suppress tumor growth by targeting glycolytic pathways [218]. Notably, stiripentol, a clinically approved antiepileptic drug, has emerged as a promising LDHA inhibitor with significant clinical applications in oncology [219]. Considering their metabolic characteristics analogous to those of tumors, interventions targeting glycolytic enzymes, including small-molecule compounds, proteolysis-targeting chimaera (PROTAC) strategies for ubiquitination and proteasomal degradation, and nanomedicines designed to engineer enzymes or modulate lactate levels, have been developed [220, 221]. In addition, inhibiting PKM2 aggregation represents a promising therapeutic approach. Recent studies have identified compounds, such as K35 and its gueanalog K27, that dissolve PKM2 aggregates and alleviate age-associated fibrosis [222]. Due to the pivotal role of PKM2 in fibrosis, researchers have explored both activators and inhibitors of this enzyme. Nonetheless, despite promising research, no PKM2 modulator has been approved by the FDA [223]. PFKFB3, another key enzyme involved in glycolysis, can be suppressed by PFK158, which is the first inhibitor to be evaluated in a phase I clinical trial (NCT02044861) [224]. This trial plans to enroll 56 individuals and will employ a “3 + 3” cohort-based dose escalation design to evaluate PFK-158. The primary objectives are to characterize dose-limiting toxicity and establish either the maximum tolerated dose or a biologically effective dose. Given the extensive preclinical studies or clinical trials targeting glycolysis that are currently underway in oncology, significant potential exists to apply similar strategies in fibrosis-related clinical research.

During the progression of fibrosis, interorgan interactions among the aforementioned organs appear to occur. As discussed in the previous section, SGLT2i can suppress the formation of HIF-1α-induced PKM2 dimers, thereby contributing to balancing energy metabolism. Ertugliflozin, another SGLT2i, was shown to reduce the risk of hospitalization for HF in VERTIS CV. (Evaluation of Ertugliflozin Efficacy and Safety Cardiovascular Outcomes Trial, NCT01986881). In this multicenter double-blind trial, a total of 8246 patients with atherosclerotic cardiovascular diseases and type 2 diabetes were randomly assigned to receive once-daily ertugliflozin (5 mg or 15 mg) or placebo. Over a mean follow-up of 3.5 years, the study demonstrated that ertugliflozin was not inferior to the placebo in terms of the risk of major adverse cardiovascular events [225]. In addition, a higher hepatic steatosis index and fibrosis-4 index were associated with cardiovascular events in the VERTIS CV study [226]. Several clinical studies are in the patient recruitment phase, with no definitive conclusions yet available. Ertugliflozin will also be investigated for its effects on cardiac fibrosis in a prospective, randomized, double-blind, placebo-controlled clinical trial involving patients with NICM. This single-center trial is expected to recruit 52 participants, and cardiac magnetic resonance (CMR) is utilized to evaluate the degree of fibrosis (NCT04490681). Metformin, an antidiabetic drug and HK2 inhibitor that mimics G6P binding, is being assessed in a prospective phase 4 study for its efficacy and safety in preventing, stabilizing, or reversing cardiac fibrosis in individuals with a genetic deficiency in plasminogen activator inhibitor-1 (PAI-1) (NCT05317806). This study aims to enroll 15 patients who will receive metformin for a minimum of 36 months. TGF-β1 levels will be measured and cardiac imaging will be performed throughout the study. Furthermore, a comparative study including 64 participants is underway to evaluate the efficacy of metformin versus the SGLT2i henagliflozin in mitigating cardiac fibrosis. Participants will receive either 10 mg of henagliflozin once daily or 1000 mg of metformin twice daily, with the treatment lasting for 24 weeks. CMR will be employed to evaluate both cardiac function and the extent of fibrosis (NCT06059287). In addition, researchers have reanalyzed extant clinical trials and confirmed the association between glycolytic pathways and fibrosis. Data from the IMPROVE-T2D (NCT03620773) and RENAL-HEIR (NCT03584217) observational studies were integrated. Among 16 patients with youth-onset type 2 diabetes, 10 patients were treated with either canagliflozin or empagliflozin, while 6 patients received standard therapy. Renal biopsy tissue samples were ultimately utilized to evaluate alterations in renal energy metabolism. These data indicate that SGLT2i treatment alleviates the increased glycolysis observed in proximal TECs, which is mediated by the inhibition of the mTORC1 pathway [227]. Exenatide, a GLP-1R agonist, was reported to decrease the 24-h urinary albumin excretion rate (24 h-UAER) in diabetic nephropathy patients in a phase 4 multicenter randomized trial (NCT02690883) [228]. This study evaluated exenatide plus insulin glargine versus insulin lispro/glargine in 92 patients with macroalbuminuric T2DM. After 24 weeks of treatment, the exenatide group exhibited a significantly greater reduction in the urinary albumin excretion rate, which was accompanied by weight loss and a lower incidence of hypoglycemia, although the incidence of gastrointestinal adverse events increased. These results validate the renoprotective effects of exenatide, which are mediated by both a decrease in albuminuria and systemic metabolic advantages, in individuals with diabetic nephropathy. Omipalisib, an inhibitor of the PI3K/mTOR pathway, has been evaluated in subjects with IPF (NCT01725139). In this trial, 17 patients were recruited and received omipalisib for approximately 8 days (7 to 10 days). Glucose uptake, as measured by ^18^F-2-fluoro-2-deoxy-d-glucose (FDG) positron emission tomography/computed tomography (PET/CT) scans, was significantly reduced in the fibrotic areas of lung tissues [229].

In this section, we summarized preclinical and clinical studies related to glycolysis and fibrosis. Clinical studies related to glycolysis are currently limited in number, with relatively small sample sizes. Additionally, investigations of glycolytic enzymes and their PTMs are predominantly confined to the preclinical phase. As more preclinical findings are published, we anticipate an increase in clinical research focused on glycolytic enzymes and their role in fibrosis.

### Treatments orchestrating lipid metabolism in fibrosis

As a partial inhibitor of stearoyl-CoA desaturase (SCD1), aramchol has been shown to ameliorate steatohepatitis and fibrosis in rodents and reduce steatosis in early-phase trials [230]. Building on this premise, the phase 2b ARREST trial (NCT02279524) evaluated two doses (400/600 mg) versus the placebo in 247 non-alcoholic steatohepatitis (NASH) patients. While patients in the 600 mg arm failed to achieve a statistically significant result for the primary endpoint of hepatic fat reduction, they showed clinically relevant histological improvements: NASH resolution without fibrosis progression occurred in 16.7% of patients compared with 5% in the placebo group, accompanied by a significant reduction in alanine aminotransferase (ALT) levels. The favorable safety profile observed supports continued investigation in an ongoing phase 3 trial [231]. NCT03449446, a Phase 2, randomized, double-blind, placebo-controlled trial, is designed to evaluate the safety and efficacy of selonsertib (an apoptosis signal-regulating kinase 1 inhibitor), firsocostat (an ACC inhibitor), cilofexor (an Farnesoid X receptor agonist), and their combinations in 392 patients with bridging fibrosis (F3) or compensated cirrhosis (F4) due to NASH. The cilofexor/firsocostat combination showed promising antifibrotic potential, with significant improvements in multiple histological parameters. This dual therapy also significantly enhanced liver biochemical and noninvasive markers while showing acceptable tolerability, supporting its potential for managing advanced NASH fibrosis with extended treatment [232]. TVB-2640, a FASN inhibitor, was evaluated in the randomized 2a FASCINATE-1 trial (NCT03938246) involving 99 NASH patients with at least 8% liver fat and confirmed fibrosis. After 12 weeks of treatment, TVB-2640 demonstrated significant, dose-dependent efficacy in reducing liver fat levels, along with improvements in multiple metabolic, inflammatory, and fibrotic biomarkers, all with a favorable safety profile. These results support its therapeutic potential for treating NASH by targeting de novo lipogenesis and addressing multiple disease pathways [233]. Fenofibrate, an agonist of PPARα, exerts renoprotective effects on type 2 diabetic kidney disease by reducing albuminuria and slowing the decrease in the estimated glomerular filtration rate (eGFR) over 5 years [234]. Based on this evidence, a phase 2 clinical trial is being developed specifically for patients with type 1 diabetic (T1D) kidney disease. This pilot study will enroll 40 T1D patients with early-to-moderate DKD to assess the efficacy of fenofibrate in protecting the kidney and collect critical data for the design of future trials, addressing a significant evidence gap in T1D nephropathy management (NCT04929379). Preclinical studies have demonstrated that FASN inhibitors such as triclosan and EGCG ameliorate renal fibrosis in a mouse ischemia–reperfusion injury model, resulting in improved functional and histological outcomes [148]. However, the therapeutic potential of these drugs remains unexplored in clinical settings, highlighting a significant translational gap in kidney disease treatment.

### Treatments targeting glutamine metabolism in fibrosis

To date, no clinical trials have been registered for GLS1 inhibitors or other direct glutamine metabolism-targeting agents specifically for fibrotic diseases. Although direct clinical evidence is lacking, there are promising preclinical studies and related clinical investigations that inform this area.

In pulmonary fibrosis, glutaminolysis is required for myofibroblast differentiation and collagen synthesis. The GLS1 inhibitor CB-839 suppressed TGF-β1-induced collagen I and III expression in human lung Fbs and attenuated bleomycin-induced lung fibrosis in mice [110]. The SLC1A5 (glutamine transporter) inhibitor V-9302 reduced glutamine uptake and collagen synthesis, and ameliorated bleomycin-induced pulmonary fibrosis [110]. Tanshinone IIA inhibited GLS1 expression, reduced glutamine metabolic flux into the TCA cycle, and suppressed proline biosynthesis, thereby decreasing collagen production [235]. The prolyl-tRNA synthetase 1 (PARS1) inhibitor DWN12088, which targets proline synthesis (a glutamine-derived pathway critical for collagen production), has completed three phase 1 trials (NCT04767815, NCT04888715, NCT04888728) and is currently in phase 2 (NCT05389215) development for IPF. NCT04767815 evaluated the pharmacokinetics and safety of DWN12088 in healthy volunteers (completed in 2021). NCT04888715 assessed drug-drug interactions between DWN12088 and pirfenidone or nintedanib in 48 healthy volunteers. NCT04888728 evaluated drug-drug interactions between DWN12088 and nebivolol or paroxetine in 24 healthy male volunteers. The ongoing phase 2 trial is a randomized, double-blind, placebo-controlled, multicenter study enrolling approximately 102 IPF patients in the United States and Korea, stratified by background antifibrotic use (pirfenidone, nintedanib or none). The primary endpoint is absolute change in forced vital capacity (FVC) in milliliters from baseline to week 24; secondary endpoints include percent-predicted FVC, diffusing capacity of the lungs for carbon monoxide (DLCO), 6-min walk test (6MWT), patient-reported outcomes (PROs) and safety [110, 236].

In liver fibrosis, although no GLS1 inhibitor trial has been registered, a related clinical trial targeting glutamine metabolism via ammonia detoxification has been conducted. A double-blind randomized placebo-controlled trial (phase not specified, CTRI/2019/05/018966) was performed in 140 cirrhotic patients with overt hepatic encephalopathy (HE) grade III-IV. Patients received intravenous L-ornithine L-aspartate (LOLA, 30 g/24 h for 5 days) or placebo, plus lactulose and rifaximin. The primary outcome was improvement in HE grade at day 5. Results showed higher improvement rates, shorter recovery time and lower 28-day mortality in the LOLA group. Although this trial targeted HE rather than fibrosis directly, it demonstrates that pharmacological modulation of glutamine metabolism is clinically feasible [237].

In conclusion, despite strong preclinical evidence, no GLS1 inhibitor trials have been registered for fibrotic diseases. However, the ongoing phase 2 trial of DWN12088 in IPF and the positive LOLA trial in cirrhosis suggest that interventions targeting glutamine metabolism and its downstream pathways are clinically viable and warrant further investigation. We have summarized the clinical trials and targets on metabolic reprogramming in fibrosis-related diseases described above (Table 3).

**Table 3:** Clinical trials target metabolic reprogramming in fibrosis

No	Drug	Target	Organ	Phase	ClinicalTrials.gov Identifier	References
1	PFK158	PFKFB3	-	1	NCT02044861	[224]
2	Ertugliflozin	PKM2	Heart	3	NCT01986881, NCT04490681	[225]
3	Metformin	HK2	Heart	4	NCT05317806	ClinicalTrials.gov
4	Metformin and henagliflozin	-	Heart	Not Applicable	NCT06059287	ClinicalTrials.gov
5	SGLT2 inhibitors	mTORC1	Kidney	2	NCT03620773, NCT03584217	[227]
6	Exenatide	GLP-1R	Kidney	4	NCT02690883	[228]
7	Omipalisib	PI3K/mTOR	Lung	1	NCT01725139	[229]
8	Aramchol	SCD1	Liver	2b	NCT02279524	[231]
9	Cilofexor and Firsocostat	ACC	Liver	2	NCT03449446	[232]
10	TVB-2640	FASN	Liver	2a	NCT03938246	[233]
11	Fenofibrate	PPARα	Kidney	2	NCT04929379	[234]
12	DWN12088	PARS1	Lung	1,2	NCT04767815, NCT04888715, NCT04888728, NCT05389215	[236]

*PI3K* phosphatidylinositol 3-kinase, *mTORC1* mammalian target of rapamycin complex 1, *SCD1* stearoyl-CoA desaturase 1, *GLP-1R* glucagon-like peptide-1 receptor, *PARS1* prolyl-tRNA synthetase 1

Manipulating energy metabolism has emerged as a promising therapeutic strategy for fibrosis. However, its translation into clinical practice faces considerable challenges, spanning both biological mechanisms and practical applications. These obstacles can be broadly classified into the general limitations of antifibrotic drug development and specific issues related to metabolic targeting.

While numerous antifibrotic agents have shown efficacy in preclinical models, their performance in human trials has often been disappointing. This translational gap may stem from several factors: animal models often fail to fully mirror the clinical features observed in human diseases; the compressed time course of disease progression in animals frequently fails to capture the prolonged trajectory characteristic of human chronic illnesses; variability in drug pharmacokinetics is based on differences in the reactions to pharmacological suppression, genetic knockdown and small interfering RNAs; and shortcomings in clinical trial design, including heterogeneous responses among participants coupled with a modest overall treatment benefit. Another central challenge lies in achieving cell type-specific inhibition of energy production, effectively suppressing fibrotic cells without impairing the global metabolic function of the organ. Concurrently, therapeutic strategies must be devised to discriminate between activated fibrogenic cells and their normal counterparts. Critical questions regarding treatment timing and safety remain open. Which phase of fibrosis is most susceptible to metabolic intervention and what strategies could minimize collateral damage to nontargeted cells are unclear.

## Metabolites: from biomarkers to therapeutic targets

Beyond their role in therapy, metabolic reprogramming also hold promise for fibrosis diagnosis and biomarker discovery. Metabolites derived from dysregulated glycolysis, lipid metabolism and glutamine metabolism can serve as non-invasive diagnostic indicators in fibrotic diseases. In addition, emerging technologies for metabolite identification are opening new avenues for targeted therapy.

### Metabolites as diagnostic biomarkers

Metabolic abnormalities are well‑established hallmarks of disease and the identification of small-molecule metabolite signatures has emerged as a powerful strategy for early diagnosis, disease classification, and risk stratification. In contrast to genomics or proteomics, the metabolome provides a dynamic and functional profile of the interplay between genetic predisposition, environmental exposures and pathophysiological processes, thereby offering a more direct reflection of the phenotypic state [238]. Consequently, metabolites in easily accessible biofluids such as serum, plasma, and urine have been extensively investigated as non-invasive biomarkers for a range of fibrotic and metabolic diseases.

In HF, particularly heart failure with preserved ejection fraction (HFpEF), metabolomic profiling has identified myo-inositol as a metabolite consistently elevated in patients [239]. Myo-inositol levels were strongly correlated with renal function, but HFpEF status remained an independent predictor of elevated myo-inositol after adjusting for kidney function and comorbidities. Importantly, elevated myo-inositol levels (≥ 69.8 μM) were associated with poor clinical outcomes, including hospitalization for HF or death. Myo-inositol also correlated positively with N-terminal pro-B-type natriuretic peptide (NT-proBNP), troponin, FGF-23 and extracellular volume, suggesting its involvement in adverse myocardial remodeling and fibrosis. These findings establish myo-inositol as a potential diagnostic and prognostic tool for HFpEF, with its transporter sodium/myo-inositol cotransporter 1 (SMIT1) emerging as a promising therapeutic target.

Similarly, in IPF, lipidomic and metabolomic studies have identified circulating metabolites that correlate with disease presence and severity. Serum levels of non‑esterified fatty acids (NEFAs), long‑chain acylcarnitines, and specific ceramides are significantly higher in patients relative to age‑matched controls, indicating enhanced lipid mobilization and mitochondrial dysfunction. Diminished levels of branched‑chain amino acids (BCAAs), including leucine, isoleucine and valine were associated with milder physiological impairment, as measured by higher DLCO [104]. A recent analytical workflow, Lipidepifind, has been developed to identify structurally modified “epi‑metabolites” in IPF serum. This approach uncovered two oxidative cleavage reactions and identified phosphatidylcholine (16:0/9:0 (CHO)) and (16:0/5:0 (COOH)) as promising diagnostic biomarkers, demonstrating superior performance over conventional database-dependent methods [240]. Other metabolomic studies in IPF have identified palmitoyl ethanolamide (PEA) and 2-amino-1,3,4-octadecanetriol as potential diagnostic and prognostic biomarkers, with both metabolites showing significant positive correlations with inflammatory markers and coagulation abnormalities [241].

In MASLD, serum and plasma metabolomics have proven invaluable for distinguishing simple steatosis from progressive MASH and significant fibrosis. Plasma succinate has been shown to accumulate early in HFD-induced male mice, correlating with the progression of MASLD and MASH, and proposed it as a potential early indicator [242]. Two serum metabolite panels have been developed and validated for the non-invasive diagnosis of at-risk MASH. The fibrosis panel comprised ten metabolites including guanidinoacetic acid (GAA), chenodeoxycholic acid (CDCA) and α-linolenic acid. An inflammation panel similarly identified patients with mild to severe inflammatory activity. Notably, GAA and sebacic acid (SA), which were depleted during disease progression, demonstrated therapeutic efficacy in preclinical MASH models, functionally validating their association with the disease [243]. Lipoprotein metabolites, particularly very low-density lipoprotein (VLDL) and low-density lipoprotein (LDL), have been identified as key biomarkers of fibrosis progression in diet-induced MASH mouse models and later validated in a retrospective cohort of patients with cirrhosis [244].

In CKD, urine serves as an ideal non-invasive source for biomarker discovery, providing direct insights into kidney pathophysiology. Several urinary protein biomarkers have been validated for early detection and prognostication. The CKD273 classifier, a 273-peptide panel detected by capillary electrophoresis-mass spectrometry, can distinguish DKD patients at high or low risk of progression, with an area under the receiver operating characteristic curve (AUROC) of 0.87 in prospective trials [245]. Among urinary protein biomarkers, decreased epidermal growth factor (EGF) correlates with tubular atrophy and fibrosis, whereas kidney injury molecule-1 (KIM-1) and CCL2 reflect tubular damage and immune cell infiltration, respectively. Combinations of these markers improve risk prediction beyond traditional clinical parameters [245]. Metabolomic studies have also identified D-serine as a promising marker of kidney function decline, with plasma levels accurately reflecting measured eGFR and predicting CKD progression [246].

In untreated SSc patients, serum metabolomic profiling has revealed a distinct signature characterized by elevated glycolytic metabolites, altered amino acid and lipid metabolism. Several metabolites, including all-trans-retinoic acid and betaine, are decreased at baseline but partially restored following immunosuppressive therapy, suggesting their potential as markers of treatment response. Notably, all-trans-retinoic acid correlates negatively, whereas D-glucuronic acid correlates positively, with the modified Rodnan skin score (mRSS), a clinical index of skin fibrosis severity. Machine learning has further identified a metabolite panel that predicts skin fibrosis progression, underscoring the utility of metabolomics for patient stratification [238].

Collectively, these findings underscore that metabolite-based biomarkers are not only valuable for the early detection and staging of multi-organ fibrotic disorders, but also provide mechanistic insights and reveal novel therapeutic targets.

### Emerging technologies for key metabolites identification and targeted therapy

Beyond biomarker discovery, metabolomics is increasingly recognized as a powerful tool for identifying therapeutic targets and guiding precision interventions. Recent technological advances are shifting the field from static metabolite quantification towards dynamic, spatial and multi-dimensional analyses that directly inform therapeutic development.

Spatial and single-cell metabolomics are revolutionizing our understanding of metabolic heterogeneity. Unlike conventional metabolomics that averages signals across a tissue, mass spectrometry imaging (MSI) preserves the spatial context of metabolites within tissues, revealing localized metabolic reprogramming. Spatial lipidomics has identified zone-specific accumulation of saturated FAs in fibrotic niches of MASH livers [247]. Similarly, MSI has revealed glycogen accumulation in myoFbs as an actionable target in pulmonary fibrosis. The integration of MSI with isotope tracing further enables spatially resolved metabolic flux analysis, allowing researchers to pinpoint where and how drugs interfere with specific metabolic pathways in situ [248]. In kidney research, single-cell transcriptomics has mapped differential metabolic pathways across nephron segments, identifying cell-specific vulnerabilities [245].

Advanced computational and artificial intelligence (AI)-driven approaches are tackling the metabolite annotation bottleneck. The TidyMass2 framework performs feature-based functional module analysis by matching mass-to-charge ratios to a comprehensive human metabolic network. When applied to a pregnancy cohort, it increased biologically interpretable features from 5.8% to 58.8% [249]. The Lipidepifind tool uses metabolic network expansion to systematically identify structurally modified "epi-metabolites", uncovering novel lipid species in IPF serum absent from conventional databases [240]. AI is also accelerating drug target identification; dose–response metabolomics can infer the number of distinct molecular targets engaged by a drug, as demonstrated for the hepatotoxicity of etomoxir [248]. Furthermore, machine learning has been successfully applied to develop diagnostic panels for fibrosis progression in MASLD and SSc [243].

Stable isotope-resolved metabolomics (SIRM) provides dynamic insights essential for target validation. By tracing ^13^C-labeled substrates through metabolic networks, SIRM pinpoints rate-limiting enzymes driving disease phenotypes. In renal fibrosis, SIRM has shown that glutamine oxidation fuels collagen synthesis in myoFbs, identifying glutaminase as a therapeutic target [148]. Flux studies in failing hearts have demonstrated altered substrate switching between FAs and glucose, informing the development of metabolic modulators [250].

Multi-omics integration provides systems-level insights. Combining metabolomics with transcriptomics and proteomics has identified novel disease drivers, such as the myo-inositol/SMIT1 axis in adverse cardiac remodeling [239]. In CKD, integrating urinary proteomics with metabolomics has validated peptide panels that reflect underlying enzymatic activities including collagen turnover [245]. Furthermore, combined microbiome-metabolomics analyses have revealed gut microbial metabolites, including trimethylamine N-oxide (TMAO) and indoxyl sulfate, as key mediators of renal and cardiac fibrosis [12, 250]. This integrated approach enables the validation of therapeutic efficacy in preclinical models by linking metabolite changes directly to target engagement.

Collectively, these emerging technologies including spatial and single-cell metabolomics, AI-driven network analysis, SIRM, and multi-omics integration, are transforming the field from descriptive biomarker discovery to hypothesis-driven target identification. They enable the precise mapping of metabolic dysregulation across organs, cell types and disease stages, accelerating the development of metabolism-targeted therapies for fibrotic diseases.

## Conclusions and perspectives

Fibrosis has emerged as a global issue affecting the functions of multiple organs. In recent years, metabolic reprogramming has been recognized as a significant characteristic of the fibrotic process. In this review, we summarize the roles of enzymes that not only perform catalytic functions but also undergo PTMs, together with lipid metabolic reprogramming and glutamine metabolism, in the progression of fibrosis across multiple organs and tissues, including the heart, lungs, liver, kidneys, skin, peritoneum and various glands, with a particular emphasis on the nonmetabolic functions of key enzymes. The debate on whether metabolic remodelling, such as increased glycolysis, is a driver or a bystander phenomenon is central to understanding its therapeutic potential. While extensive literature supports that enhanced glycolysis plays a causal role in fibrogenesis, emerging evidence suggests that its contribution is context-dependent, varying across cell types, fibrotic stages and experimental models. For instance, the observation that LDH5 inhibition did not significantly alter fibrosis-associated markers in certain pulmonary fibrosis models. Therefore, rather than viewing glycolysis as a universal and absolute driver, we propose a more nuanced perspective: increased glycolysis is a critical but context-dependent facilitator of fibrosis, with its role shaped by the specific cellular microenvironment and disease stage. Accumulating evidence has shown that the direct manipulation of key glycolytic enzymes, including PKM2, HK2 and PFKFB3, is sufficient to modulate myoFb activation and collagen production in vivo. Mechanistically, glycolysis fuels fibrosis by providing essential biosynthetic precursors for collagen and lipid metabolism and by generating lactate, which serves as a signalling molecule to stabilize transcription factors such as HIF-1α and promote profibrotic epigenetic modifications. Crucially, glycolysis engages in a self-reinforcing positive feedback loop with master regulators such as TGF-β; when TGF-β induces the expression of glycolytic enzymes, the resulting metabolic reprogramming is essential for sustaining the TGF-β-driven profibrotic transcriptional program. A similarly pivotal role for lipid metabolic remodeling in fibrosis is emerging. TGF-β also upregulates the expression of lipogenic enzymes such as ACC and FASN, whose products are essential for maintaining a persistent profibrotic phenotype and excessive ECM production. Thus, metabolic reprogramming is a necessary upstream event that actively promotes the fibrotic cascade, making it a promising therapeutic target.

The regulation of metabolic networks is exquisitely intricate. The functional diversity of enzymes across organs and cell types is dictated by variations in their expression levels, subcellular localization, isoform distribution, and PTMs. A pertinent example is the role of PKM2, which, despite promising implications in organ fibrosis, presents considerable challenges for clinical translation. A primary challenge arises from the high structural conservation of the ATP binding site across PK isoforms. This homology complicates the development of PKM2-selective inhibitors, as many compounds inadvertently suppress PK activity, raising concerns about potential hepatotoxicity and hemolysis. Furthermore, many current PKM2 modulators—whether inhibitors or activators—are hampered by poor pharmaceutical properties, including low solubility, inadequate bioavailability, and significant toxicity, confining most candidates to preclinical studies. Therefore, minimizing side effects presents a considerable challenge in drug development.

The broadly tested glycolysis inhibitor 2-deoxy-D-glucose (2-DG) is a glucose gueanalog that competes with glucose for cellular uptake via GLUT transporters. Once inside cells, 2-DG is phosphorylated by HK to generate 2-deoxy-D-glucose-6-phosphate (2-DG-6P). This metabolite cannot be further processed by phosphoglucose isomerase, leading to the accumulation and subsequent inhibition of glycolysis [251]. Despite this promising mechanism, its clinical translation faces hurdles because of its short half-life, rapid metabolism, and poor potency, and thus high concentrations are required(≥ 5 mmol/L) to compete with blood glucose for cellular uptake. However, clinical studies have identified a potential therapeutic window. Doses up to 63 mg/kg were well tolerated, and a phase II trial in COVID-19 patients confirmed the general safety of doses up to 126 mg/kg/day, with hyperglycemia being the most common side effect [252]. While higher doses can cause symptoms of glucopenia and other adverse events, the established safety profile of moderate doses supports its potential for further development as an antiglycolytic therapy. Current strategies to minimize systemic exposure and improve therapeutic safety include “isoform-restricted” targeting of specific subtypes and nanoparticle-based delivery systems, both of which require a careful evaluation of their therapeutic safety windows [195].

Given that metabolic reprogramming occurs in fibrosis in multiple organs, we anticipate that targeting these alterations, such as by suppressing aberrant glycolysis, restoring impaired lipid oxidation, and stabilizing cellular metabolic homeostasis, may represent a promising direction for antifibrotic therapy and drug development.

## Data Availability

Not applicable.

## References

[1] Deng Q, Wu L, Zhang C, Dang M. Global fibrosis burden and a transcriptional biomarker-based strategy for early detection in resource-limited settings. Biomolecules. 2025. 10.3390/biom15091273. 41008580 10.3390/biom15091273 PMC12467191

[2] Zhao X, Kwan JYY, Yip K, Liu PP, Liu FF. Targeting metabolic dysregulation for fibrosis therapy. Nat Rev Drug Discov. 2020;19(1):57–75. 10.1038/s41573-019-0040-5. 31548636 10.1038/s41573-019-0040-5

[3] Noom A, Sawitzki B, Knaus P, Duda GN. A two-way street - cellular metabolism and myofibroblast contraction. npj Regen Med. 2024;9(1):15. 10.1038/s41536-024-00359-x. 38570493 10.1038/s41536-024-00359-x PMC10991391

[4] Younesi FS, Miller AE, Barker TH, Rossi FMV, Hinz B. Fibroblast and myofibroblast activation in normal tissue repair and fibrosis. Nat Rev Mol Cell Biol. 2024. 10.1038/s41580-024-00716-0. 38589640 10.1038/s41580-024-00716-0

[5] Henderson J, O’Reilly S. The emerging role of metabolism in fibrosis. Trends Endocrinol Metab. 2021;32(8):639–53. 10.1016/j.tem.2021.05.003. 34024695 10.1016/j.tem.2021.05.003

[6] Sun W, Zhou S, Peng L, Wang W, Liu Y, Wang T, et al. Fatty Acid Oxidation-Glycolysis Metabolic Transition Affects ECM Homeostasis in Silica-Induced Pulmonary Fibrosis. Adv Sci (Weinh). 2025;12(7):e2407134. 10.1002/advs.202407134. 39721015 10.1002/advs.202407134 PMC11831484

[7] Stocks B, Zierath JR. Post-translational modifications: the signals at the intersection of exercise, glucose uptake, and insulin sensitivity. Endocr Rev. 2022;43(4):654–77. 10.1210/endrev/bnab038. 34730177 10.1210/endrev/bnab038 PMC9277643

[8] Liu Z, Li A, Ma Z, Wang J, Chen X, Wang Z, et al. Lactate metabolism and protein lactylation in cancer. Mol Biomed. 2026. 10.1186/s43556-026-00417-4. 41746580 10.1186/s43556-026-00417-4 PMC12946641

[9] Zhang W, Zhu X, Zhang Y, Yang J, Sun X, Wang J, et al. Lactylation: a metabolic-epigenetic bridge in diabetic kidney disease and a therapeutic target for TCM. Chin Med. 2026. 10.1186/s13020-026-01361-9. 41787542 10.1186/s13020-026-01361-9 PMC12964770

[10] Certo M, Llibre A, Lee W, Mauro C. Understanding lactate sensing and signalling. Trends Endocrinol Metab. 2022;33(10):722–35. 10.1016/j.tem.2022.07.004. 35999109 10.1016/j.tem.2022.07.004

[11] Liu ZY, Song K, Tu B, Lin LC, Sun H, Zhou Y, et al. Glycolytic reprogramming in organ fibrosis: new dynamics of the epigenetic landscape. Free Radic Biol Med. 2023;207:1–10. 10.1016/j.freeradbiomed.2023.07.003. 37419215 10.1016/j.freeradbiomed.2023.07.003

[12] Steinberg GR, Carpentier AC, Wang D. MASH: the nexus of metabolism, inflammation, and fibrosis. J Clin Invest. 2025. 10.1172/jci186420. 40955659 10.1172/JCI186420 PMC12435842

[13] Chandel NS. Glycolysis. Cold Spring Harb Perspect Biol. 2021. 10.1101/cshperspect.a040535. 33941515 10.1101/cshperspect.a040535 PMC8091952

[14] Andreadou I, Ghigo A, Nikolaou PE, Swirski FK, Thackeray JT, Heusch G, et al. Immunometabolism in heart failure. Nat Rev Cardiol. 2025;22(10):751–72. 10.1038/s41569-025-01165-8. 40544171 10.1038/s41569-025-01165-8

[15] Bian X, Jiang H, Meng Y, Li YP, Fang J, Lu Z. Regulation of gene expression by glycolytic and gluconeogenic enzymes. Trends Cell Biol. 2022;32(9):786–99. 10.1016/j.tcb.2022.02.003. 35300892 10.1016/j.tcb.2022.02.003

[16] Horn P, Tacke F. Metabolic reprogramming in liver fibrosis. Cell Metab. 2024;36(7):1439–55. 10.1016/j.cmet.2024.05.003. 38823393 10.1016/j.cmet.2024.05.003

[17] Sun Q, Karwi QG, Wong N, Lopaschuk GD. Advances in myocardial energy metabolism: metabolic remodelling in heart failure and beyond. Cardiovasc Res. 2024;120(16):1996–2016. 10.1093/cvr/cvae231. 39453987 10.1093/cvr/cvae231 PMC11646102

[18] Luo H, Guo S, Chu Y, Xin Y, Yin X, Li X. Metabolism and Targeted Therapy of Fibrosis in Chronic Pancreatitis: A Review. Int J Med Sci. 2025;22(14):3528–42. 10.7150/ijms.118338. 40959561 10.7150/ijms.118338 PMC12434811

[19] Gibb AA, Murray EK, Huynh AT, Gaspar RB, Ploesch TL, Bedi K, et al. Glutaminolysis is Essential for Myofibroblast Persistence and In Vivo Targeting Reverses Fibrosis and Cardiac Dysfunction in Heart Failure. Circulation. 2022;145(21):1625–8. 10.1161/circulationaha.121.057879. 35605036 10.1161/CIRCULATIONAHA.121.057879 PMC9179236

[20] Kumar MA, Baba SK, Khan IR, Khan MS, Husain FM, Ahmad S, et al. Glutamine metabolism: molecular regulation, biological functions, and diseases. MedComm (2020). 2025;6(7):e70120. 10.1002/mco2.70120. 40567251 10.1002/mco2.70120 PMC12188105

[21] Martínez-Reyes I, Chandel NS. Mitochondrial TCA cycle metabolites control physiology and disease. Nat Commun. 2020;11(1):102. 10.1038/s41467-019-13668-3. 31900386 10.1038/s41467-019-13668-3 PMC6941980

[22] Alhasan KA, King MA, Pattar BSB, Lewis IA, Lopaschuk GD, Greenway SC. Anaplerotic filling in heart failure: a review of mechanism and potential therapeutics. Cardiovasc Res. 2024;120(17):2166–78. 10.1093/cvr/cvae248. 39570879 10.1093/cvr/cvae248 PMC11687400

[23] Sarkar S, Chang CI, Jean J, Wu MJ. TCA cycle-derived oncometabolites in cancer and the immune microenvironment. J Biomed Sci. 2025;32(1):87. 10.1186/s12929-025-01186-y. 40931345 10.1186/s12929-025-01186-y PMC12424225

[24] Ma R, Lu D, Yang Z, Ji X, Tian R, Xu F, et al. Emerging therapy strategies for energy metabolism in acute myocardial infarction. J Transl Med. 2025;23(1):1140. 10.1186/s12967-025-07088-9. 41121256 10.1186/s12967-025-07088-9 PMC12538951

[25] Lei Y, Cai S, Zhang JK, Ding SQ, Zhang ZH, Zhang CD, et al. The role and mechanism of fatty acid oxidation in cancer drug resistance. Cell Death Discov. 2025;11(1):277. 10.1038/s41420-025-02554-1. 40514365 10.1038/s41420-025-02554-1 PMC12166077

[26] Dembitz V, James SC, Gallipoli P. Targeting lipid metabolism in acute myeloid leukemia: biological insights and therapeutic opportunities. Leukemia. 2025;39(8):1814–23. 10.1038/s41375-025-02645-z. 40404984 10.1038/s41375-025-02645-z PMC12310549

[27] Hu T, Liu CH, Lei M, Zeng Q, Li L, Tang H, et al. Metabolic regulation of the immune system in health and diseases: mechanisms and interventions. Signal Transduct Target Ther. 2024;9(1):268. 10.1038/s41392-024-01954-6. 39379377 10.1038/s41392-024-01954-6 PMC11461632

[28] Zhao M, Wang L, Wang M, Zhou S, Lu Y, Cui H, et al. Targeting fibrosis, mechanisms and cilinical trials. Signal Transduct Target Ther. 2022;7(1):206. 10.1038/s41392-022-01070-3. 35773269 10.1038/s41392-022-01070-3 PMC9247101

[29] Lin LC, Liu ZY, Yang JJ, Zhao JY, Tao H. Lipid metabolism reprogramming in cardiac fibrosis. Trends Endocrinol Metab. 2024;35(2):164–75. 10.1016/j.tem.2023.10.004. 37949734 10.1016/j.tem.2023.10.004

[30] Liu P, Liu ZY, Mao S, Shen XY, Liu ZY, Lin LC, et al. Targeted mitochondrial function for cardiac fibrosis: An epigenetic perspective. Free Radic Biol Med. 2025;228:163–72. 10.1016/j.freeradbiomed.2025.01.001. 39755218 10.1016/j.freeradbiomed.2025.01.001

[31] Nederlof R, Eerbeek O, Hollmann MW, Southworth R, Zuurbier CJ. Targeting hexokinase II to mitochondria to modulate energy metabolism and reduce ischaemia-reperfusion injury in heart. Br J Pharmacol. 2014;171(8):2067–79. 10.1111/bph.12363. 24032601 10.1111/bph.12363 PMC3976622

[32] Pasdois P, Parker JE, Halestrap AP. Extent of mitochondrial hexokinase II dissociation during ischemia correlates with mitochondrial cytochrome c release, reactive oxygen species production, and infarct size on reperfusion. J Am Heart Assoc. 2012;2(1):e005645. 10.1161/jaha.112.005645. 23525412 10.1161/JAHA.112.005645 PMC3603240

[33] Chen S, Zou Y, Song C, Cao K, Cai K, Wu Y, et al. The role of glycolytic metabolic pathways in cardiovascular disease and potential therapeutic approaches. Basic Res Cardiol. 2023;118(1):48. 10.1007/s00395-023-01018-w. 37938421 10.1007/s00395-023-01018-w PMC10632287

[34] Zhang Y, Cheng X, Wang Y, Guo H, Song Y, Wang H, et al. Phlorizin ameliorates myocardial fibrosis by inhibiting pyroptosis through restraining HK1-mediated NLRP3 inflammasome activation. Heliyon. 2023;9(11):e21217. 10.1016/j.heliyon.2023.e21217. 38027628 10.1016/j.heliyon.2023.e21217 PMC10658207

[35] McCommis KS, Douglas DL, Krenz M, Baines CP. Cardiac-specific hexokinase 2 overexpression attenuates hypertrophy by increasing pentose phosphate pathway flux. J Am Heart Assoc. 2013;2(6):e000355. 10.1161/jaha.113.000355. 24190878 10.1161/JAHA.113.000355 PMC3886755

[36] Wu R, Wyatt E, Chawla K, Tran M, Ghanefar M, Laakso M, et al. Hexokinase II knockdown results in exaggerated cardiac hypertrophy via increased ROS production. EMBO Mol Med. 2012;4(7):633–46. 10.1002/emmm.201200240. 22517678 10.1002/emmm.201200240 PMC3407950

[37] Wang F, Yin X, Fan YM, Zhang X, Ma C, Jia K, et al. Upregulation of glycolytic enzyme PFKFB3 by deubiquitinase OTUD4 promotes cardiac fibrosis post myocardial infarction. J Mol Med (Berl). 2023;101(6):743–56. 10.1007/s00109-023-02323-6. 37162556 10.1007/s00109-023-02323-6 PMC10234888

[38] Zou X, Ouyang H, Lin F, Zhang H, Yang Y, Pang D, et al. MYBPC3 deficiency in cardiac fibroblasts drives their activation and contributes to fibrosis. Cell Death Dis. 2022;13(11):948. 10.1038/s41419-022-05403-6. 36357371 10.1038/s41419-022-05403-6 PMC9649783

[39] He X, Zeng H, Cantrell AC, Williams QA, Chen JX. Knockout of TIGAR enhances myocardial phosphofructokinase activity and preserves diastolic function in heart failure. J Cell Physiol. 2022;237(8):3317–27. 10.1002/jcp.30790. 35621078 10.1002/jcp.30790 PMC9378637

[40] He X, Williams QA, Cantrell AC, Besanson J, Zeng H, Chen JX. TIGAR deficiency blunts Angiotensin-II-induced cardiac hypertrophy in mice. Int J Mol Sci. 2024. 10.3390/ijms25042433. 38397106 10.3390/ijms25042433 PMC10889085

[41] Wang C, Qiao S, Zhao Y, Tian H, Yan W, Hou X, et al. The KLF7/PFKL/ACADL axis modulates cardiac metabolic remodelling during cardiac hypertrophy in male mice. Nat Commun. 2023;14(1):959. 10.1038/s41467-023-36712-9. 36810848 10.1038/s41467-023-36712-9 PMC9944323

[42] Lee YB, Min JK, Kim JG, Cap KC, Islam R, Hossain AJ, et al. Multiple functions of pyruvate kinase M2 in various cell types. J Cell Physiol. 2022;237(1):128–48. 10.1002/jcp.30536. 34311499 10.1002/jcp.30536

[43] Hauck L, Dadson K, Chauhan S, Grothe D, Billia F. Inhibiting the Pkm2/b-catenin axis drives in vivo replication of adult cardiomyocytes following experimental MI. Cell Death Differ. 2021;28(4):1398–417. 10.1038/s41418-020-00669-9. 33288902 10.1038/s41418-020-00669-9 PMC8027412

[44] Magadum A, Singh N, Kurian AA, Munir I, Mehmood T, Brown K, et al. Pkm2 Regulates Cardiomyocyte Cell Cycle and Promotes Cardiac Regeneration. Circulation. 2020;141(15):1249–65. 10.1161/circulationaha.119.043067. 32078387 10.1161/CIRCULATIONAHA.119.043067 PMC7241614

[45] Wu X, Liu L, Zheng Q, Hao H, Ye H, Li P, et al. Protocatechuic aldehyde protects cardiomycoytes against ischemic injury via regulation of nuclear pyruvate kinase M2. Acta Pharmaceutica Sinica B. 2021;11(11):3553–66. 10.1016/j.apsb.2021.03.021. 34900536 10.1016/j.apsb.2021.03.021 PMC8642444

[46] Sharma S, Watanabe T, Nishimoto T, Takihara T, Mlakar L, Nguyen XX, et al. E4 engages uPAR and enolase-1 and activates urokinase to exert antifibrotic effects. JCI Insight. 2021. 10.1172/jci.insight.144935. 34935642 10.1172/jci.insight.144935 PMC8783693

[47] Zhang X, Zheng C, Gao Z, Wang L, Chen C, Zheng Y, et al. PKM2 promotes angiotensin-II-induced cardiac remodelling by activating TGF-β/Smad2/3 and Jak2/Stat3 pathways through oxidative stress. J Cell Mol Med. 2021;25(22):10711–23. 10.1111/jcmm.17007. 34687136 10.1111/jcmm.17007 PMC8581335

[48] Ni L, Lin B, Hu L, Zhang R, Fu F, Shen M, et al. Pyruvate kinase M2 protects heart from pressure overload-induced heart failure by phosphorylating RAC1. J Am Heart Assoc. 2022;11(11):e024854. 10.1161/jaha.121.024854. 35656980 10.1161/JAHA.121.024854 PMC9238738

[49] Lorenzana-Carrillo MA, Gopal K, Byrne NJ, Tejay S, Saleme B, Das SK, et al. TRIM35-mediated degradation of nuclear PKM2 destabilizes GATA4/6 and induces P53 in cardiomyocytes to promote heart failure. Sci Transl Med. 2022;14(669):eabm3565. 10.1126/scitranslmed.abm3565. 36322626 10.1126/scitranslmed.abm3565

[50] Zhou J, Zhang S, Chen Z, He Z, Xu Y, Li Z. CircRNA-ENO1 promoted glycolysis and tumor progression in lung adenocarcinoma through upregulating its host gene ENO1. Cell Death Dis. 2019;10(12):885. 10.1038/s41419-019-2127-7. 31767835 10.1038/s41419-019-2127-7 PMC6877563

[51] Ji JJ, Qian LL, Zhu Y, Jiang Y, Guo JQ, Wu Y, et al. Kallistatin/Serpina3c inhibits cardiac fibrosis after myocardial infarction by regulating glycolysis via Nr4a1 activation. Biochim Biophys Acta Mol Basis Dis. 2022;1868(9):166441. 10.1016/j.bbadis.2022.166441. 35577178 10.1016/j.bbadis.2022.166441

[52] Wu Y, Qin YH, Liu Y, Zhu L, Zhao XX, Liu YY, et al. Cardiac troponin I autoantibody induces myocardial dysfunction by PTEN signaling activation. EBioMedicine. 2019;47:329–40. 10.1016/j.ebiom.2019.08.045. 31474552 10.1016/j.ebiom.2019.08.045 PMC6796505

[53] Iida-Norita R, Miyata H, Kaneda Y, Emori C, Noda T, Nakagawa T, et al. Generation of humanized LDHC knock-in mice as a tool to assess human LDHC-targeting contraceptive drugs. Andrology. 2023;11(5):840–8. 10.1111/andr.13359. 36464740 10.1111/andr.13359

[54] Sharma D, Singh M, Rani R. Role of LDH in tumor glycolysis: regulation of LDHA by small molecules for cancer therapeutics. Semin Cancer Biol. 2022;87:184–95. 10.1016/j.semcancer.2022.11.007. 36371026 10.1016/j.semcancer.2022.11.007

[55] Luengo A, Li Z, Gui DY, Sullivan LB, Zagorulya M, Do BT, et al. Increased demand for NAD(+) relative to ATP drives aerobic glycolysis. Mol Cell. 2021;81(4):691-707.e696. 10.1016/j.molcel.2020.12.012. 33382985 10.1016/j.molcel.2020.12.012 PMC8315838

[56] Gong H, Zhong H, Cheng L, Li LP, Zhang DK. Post-translational protein lactylation modification in health and diseases: a double-edged sword. J Transl Med. 2024;22(1):41. 10.1186/s12967-023-04842-9. 38200523 10.1186/s12967-023-04842-9 PMC10777551

[57] Baartscheer A, Schumacher CA, van Borren MM, Belterman CN, Coronel R, Fiolet JW. Increased Na+/H+-exchange activity is the cause of increased [Na+]i and underlies disturbed calcium handling in the rabbit pressure and volume overload heart failure model. Cardiovasc Res. 2003;57(4):1015–24. 10.1016/s0008-6363(02)00809-x. 12650879 10.1016/s0008-6363(02)00809-x

[58] Li H, Chen J, Xing X, Lou D. Association of lactate detection with in-hospital mortality in critically ill patients with acute myocardial infarction: a retrospective cohort study. BMJ Open. 2023;13(4):e069129. 10.1136/bmjopen-2022-069129. 37085300 10.1136/bmjopen-2022-069129 PMC10124257

[59] Fan M, Yang K, Wang X, Chen L, Gill PS, Ha T, et al. Lactate promotes endothelial-to-mesenchymal transition via Snail1 lactylation after myocardial infarction. Sci Adv. 2023;9(5):eadc9465. 10.1126/sciadv.adc9465. 36735787 10.1126/sciadv.adc9465 PMC9897666

[60] Wang N, Wang W, Wang X, Mang G, Chen J, Yan X, et al. Histone lactylation boosts reparative gene activation post-myocardial infarction. Circ Res. 2022;131(11):893–908. 10.1161/circresaha.122.320488. 36268709 10.1161/CIRCRESAHA.122.320488

[61] Wei T, Guo Y, Huang C, Sun M, Zhou B, Gao J, et al. Fibroblast-to-cardiomyocyte lactate shuttle modulates hypertensive cardiac remodelling. Cell Biosci. 2023;13(1):151. 10.1186/s13578-023-01098-0. 37580825 10.1186/s13578-023-01098-0 PMC10426103

[62] Gizak A, McCubrey JA, Rakus D. Cell-to-cell lactate shuttle operates in heart and is important in age-related heart failure. Aging (Albany NY). 2020;12(4):3388–406. 10.18632/aging.102818. 32035422 10.18632/aging.102818 PMC7066931

[63] Hou Z, Yan W, Li T, Wu W, Cui Y, Zhang X, et al. Lactic acid-mediated endothelial to mesenchymal transition through TGF-β1 contributes to in-stent stenosis in poly-L-lactic acid stent. Int J Biol Macromol. 2020;155:1589–98. 10.1016/j.ijbiomac.2019.11.136. 31770555 10.1016/j.ijbiomac.2019.11.136

[64] Zhang N, Zhang Y, Xu J, Wang P, Wu B, Lu S, et al. α-myosin heavy chain lactylation maintains sarcomeric structure and function and alleviates the development of heart failure. Cell Res. 2023;33(9):679–98. 10.1038/s41422-023-00844-w. 37443257 10.1038/s41422-023-00844-w PMC10474270

[65] Du L, Wang X, Guo Y, Tao T, Wu H, Xu X, et al. Altered lipid metabolism promoting cardiac fibrosis is mediated by CD34(+) cell-derived FABP4(+) fibroblasts. Exp Mol Med. 2024;56(8):1869–86. 10.1038/s12276-024-01309-9. 39198543 10.1038/s12276-024-01309-9 PMC11372182

[66] Glatz JFC, Heather LC, Luiken J. CD36 as a gatekeeper of myocardial lipid metabolism and therapeutic target for metabolic disease. Physiol Rev. 2024;104(2):727–64. 10.1152/physrev.00011.2023. 37882731 10.1152/physrev.00011.2023

[67] Wang HF, Wang YX, Zhou YP, Wei YP, Yan Y, Zhang ZJ, et al. Protein O-GlcNAcylation in cardiovascular diseases. Acta Pharmacol Sin. 2023;44(1):8–18. 10.1038/s41401-022-00934-2. 35817809 10.1038/s41401-022-00934-2 PMC9813366

[68] Zhao K, Yan L, Sun X, Hu X. O-GlcNAc transferase-mediated O-GlcNAcylation of CD36 against myocardial ischemia-reperfusion injury. Tissue Cell. 2025;95:102878. 10.1016/j.tice.2025.102878. 40154105 10.1016/j.tice.2025.102878

[69] Zhang Q, Li J, Liu X, Chen X, Zhu L, Zhang Z, et al. Inhibiting CD36 palmitoylation improves cardiac function post-infarction by regulating lipid metabolic homeostasis and autophagy. Nat Commun. 2025;16(1):6602. 10.1038/s41467-025-61875-y. 40675975 10.1038/s41467-025-61875-y PMC12271414

[70] Seferović PM, Paulus WJ, Rosano G, Polovina M, Petrie MC, Jhund PS, et al. Diabetic myocardial disorder. A clinical consensus statement of the Heart Failure Association of the ESC and the ESC Working Group on Myocardial & Pericardial Diseases. Eur J Heart Fail. 2024;26(9):1893–903. 10.1002/ejhf.3347. 38896048 10.1002/ejhf.3347

[71] Wang S, Han Y, Liu R, Hou M, Neumann D, Zhang J, et al. Glycolysis-Mediated Activation of v-ATPase by Nicotinamide Mononucleotide Ameliorates Lipid-Induced Cardiomyopathy by Repressing the CD36-TLR4 Axis. Circ Res. 2024;134(5):505–25. 10.1161/circresaha.123.322910. 38422177 10.1161/CIRCRESAHA.123.322910 PMC10906217

[72] Dennis K, Gopal K, Montes Aparicio CN, Zhang JA, Castro-Guarda M, Nicol T, et al. FoxO1-zDHHC4-CD36 S-acylation axis drives metabolic dysfunction in Diabetes. Circ Res. 2025;136(12):1545–60. 10.1161/circresaha.124.325918. 40357580 10.1161/CIRCRESAHA.124.325918 PMC12136392

[73] Lazaropoulos MP, Gibb AA, Chapski DJ, Nair AA, Reiter AN, Roy R, et al. Nuclear ATP-citrate lyase regulates chromatin-dependent activation and maintenance of the myofibroblast gene program. Nat Cardiovasc Res. 2024;3(7):869–82. 10.1038/s44161-024-00502-3. 39196175 10.1038/s44161-024-00502-3 PMC11358007

[74] Kuwahara N, Nagao M, Shinohara M, Kaneshiro K, Emoto T, Yoshida T, et al. ACLY promotes cardiac fibrosis via the regulation of DNL and histone acetylation. Hypertension. 2025;82(6):1116–28. 10.1161/hypertensionaha.124.24088. 40047081 10.1161/HYPERTENSIONAHA.124.24088

[75] Li Y, Lou Z, Liu F, Liu Y, Wang C, Wang Y, et al. The impact of lipid metabolism on ferroptosis in myocardial ischemia-reperfusion injury. Apoptosis. 2025. 10.1007/s10495-025-02192-z. 41047443 10.1007/s10495-025-02192-z

[76] Raghu G, Remy-Jardin M, Richeldi L, Thomson CC, Inoue Y, Johkoh T, et al. Idiopathic Pulmonary Fibrosis (an update) and Progressive Pulmonary Fibrosis in adults: An official ATS/ERS/JRS/ALAT clinical practice guideline. Am J Respir Crit Care Med. 2022;205(9):e18–47. 10.1164/rccm.202202-0399ST. 35486072 10.1164/rccm.202202-0399ST PMC9851481

[77] Dai T, Liang Y, Li X, Zhao J, Li G, Li Q, et al. Targeting alveolar epithelial cell metabolism in pulmonary fibrosis: pioneering an emerging therapeutic strategy. Front Cell Dev Biol. 2025;13:1608750. 10.3389/fcell.2025.1608750. 40636669 10.3389/fcell.2025.1608750 PMC12238887

[78] Roque W, Romero F. Cellular metabolomics of pulmonary fibrosis, from amino acids to lipids. Am J Physiol Cell Physiol. 2021;320(5):C689-c695. 10.1152/ajpcell.00586.2020. 33471621 10.1152/ajpcell.00586.2020 PMC8163573

[79] Umeda Y, Demura Y, Morikawa M, Anzai M, Kadowaki M, Ameshima S, et al. Prognostic value of dual-time-point 18F-FDG PET for Idiopathic Pulmonary Fibrosis. J Nucl Med. 2015;56(12):1869–75. 10.2967/jnumed.115.163360. 26359263 10.2967/jnumed.115.163360

[80] Yan P, Liu J, Li Z, Wang J, Zhu Z, Wang L, et al. Glycolysis reprogramming in Idiopathic Pulmonary Fibrosis: unveiling the mystery of lactate in the lung. Int J Mol Sci. 2023. 10.3390/ijms25010315. 38203486 10.3390/ijms25010315 PMC10779333

[81] Newton DA, Lottes RG, Ryan RM, Spyropoulos DD, Baatz JE. Dysfunctional lactate metabolism in human alveolar type II cells from idiopathic pulmonary fibrosis lung explant tissue. Respir Res. 2021;22(1):278. 10.1186/s12931-021-01866-x. 34711218 10.1186/s12931-021-01866-x PMC8554831

[82] Rao X, Zhou D, Deng H, Chen Y, Wang J, Zhou X, et al. Activation of NLRP3 inflammasome in lung epithelial cells triggers radiation-induced lung injury. Respir Res. 2023;24(1):25. 10.1186/s12931-023-02331-7. 36694200 10.1186/s12931-023-02331-7 PMC9872296

[83] Wang P, Xie D, Xiao T, Cheng C, Wang D, Sun J, et al. H3K18 lactylation promotes the progression of arsenite-related idiopathic pulmonary fibrosis via YTHDF1/m6A/NREP. J Hazard Mater. 2024;461:132582. 10.1016/j.jhazmat.2023.132582. 37742376 10.1016/j.jhazmat.2023.132582

[84] Satyanarayana G, Turaga RC, Sharma M, Wang S, Mishra F, Peng G, et al. Pyruvate kinase M2 regulates fibrosis development and progression by controlling glycine auxotrophy in myofibroblasts. Theranostics. 2021;11(19):9331–41. 10.7150/thno.60385. 34646373 10.7150/thno.60385 PMC8490528

[85] van de Wetering C, Manuel AM, Sharafi M, Aboushousha R, Qian X, Erickson C, et al. Glutathione-S-transferase P promotes glycolysis in asthma in association with oxidation of pyruvate kinase M2. Redox Biol. 2021;47:102160. 10.1016/j.redox.2021.102160. 34624602 10.1016/j.redox.2021.102160 PMC8502950

[86] Mei S, Xu Q, Hu Y, Tang R, Feng J, Zhou Y, et al. Integrin β3-PKM2 pathway-mediated aerobic glycolysis contributes to mechanical ventilation-induced pulmonary fibrosis. Theranostics. 2022;12(14):6057–68. 10.7150/thno.72328. 36168620 10.7150/thno.72328 PMC9475464

[87] Gu X, Meng H, Peng C, Lin S, Li B, Zhao L, et al. Inflammasome activation and metabolic remodelling in p16-positive aging cells aggravates high-fat diet-induced lung fibrosis by inhibiting NEDD4L-mediated K48-polyubiquitin-dependent degradation of SGK1. Clin Transl Med. 2023;13(6):e1308. 10.1002/ctm2.1308. 37345264 10.1002/ctm2.1308 PMC10285269

[88] Andrianifahanana M, Hernandez DM, Yin X, Kang JH, Jung MY, Wang Y, et al. Profibrotic up-regulation of glucose transporter 1 by TGF-β involves activation of MEK and mammalian target of rapamycin complex 2 pathways. Faseb j. 2016;30(11):3733–44. 10.1096/fj.201600428R. 27480571 10.1096/fj.201600428R PMC5067255

[89] Cho SJ, Moon JS, Lee CM, Choi AM, Stout-Delgado HW. Glucose Transporter 1-Dependent Glycolysis Is Increased during Aging-Related Lung Fibrosis, and Phloretin Inhibits Lung Fibrosis. Am J Respir Cell Mol Biol. 2017;56(4):521–31. 10.1165/rcmb.2016-0225OC. 27997810 10.1165/rcmb.2016-0225OC PMC5449513

[90] Xu Q, Cheng D, Li G, Liu Y, Li P, Sun W, et al. CircHIPK3 regulates pulmonary fibrosis by facilitating glycolysis in miR-30a-3p/FOXK2-dependent manner. Int J Biol Sci. 2021;17(9):2294–307. 10.7150/ijbs.57915. 34239356 10.7150/ijbs.57915 PMC8241722

[91] Zhang J, Chen W, Du J, Chu L, Zhou Z, Zhong W, et al. RNF130 protects against pulmonary fibrosis through suppressing aerobic glycolysis by mediating c-myc ubiquitination. Int Immunopharmacol. 2023;117:109985. 10.1016/j.intimp.2023.109985. 36893517 10.1016/j.intimp.2023.109985

[92] Yin X, Choudhury M, Kang JH, Schaefbauer KJ, Jung MY, Andrianifahanana M, et al. Hexokinase 2 couples glycolysis with the profibrotic actions of TGF-β. Sci Signal. 2019. 10.1126/scisignal.aax4067. 31848318 10.1126/scisignal.aax4067

[93] Shan B, Zhou H, Guo C, Liu X, Wu M, Zhai R, et al. Tanshinone IIA ameliorates energy metabolism dysfunction of pulmonary fibrosis using (13)C metabolic flux analysis. J Pharm Anal. 2024;14(2):244–58. 10.1016/j.jpha.2023.09.008. 38464785 10.1016/j.jpha.2023.09.008 PMC10921327

[94] Hu X, Xu Q, Wan H, Hu Y, Xing S, Yang H, et al. PI3K-Akt-mTOR/PFKFB3 pathway mediated lung fibroblast aerobic glycolysis and collagen synthesis in lipopolysaccharide-induced pulmonary fibrosis. Lab Invest. 2020;100(6):801–11. 10.1038/s41374-020-0404-9. 32051533 10.1038/s41374-020-0404-9

[95] Chen W, Zhang J, Zhong W, Liu Y, Lu Y, Zeng Z, et al. Anlotinib inhibits PFKFB3-driven glycolysis in myofibroblasts to reverse pulmonary fibrosis. Front Pharmacol. 2021;12:744826. 10.3389/fphar.2021.744826. 34603058 10.3389/fphar.2021.744826 PMC8481786

[96] Wang W, Zhang Y, Huang W, Yuan Y, Hong Q, Xie Z, et al. Alamandine/MrgD axis prevents TGF-β1-mediated fibroblast activation via regulation of aerobic glycolysis and mitophagy. J Transl Med. 2023;21(1):24. 10.1186/s12967-022-03837-2. 36635651 10.1186/s12967-022-03837-2 PMC9838062

[97] Lai X, Huang S, Lin Y, Qiu Y, Pu L, Lin S, et al. DACT2 protects against pulmonary fibrosis via suppressing glycolysis in lung myofibroblasts. Int J Biol Macromol. 2023;226:291–300. 10.1016/j.ijbiomac.2022.11.324. 36481337 10.1016/j.ijbiomac.2022.11.324

[98] Xie Y, Yang S, Xu Y, Gu P, Zhang Y, You X, et al. Interleukin-11 drives fibroblast metabolic reprogramming in crystalline silica-induced lung fibrosis. Sci Total Environ. 2024;949:174976. 10.1016/j.scitotenv.2024.174976. 39047838 10.1016/j.scitotenv.2024.174976

[99] Goodwin J, Choi H, Hsieh MH, Neugent ML, Ahn JM, Hayenga HN, et al. Targeting Hypoxia-Inducible Factor-1α/Pyruvate Dehydrogenase Kinase 1 axis by Dichloroacetate suppresses Bleomycin-induced pulmonary fibrosis. Am J Respir Cell Mol Biol. 2018;58(2):216–31. 10.1165/rcmb.2016-0186OC. 28915065 10.1165/rcmb.2016-0186OC PMC5805994

[100] Schruf E, Schroeder V, Kuttruff CA, Weigle S, Krell M, Benz M, et al. Human lung fibroblast-to-myofibroblast transformation is not driven by an LDH5-dependent metabolic shift towards aerobic glycolysis. Respir Res. 2019;20(1):87. 10.1186/s12931-019-1058-2. 31072408 10.1186/s12931-019-1058-2 PMC6507142

[101] Mo X, Yao H, Ju H, Wang M, Yi M, Lu Y, et al. PKM2 promotes glycolysis in alveolar macrophages and induces inflammation in Bronchopulmonary Dysplasia. Inflammation. 2026. 10.1007/s10753-026-02476-9. 41697433 10.1007/s10753-026-02476-9 PMC12963179

[102] Liu Z, Liu W, Wei H, Ping Y, Yu Z, Dong Z, et al. Elevated lactate production exacerbates PM2.5-induced pulmonary fibrosis by stabilizing TGF-β1. J Adv Res. 2025. 10.1016/j.jare.2025.07.057 10.1016/j.jare.2025.07.057 PMC13131523 40759348

[103] Cui H, Xie N, Banerjee S, Ge J, Jiang D, Dey T, et al. Lung myofibroblasts promote macrophage profibrotic activity through lactate-induced histone lactylation. Am J Respir Cell Mol Biol. 2021;64(1):115–25. 10.1165/rcmb.2020-0360OC. 33074715 10.1165/rcmb.2020-0360OC PMC7780997

[104] Summer R, Todd JL, Neely ML, Lobo LJ, Namen A, Newby LK, et al. Circulating metabolic profile in idiopathic pulmonary fibrosis: data from the IPF-PRO Registry. Respir Res. 2024;25(1):58. 10.1186/s12931-023-02644-7. 38273290 10.1186/s12931-023-02644-7 PMC10809477

[105] Liang J, Huang G, Liu X, Zhang X, Rabata A, Liu N, et al. Lipid deficiency contributes to impaired alveolar progenitor cell function in aging and Idiopathic Pulmonary Fibrosis. Am J Respir Cell Mol Biol. 2024;71(2):242–53. 10.1165/rcmb.2023-0290OC. 38657143 10.1165/rcmb.2023-0290OC PMC11299087

[106] Yang J, Pan X, Xu M, Li Y, Liang C, Liu L, et al. Downregulation of HMGCS2 mediated AECIIs lipid metabolic alteration promotes pulmonary fibrosis by activating fibroblasts. Respir Res. 2024;25(1):176. 10.1186/s12931-024-02816-z. 38658970 10.1186/s12931-024-02816-z PMC11040761

[107] Kwak D, Bradley PB, Subbotina N, Ling S, Teitz-Tennenbaum S, Osterholzer JJ, et al. CD36/Lyn kinase interactions within macrophages promotes pulmonary fibrosis in response to oxidized phospholipid. Respir Res. 2023;24(1):314. 10.1186/s12931-023-02629-6. 38098035 10.1186/s12931-023-02629-6 PMC10722854

[108] Wan HQ, Xie LF, Li HL, Ma Y, Li QH, Dai MQ, et al. GPR40 activation alleviates pulmonary fibrosis by repressing M2 macrophage polarization through the PKD1/CD36/TGF-β1 pathway. Acta Pharmacol Sin. 2025;46(10):2707–22. 10.1038/s41401-025-01558-y. 40369224 10.1038/s41401-025-01558-y PMC12460884

[109] Wang L, Yuan H, Li W, Yan P, Zhao M, Li Z, et al. ACSS3 regulates the metabolic homeostasis of epithelial cells and alleviates pulmonary fibrosis. Biochim Biophys Acta Mol Basis Dis. 2024;1870(2):166960. 10.1016/j.bbadis.2023.166960. 37979225 10.1016/j.bbadis.2023.166960

[110] Zheng H, Zhang L, Wang C, Wang Y, Zeng C. Metabolic dysregulation in pulmonary fibrosis: insights into amino acid contributions and therapeutic potential. Cell Death Discov. 2025;11(1):411. 10.1038/s41420-025-02715-2. 40858556 10.1038/s41420-025-02715-2 PMC12381058

[111] Contento G, Wilson JA, Selvarajah B, Platé M, Guillotin D, Morales V, et al. Pyruvate metabolism dictates fibroblast sensitivity to GLS1 inhibition during fibrogenesis. JCI Insight. 2024;9(18). 10.1172/jci.insight.178453 10.1172/jci.insight.178453 PMC11457851 39315548

[112] Wang S, Li X, Ma Q, Wang Q, Wu J, Yu H, et al. Glutamine metabolism is required for alveolar regeneration during lung injury. Biomolecules. 2022. 10.3390/biom12050728. 35625656 10.3390/biom12050728 PMC9138637

[113] Li G, Xu Q, Cheng D, Sun W, Liu Y, Ma D, et al. Caveolin-1 and Its Functional Peptide CSP7 Affect Silica-Induced Pulmonary Fibrosis by Regulating Fibroblast Glutaminolysis. Toxicol Sci. 2022;190(1):41–53. 10.1093/toxsci/kfac089. 36053221 10.1093/toxsci/kfac089

[114] Xiang Z, Bai L, Zhou JQ, Cevallos RR, Sanders JR, Liu G, et al. Epigenetic regulation of IPF fibroblast phenotype by glutaminolysis. Mol Metab. 2023;67:101655. 10.1016/j.molmet.2022.101655. 36526153 10.1016/j.molmet.2022.101655 PMC9827063

[115] Shaghaghi H, Para R, Tran C, Roman J, Ojeda-Lassalle Y, Sun J, et al. Glutamine restores mitochondrial respiration in bleomycin-injured epithelial cells. Free Radic Biol Med. 2021;176:335–44. 10.1016/j.freeradbiomed.2021.10.006. 34634441 10.1016/j.freeradbiomed.2021.10.006 PMC9121335

[116] Hammerich L, Tacke F. Hepatic inflammatory responses in liver fibrosis. Nat Rev Gastroenterol Hepatol. 2023;20(10):633–46. 10.1038/s41575-023-00807-x. 37400694 10.1038/s41575-023-00807-x

[117] Trivedi P, Wang S, Friedman SL. The Power of Plasticity-Metabolic Regulation of Hepatic Stellate Cells. Cell Metab. 2021;33(2):242–57. 10.1016/j.cmet.2020.10.026. 33232666 10.1016/j.cmet.2020.10.026 PMC7858232

[118] Shang Y, Sun Q, Xin X, Wang Z, Gao S, Lin R, et al. The role of glycolysis in MASLD development: Pathogenesis and therapeutic strategies. Pharmacol Res. 2025;221:107990. 10.1016/j.phrs.2025.107990. 41077164 10.1016/j.phrs.2025.107990

[119] Rho H, Terry AR, Chronis C, Hay N. Hexokinase 2-mediated gene expression via histone lactylation is required for hepatic stellate cell activation and liver fibrosis. Cell Metab. 2023;35(8):1406-1423.e1408. 10.1016/j.cmet.2023.06.013. 37463576 10.1016/j.cmet.2023.06.013 PMC11748916

[120] Loh Z, Fitzsimmons RL, Reid RC, Ramnath D, Clouston A, Gupta PK, et al. Inhibitors of class I histone deacetylases attenuate thioacetamide-induced liver fibrosis in mice by suppressing hepatic type 2 inflammation. Br J Pharmacol. 2019;176(19):3775–90. 10.1111/bph.14768. 31236923 10.1111/bph.14768 PMC6780048

[121] Wu X, Shen Y, Meng Y, Chen J, Zhang Y, Zeng S, et al. Suv39h1 contributes to activation of hepatic stellate cells in non-alcoholic fatty liver disease by enabling anaerobic glycolysis. Life Sci. 2024;341:122498. 10.1016/j.lfs.2024.122498. 38340980 10.1016/j.lfs.2024.122498

[122] Mejias M, Gallego J, Naranjo-Suarez S, Ramirez M, Pell N, Manzano A, et al. CPEB4 increases expression of PFKFB3 to induce glycolysis and activate mouse and human hepatic stellate cells, promoting liver fibrosis. Gastroenterology. 2020;159(1):273–88. 10.1053/j.gastro.2020.03.008. 32169429 10.1053/j.gastro.2020.03.008

[123] Zheng D, Jiang Y, Qu C, Yuan H, Hu K, He L, et al. Pyruvate kinase M2 tetramerization protects against hepatic stellate cell activation and liver fibrosis. Am J Pathol. 2020;190(11):2267–81. 10.1016/j.ajpath.2020.08.002. 32805235 10.1016/j.ajpath.2020.08.002 PMC7786052

[124] Guilliams M, Scott CL. Liver macrophages in health and disease. Immunity. 2022;55(9):1515–29. 10.1016/j.immuni.2022.08.002. 36103850 10.1016/j.immuni.2022.08.002

[125] Rao J, Wang H, Ni M, Wang Z, Wang Z, Wei S, et al. FSTL1 promotes liver fibrosis by reprogramming macrophage function through modulating the intracellular function of PKM2. Gut. 2022;71(12):2539–50. 10.1136/gutjnl-2021-325150. 35140065 10.1136/gutjnl-2021-325150 PMC9664121

[126] Xu F, Guo M, Huang W, Feng L, Zhu J, Luo K, et al. Annexin A5 regulates hepatic macrophage polarization via directly targeting PKM2 and ameliorates NASH. Redox Biol. 2020;36:101634. 10.1016/j.redox.2020.101634. 32863213 10.1016/j.redox.2020.101634 PMC7369618

[127] Li J, Chen X, Song S, Jiang W, Geng T, Wang T, et al. Hexokinase 2-mediated metabolic stress and inflammation burden of liver macrophages via histone lactylation in MASLD. Cell Rep. 2025;44(3):115350. 10.1016/j.celrep.2025.115350. 40014451 10.1016/j.celrep.2025.115350

[128] Dong T, Hu G, Fan Z, Wang H, Gao Y, Wang S, et al. Activation of GPR3-β-arrestin2-PKM2 pathway in Kupffer cells stimulates glycolysis and inhibits obesity and liver pathogenesis. Nat Commun. 2024;15(1):807. 10.1038/s41467-024-45167-5. 38280848 10.1038/s41467-024-45167-5 PMC10821868

[129] Xia Q, Sun D, Wan K, Liu C, Shu H, Wang M, et al. Lactylation: the metabolic-immune hub in autoimmune diseases. Front Immunol. 2025;16:1651923. 10.3389/fimmu.2025.1651923. 41488662 10.3389/fimmu.2025.1651923 PMC12756412

[130] Wang F, Chen L, Kong D, Zhang X, Xia S, Liang B, et al. Canonical Wnt signaling promotes HSC glycolysis and liver fibrosis through an LDH-A/HIF-1α transcriptional complex. Hepatology. 2024;79(3):606–23. 10.1097/hep.0000000000000569. 37733267 10.1097/HEP.0000000000000569 PMC10871634

[131] Li Y, Zhou Y, Xia S, Chen L, Yang T, Zhao D, et al. Blockade of KLF5/LDH-A feedback loop contributes to Curcumol inhibition of sinusoidal endothelial cell glycolysis and mitigation of liver fibrosis. Phytomedicine. 2023;114:154759. 10.1016/j.phymed.2023.154759. 37031640 10.1016/j.phymed.2023.154759

[132] Wang F, Jia Y, Li M, Wang L, Shao J, Guo Q, et al. Blockade of glycolysis-dependent contraction by Oroxylin A via inhibition of lactate dehydrogenase-A in hepatic stellate cells. Cell Commun Signal. 2019;17(1):11. 10.1186/s12964-019-0324-8. 30744642 10.1186/s12964-019-0324-8 PMC6371416

[133] Irshad I, Alqahtani SA, Ikejima K, Yu ML, Romero-Gomez M, Eslam M. Energy metabolism: An emerging therapeutic frontier in liver fibrosis. Ann Hepatol. 2025;30(1):101896. 10.1016/j.aohep.2025.101896. 40057035 10.1016/j.aohep.2025.101896

[134] Yan M, Cui Y, Xiang Q. Metabolism of hepatic stellate cells in chronic liver diseases: emerging molecular and therapeutic interventions. Theranostics. 2025;15(5):1715–40. 10.7150/thno.106597. 39897543 10.7150/thno.106597 PMC11780521

[135] Kisseleva T, Ganguly S, Murad R, Wang A, Brenner DA. Regulation of hepatic stellate cell phenotypes in Metabolic Dysfunction-Associated Steatohepatitis. Gastroenterology. 2025;169(5):797–812. 10.1053/j.gastro.2025.03.010. 40120772 10.1053/j.gastro.2025.03.010 PMC12416914

[136] Guillot A, Tacke F. Liver macrophages revisited: The expanding universe of versatile responses in a spatiotemporal context. Hepatol Commun. 2024;8(7). 10.1097/hc9.0000000000000491 10.1097/HC9.0000000000000491 PMC11227356 38967563

[137] Vassiliou E, Farias-Pereira R. Impact of lipid metabolism on macrophage polarization: implications for inflammation and tumor immunity. Int J Mol Sci. 2023. 10.3390/ijms241512032. 37569407 10.3390/ijms241512032 PMC10418847

[138] Ma C, Wang S, Dong B, Tian Y. Metabolic reprogramming of immune cells in MASH. Hepatology. 2025. 10.1097/hep.0000000000001371.40324062 10.1097/HEP.0000000000001371

[139] Steinberg GR, Valvano CM, De Nardo W, Watt MJ. Integrative metabolism in MASLD and MASH: Pathophysiology and emerging mechanisms. J Hepatol. 2025;83(2):584–95. 10.1016/j.jhep.2025.02.033. 40032040 10.1016/j.jhep.2025.02.033

[140] Yin X, Peng J, Gu L, Liu Y, Li X, Wu J, et al. Targeting glutamine metabolism in hepatic stellate cells alleviates liver fibrosis. Cell Death Dis. 2022;13(11):955. 10.1038/s41419-022-05409-0. 36376267 10.1038/s41419-022-05409-0 PMC9663710

[141] Wang F, Li Z, Chen L, Yang T, Liang B, Zhang Z, et al. Inhibition of ASCT2 induces hepatic stellate cell senescence with modified proinflammatory secretome through an IL-1α/NF-κB feedback pathway to inhibit liver fibrosis. Acta Pharm Sin B. 2022;12(9):3618–38. 10.1016/j.apsb.2022.03.014. 36176909 10.1016/j.apsb.2022.03.014 PMC9513497

[142] Zhang X, Zeng Y, Ying H, Hong Y, Xu J, Lin R, et al. AdipoRon mitigates liver fibrosis by suppressing serine/glycine biosynthesis through ATF4-dependent glutaminolysis. Ecotoxicol Environ Saf. 2025;289:117511. 10.1016/j.ecoenv.2024.117511. 39662457 10.1016/j.ecoenv.2024.117511

[143] Huang R, Cui H, Alshami YAMA, Fu C, Jiang W, Cai M, et al. LOX-1 rewires glutamine ammonia metabolism to drive liver fibrosis. Mol Metab. 2025;96:102132. 10.1016/j.molmet.2025.102132. 40180177 10.1016/j.molmet.2025.102132 PMC12004974

[144] Huang R, Fu P, Ma L. Kidney fibrosis: from mechanisms to therapeutic medicines. Signal Transduct Target Ther. 2023;8(1):129. 10.1038/s41392-023-01379-7. 36932062 10.1038/s41392-023-01379-7 PMC10023808

[145] Li L, Fu H, Liu Y. The fibrogenic niche in kidney fibrosis: components and mechanisms. Nat Rev Nephrol. 2022;18(9):545–57. 10.1038/s41581-022-00590-z. 35788561 10.1038/s41581-022-00590-z

[146] Kuppe C, Ibrahim MM, Kranz J, Zhang X, Ziegler S, Perales-Patón J, et al. Decoding myofibroblast origins in human kidney fibrosis. Nature. 2021;589(7841):281–6. 10.1038/s41586-020-2941-1. 33176333 10.1038/s41586-020-2941-1 PMC7611626

[147] Wei X, Hou Y, Long M, Jiang L, Du Y. Advances in energy metabolism in renal fibrosis. Life Sci. 2023;312:121033. 10.1016/j.lfs.2022.121033. 36270427 10.1016/j.lfs.2022.121033

[148] Miguel V, Shaw IW, Kramann R. Metabolism at the crossroads of inflammation and fibrosis in chronic kidney disease. Nat Rev Nephrol. 2025;21(1):39–56. 10.1038/s41581-024-00889-z. 39289568 10.1038/s41581-024-00889-z

[149] Wang Y, Li H, Jiang S, Fu D, Lu X, Lu M, et al. The glycolytic enzyme PFKFB3 drives kidney fibrosis through promoting histone lactylation-mediated NF-κB family activation. Kidney Int. 2024;106(2):226–40. 10.1016/j.kint.2024.04.016. 38789037 10.1016/j.kint.2024.04.016

[150] Yang Q, Huo E, Cai Y, Zhang Z, Dong C, Asara JM, et al. Myeloid PFKFB3-mediated glycolysis promotes kidney fibrosis. Front Immunol. 2023;14:1259434. 10.3389/fimmu.2023.1259434. 38035106 10.3389/fimmu.2023.1259434 PMC10687406

[151] Song C, Wang S, Fu Z, Chi K, Geng X, Liu C, et al. IGFBP5 promotes diabetic kidney disease progression by enhancing PFKFB3-mediated endothelial glycolysis. Cell Death Dis. 2022;13(4):340. 10.1038/s41419-022-04803-y. 35418167 10.1038/s41419-022-04803-y PMC9007962

[152] Huang Y, Cong A, Li J, Zhou Z, Zhou H, Su C, et al. Glycolysis in peritubular endothelial cells and microvascular rarefaction in CKD. J Am Soc Nephrol. 2024. 10.1681/asn.0000000000000488. 39226371 10.1681/ASN.0000000000000488 PMC11706556

[153] Yang S, Wu H, Li Y, Li L, Xiang J, Kang L, et al. Inhibition of PFKP in renal tubular epithelial cell restrains TGF-β induced glycolysis and renal fibrosis. Cell Death Dis. 2023;14(12):816. 10.1038/s41419-023-06347-1. 38086793 10.1038/s41419-023-06347-1 PMC10716164

[154] Xu S, Cheuk YC, Jia Y, Chen T, Chen J, Luo Y, et al. Bone marrow mesenchymal stem cell-derived exosomal miR-21a-5p alleviates renal fibrosis by attenuating glycolysis by targeting PFKM. Cell Death Dis. 2022;13(10):876. 10.1038/s41419-022-05305-7. 36253358 10.1038/s41419-022-05305-7 PMC9576726

[155] Gu M, Tan M, Zhou L, Sun X, Lu Q, Wang M, et al. Protein phosphatase 2Acα modulates fatty acid oxidation and glycolysis to determine tubular cell fate and kidney injury. Kidney Int. 2022;102(2):321–36. 10.1016/j.kint.2022.03.024. 35483524 10.1016/j.kint.2022.03.024

[156] Yu H, Zhu J, Chang L, Liang C, Li X, Wang W. 3-Bromopyruvate decreased kidney fibrosis and fibroblast activation by suppressing aerobic glycolysis in unilateral ureteral obstruction mice model. Life Sci. 2021;272:119206. 10.1016/j.lfs.2021.119206. 33577854 10.1016/j.lfs.2021.119206

[157] Ding H, Jiang L, Xu J, Bai F, Zhou Y, Yuan Q, et al. Inhibiting aerobic glycolysis suppresses renal interstitial fibroblast activation and renal fibrosis. Am J Physiol Renal Physiol. 2017;313(3):F561-f575. 10.1152/ajprenal.00036.2017. 28228400 10.1152/ajprenal.00036.2017

[158] Cao H, Luo J, Zhang Y, Mao X, Wen P, Ding H, et al. Tuberous sclerosis 1 (Tsc1) mediated mTORC1 activation promotes glycolysis in tubular epithelial cells in kidney fibrosis. Kidney Int. 2020;98(3):686–98. 10.1016/j.kint.2020.03.035. 32739207 10.1016/j.kint.2020.03.035

[159] Li J, Liu H, Takagi S, Nitta K, Kitada M, Srivastava SP, et al. Renal protective effects of empagliflozin via inhibition of EMT and aberrant glycolysis in proximal tubules. JCI Insight. 2020. 10.1172/jci.insight.129034. 32134397 10.1172/jci.insight.129034 PMC7213787

[160] Li SY, Tsai MT, Kuo YM, Yang HM, Tong ZJ, Cheng HW, et al. Aldehyde dehydrogenase 2 preserves kidney function by countering acrolein-induced metabolic and mitochondrial dysfunction. JCI Insight. 2024. 10.1172/jci.insight.179871. 39226171 10.1172/jci.insight.179871 PMC11466184

[161] Xu C, Hong Q, Zhuang K, Ren X, Cui S, Dong Z, et al. Regulation of pericyte metabolic reprogramming restricts the AKI to CKD transition. Metabolism. 2023;145:155592. 10.1016/j.metabol.2023.155592. 37230215 10.1016/j.metabol.2023.155592

[162] Chen L, Li X, Deng Y, Chen J, Huang M, Zhu F, et al. The PI3K-Akt-mTOR pathway mediates renal pericyte-myofibroblast transition by enhancing glycolysis through HKII. J Transl Med. 2023;21(1):323. 10.1186/s12967-023-04167-7. 37179292 10.1186/s12967-023-04167-7 PMC10182641

[163] Hu JQ, Zheng DC, Huang L, Yang X, Ning CQ, Zhou J, et al. Suppression of ZEB1 by Ethyl caffeate attenuates renal fibrosis via switching glycolytic reprogramming. Pharmacol Res. 2024. 10.1016/j.phrs.2024.107407. 39270946 10.1016/j.phrs.2024.107407

[164] Smith ER, Wigg B, Holt S, Hewitson TD. TGF-β1 modifies histone acetylation and acetyl-coenzyme A metabolism in renal myofibroblasts. Am J Physiol Renal Physiol. 2019. 10.1152/ajprenal.00513.2018. 30623724 10.1152/ajprenal.00513.2018

[165] Zhu W, Guo S, Sun J, Zhao Y, Liu C. Lactate and lactylation in cardiovascular diseases: current progress and future perspectives. Metabolism. 2024;158:155957. 10.1016/j.metabol.2024.155957. 38908508 10.1016/j.metabol.2024.155957

[166] Zhang X, Chen J, Lin R, Huang Y, Wang Z, Xu S, et al. Lactate drives epithelial-mesenchymal transition in diabetic kidney disease via the H3K14la/KLF5 pathway. Redox Biol. 2024;75:103246. 10.1016/j.redox.2024.103246. 38925041 10.1016/j.redox.2024.103246 PMC11255112

[167] Smith ER, Hewitson TD. TGF-β1 is a regulator of the pyruvate dehydrogenase complex in fibroblasts. Sci Rep. 2020;10(1):17914. 10.1038/s41598-020-74919-8. 33087819 10.1038/s41598-020-74919-8 PMC7578649

[168] Zhang Y, Wen P, Luo J, Ding H, Cao H, He W, et al. Sirtuin 3 regulates mitochondrial protein acetylation and metabolism in tubular epithelial cells during renal fibrosis. Cell Death Dis. 2021;12(9):847. 10.1038/s41419-021-04134-4. 34518519 10.1038/s41419-021-04134-4 PMC8437958

[169] Chen H, You R, Guo J, Zhou W, Chew G, Devapragash N, et al. WWP2 regulates renal fibrosis and the metabolic reprogramming of profibrotic myofibroblasts. J Am Soc Nephrol. 2024;35(6):696–718. 10.1681/asn.0000000000000328. 38502123 10.1681/ASN.0000000000000328 PMC11164121

[170] Li H, Dixon EE, Wu H, Humphreys BD. Comprehensive single-cell transcriptional profiling defines shared and unique epithelial injury responses during kidney fibrosis. Cell Metab. 2022;34(12):1977-1998.e1979. 10.1016/j.cmet.2022.09.026. 36265491 10.1016/j.cmet.2022.09.026 PMC9742301

[171] Mitrofanova A, Merscher S, Fornoni A. Kidney lipid dysmetabolism and lipid droplet accumulation in chronic kidney disease. Nat Rev Nephrol. 2023;19(10):629–45. 10.1038/s41581-023-00741-w. 37500941 10.1038/s41581-023-00741-w PMC12926870

[172] Lee LE, Doke T, Mukhi D, Susztak K. The key role of altered tubule cell lipid metabolism in kidney disease development. Kidney Int. 2024;106(1):24–34. 10.1016/j.kint.2024.02.025. 38614389 10.1016/j.kint.2024.02.025 PMC11193624

[173] Garcia NH, Gaivin RJ, Khan S, Li V, Rbaibi Y, Weisz OA, et al. Fatty acids and albumin are transported by distinct mechanisms in the proximal tubule. Am J Physiol Renal Physiol. 2025;329(4):F444-f451. 10.1152/ajprenal.00168.2025. 40875392 10.1152/ajprenal.00168.2025 PMC12486159

[174] Kang HM, Ahn SH, Choi P, Ko YA, Han SH, Chinga F, et al. Defective fatty acid oxidation in renal tubular epithelial cells has a key role in kidney fibrosis development. Nat Med. 2015;21(1):37–46. 10.1038/nm.3762. 25419705 10.1038/nm.3762 PMC4444078

[175] Pandi A, Sen N, Kalappan VM. Fatty acid transport protein 2: A novel therapeutic target in lipid metabolism and disease - A review. Int J Biol Macromol. 2025;322(Pt 4):146856. 10.1016/j.ijbiomac.2025.146856. 40825426 10.1016/j.ijbiomac.2025.146856

[176] Wang J, Wang L, Feng X, Xu Y, Zhou L, Wang C, et al. Astragaloside IV attenuates fatty acid-induced renal tubular injury in diabetic kidney disease by inhibiting fatty acid transport protein-2. Phytomedicine. 2024;134:155991. 10.1016/j.phymed.2024.155991. 39217653 10.1016/j.phymed.2024.155991

[177] Wilson PC, Muto Y, Wu H, Karihaloo A, Waikar SS, Humphreys BD. Multimodal single cell sequencing implicates chromatin accessibility and genetic background in diabetic kidney disease progression. Nat Commun. 2022;13(1):5253. 10.1038/s41467-022-32972-z. 36068241 10.1038/s41467-022-32972-z PMC9448792

[178] Mohandes S, Doke T, Hu H, Mukhi D, Dhillon P, Susztak K. Molecular pathways that drive diabetic kidney disease. J Clin Invest. 2023. 10.1172/jci165654. 36787250 10.1172/JCI165654 PMC9927939

[179] Li X, Chen J, Li J, Zhang Y, Xia J, Du H, et al. ATGL regulates renal fibrosis by reprogramming lipid metabolism during the transition from AKI to CKD. Mol Ther. 2025;33(2):805–22. 10.1016/j.ymthe.2024.12.053. 39748508 10.1016/j.ymthe.2024.12.053 PMC11853023

[180] Zhou S, Ling X, Liang Y, Feng Q, Xie C, Li J, et al. Cannabinoid receptor 2 plays a key role in renal fibrosis through inhibiting lipid metabolism in renal tubular cells. Metabolism. 2024;159:155978. 10.1016/j.metabol.2024.155978. 39097161 10.1016/j.metabol.2024.155978

[181] Sun P, Chen Q, Chen X, Zhou J, Long T, Ma Y, et al. Renal tubular S100A7a impairs fatty acid oxidation and exacerbates renal fibrosis via both intracellular and extracellular pathway. Biochim Biophys Acta Mol Basis Dis. 2025;1871(3):167656. 10.1016/j.bbadis.2025.167656. 39778778 10.1016/j.bbadis.2025.167656

[182] Sun J, Yin C, Li Z, Gao X, Li S, Gao H, et al. Natural compounds regulating fatty acid oxidation in the treatment of diabetic kidney disease. Front Nutr. 2025;12:1669557. 10.3389/fnut.2025.1669557. 41170356 10.3389/fnut.2025.1669557 PMC12568393

[183] Jia J, Tan R, Xu L, Wang H, Li J, Su H, et al. Hederagenin improves renal fibrosis in diabetic nephropathy by regulating Smad3/NOX4/SLC7A11 signaling-mediated tubular cell ferroptosis. Int Immunopharmacol. 2024;135:112303. 10.1016/j.intimp.2024.112303. 38776855 10.1016/j.intimp.2024.112303

[184] Liu YH. Kidney fibrosis: fundamental questions, challenges, and perspectives. Integr Med Nephrol Androl. 2024;11(4):4. 10.1097/imna-d-24-00027.

[185] Knol MGE, Wulfmeyer VC, Müller RU, Rinschen MM. Amino acid metabolism in kidney health and disease. Nat Rev Nephrol. 2024;20(12):771–88. 10.1038/s41581-024-00872-8. 39198707 10.1038/s41581-024-00872-8

[186] Abraham DJ, Black CM, Denton CP, Distler JHW, Domsic R, Feghali-Bostwick C, et al. An international perspective on the future of systemic sclerosis research. Nat Rev Rheumatol. 2025;21(3):174–87. 10.1038/s41584-024-01217-2. 39953141 10.1038/s41584-024-01217-2

[187] Li DJ, Berry CE, Wan DC, Longaker MT. Clinical, mechanistic, and therapeutic landscape of cutaneous fibrosis. Sci Transl Med. 2024;16(766):eadn7871. 10.1126/scitranslmed.adn7871. 39321265 10.1126/scitranslmed.adn7871 PMC12093819

[188] Guo L, Xiang W, Pan Z, Gu H, Jiang X. Post-translational modifications of collagen and its related diseases in metabolic pathways. Acta Pharm Sin B. 2025;15(4):1773–95. 10.1016/j.apsb.2025.02.007. 40486838 10.1016/j.apsb.2025.02.007 PMC12138068

[189] Marella S, Plikus MV, Gudjonsson JE. Skin fibroblasts in health and disease: from extracellular matrix remodeling to immune regulation. J Invest Dermatol. 2026. 10.1016/j.jid.2025.12.002. 41493297 10.1016/j.jid.2025.12.002

[190] Distler JHW, Launay D, Feghali-Bostwick C, Matei AE, Trojanowska M, Gudjonsson JE. Mechanisms of fibrotic tissue remodelling: insights from systemic sclerosis. Nat Rev Rheumatol. 2026;22(4):221–38. 10.1038/s41584-025-01349-z. 41588245 10.1038/s41584-025-01349-z

[191] Ghasemishahrestani Z, Khumalo NP, Ipp H, Bayat A. Cutaneous immune memory: a double-edged sword in skin repair and fibrosis. J Invest Dermatol. 2026;146(3):625–39. 10.1016/j.jid.2025.09.006. 41108291 10.1016/j.jid.2025.09.006

[192] Yu F, Chen J, Wang X, Hou S, Li H, Yao Y, et al. Metabolic reprogramming of peritoneal mesothelial cells in peritoneal dialysis-associated fibrosis: therapeutic targets and strategies. Cell Commun Signal. 2025;23(1):114. 10.1186/s12964-025-02113-2. 40016825 10.1186/s12964-025-02113-2 PMC11866825

[193] Jasiński T, Kozłowska N, Zdrojkowski Ł, Bręborowicz A, Rey B, Domino M. The role of NF-κB in peritoneal fibrosis and adhesion in humans and animals: A systematic review. Int J Mol Sci. 2026. 10.3390/ijms27052199. 41828423 10.3390/ijms27052199 PMC12984160

[194] Ferreira-Gonzalez S, Matsumoto T, Hara E, Forbes SJ. Senescence, aging and disease throughout the gastrointestinal system. Gastroenterology. 2025;169(7):1357–79. 10.1053/j.gastro.2025.06.010. 40532827 10.1053/j.gastro.2025.06.010

[195] Rieder F, Nagy LE, Maher TM, Distler JHW, Kramann R, Hinz B, et al. Fibrosis: cross-organ biology and pathways to development of innovative drugs. Nat Rev Drug Discov. 2025;24(7):543–69. 10.1038/s41573-025-01158-9. 40102636 10.1038/s41573-025-01158-9 PMC13264708

[196] Di X, Li Y, Wei J, Li T, Liao B. Targeting fibrosis: from molecular mechanisms to advanced therapies. Adv Sci. 2025;12(3):e2410416. 10.1002/advs.202410416. 10.1002/advs.202410416 PMC11744640 39665319

[197] Junho CVC, Frisch J, Soppert J, Wollenhaupt J, Noels H. Cardiomyopathy in chronic kidney disease: clinical features, biomarkers and the contribution of murine models in understanding pathophysiology. Clin Kidney J. 2023;16(11):1786–803. 10.1093/ckj/sfad085. 37915935 10.1093/ckj/sfad085 PMC10616472

[198] Noels H, van der Vorst EPC, Rubin S, Emmett A, Marx N, Tomaszewski M, et al. Renal-cardiac crosstalk in the pathogenesis and progression of heart failure. Circ Res. 2025;136(11):1306–34. 10.1161/circresaha.124.325488. 40403103 10.1161/CIRCRESAHA.124.325488 PMC12105978

[199] Park AC, Schilling JD. The cardiohepatic axis in heart failure. JACC Basic Transl Sci. 2025;10(7):101312. 10.1016/j.jacbts.2025.05.007. 40738518 10.1016/j.jacbts.2025.05.007 PMC12434209

[200] Wattacheril JJ, Abdelmalek MF, Lim JK, Sanyal AJ. AGA clinical practice update on the role of noninvasive biomarkers in the evaluation and management of nonalcoholic fatty liver disease: expert review. Gastroenterology. 2023;165(4):1080–8. 10.1053/j.gastro.2023.06.013. 37542503 10.1053/j.gastro.2023.06.013

[201] Chew NWS, Mehta A, Goh R, Koh J, Chen Y, Chong B, et al. The global cardiovascular-liver-metabolic syndemic: epidemiology, trends and challenges. Nat Rev Cardiol. 2025. 10.1038/s41569-025-01220-4. 41053364 10.1038/s41569-025-01220-4

[202] Chew NWS, Mehta A, Goh RSJ, Zhang A, Chen Y, Chong B, et al. Cardiovascular-liver-metabolic health: recommendations in screening, diagnosis, and management of metabolic dysfunction-associated steatotic liver disease in cardiovascular disease via modified Delphi approach. Circulation. 2025;151(1):98–119. 10.1161/circulationaha.124.070535. 39723980 10.1161/CIRCULATIONAHA.124.070535

[203] Liu W, Wu X, Zeng W, Chandy M, Wu JC. Cardiac fibrosis: from mechanisms and models to medicines. Trends Pharmacol Sci. 2025. 10.1016/j.tips.2025.07.016. 40877078 10.1016/j.tips.2025.07.016 PMC13378603

[204] Miguel V, Alcalde-Estévez E, Sirera B, Rodríguez-Pascual F, Lamas S. Metabolism and bioenergetics in the pathophysiology of organ fibrosis. Free Radic Biol Med. 2024;222:85–105. 10.1016/j.freeradbiomed.2024.06.001. 38838921 10.1016/j.freeradbiomed.2024.06.001

[205] Tiwari P, Verma S, Washimkar KR, Nilakanth Mugale M. Immune cells crosstalk pathways, and metabolic alterations in idiopathic pulmonary fibrosis. Int Immunopharmacol. 2024;135:112269. 10.1016/j.intimp.2024.112269. 38781610 10.1016/j.intimp.2024.112269

[206] Wang Y, Wang J, Zhang J, Wang Y, Wang Y, Kang H, et al. Stiffness sensing via Piezo1 enhances macrophage efferocytosis and promotes the resolution of liver fibrosis. Sci Adv. 2024;10(23):eadj3289. 10.1126/sciadv.adj3289. 38838160 10.1126/sciadv.adj3289 PMC11152137

[207] Amrute JM, Luo X, Penna V, Yang S, Yamawaki T, Hayat S, et al. Targeting immune-fibroblast cell communication in heart failure. Nature. 2024;635(8038):423–33. 10.1038/s41586-024-08008-5. 39443792 10.1038/s41586-024-08008-5 PMC12334188

[208] Luo J, Li P, Dong M, Zhang Y, Lu S, Chen M, et al. SLC15A3 plays a crucial role in pulmonary fibrosis by regulating macrophage oxidative stress. Cell Death Differ. 2024;31(4):417–30. 10.1038/s41418-024-01266-w. 38374230 10.1038/s41418-024-01266-w PMC11043330

[209] Wang J, Du H, Xie W, Bi J, Zhang H, Liu X, et al. CAR-Macrophage therapy alleviates myocardial ischemia-reperfusion injury. Circ Res. 2024;135(12):1161–74. 10.1161/circresaha.124.325212. 39465245 10.1161/CIRCRESAHA.124.325212

[210] Zhao X, Li Y, Yu J, Teng H, Wu S, Wang Y, et al. Role of mitochondria in pathogenesis and therapy of renal fibrosis. Metabolism. 2024;155:155913. 10.1016/j.metabol.2024.155913. 38609039 10.1016/j.metabol.2024.155913

[211] Kliment CR, Gurkar AU, Cárdenes N, Ramonell R, Finkel T, Königshoff M. Fueling the fire: metabolic dysfunction and senescence as drivers of lung aging and disease. Physiol Rev. 2026. 10.1152/physrev.00024.2025. 41789983 10.1152/physrev.00024.2025 PMC13091605

[212] Zhang X, Wang Y, Guo X, Xiao Y, Wan W, Zou H, et al. Mitochondrial dysfunction in fibrotic diseases: research progress and MSC-exos therapy. Exp Mol Pathol. 2025;143:104983. 10.1016/j.yexmp.2025.104983. 40644771 10.1016/j.yexmp.2025.104983

[213] Jin X, Ma Y, Tang Y, Qiao F, Xiao T, Cui Y, et al. Research trends of mitochondrial dysfunction in hepatic fibrosis: a bibliometric analysis. Front Physiol. 2026;17:1767822. 10.3389/fphys.2026.1767822. 41835144 10.3389/fphys.2026.1767822 PMC12982054

[214] Fan X, Yang M, Lang Y, Lu S, Kong Z, Gao Y, et al. Mitochondrial metabolic reprogramming in diabetic kidney disease. Cell Death Dis. 2024;15(6):442. 10.1038/s41419-024-06833-0. 38910210 10.1038/s41419-024-06833-0 PMC11194272

[215] Wang Y, Yang J, Zhang Y, Zhou J. Focus on mitochondrial respiratory chain: Potential therapeutic target for chronic renal failure. Int J Mol Sci. 2024. 10.3390/ijms25020949. 38256023 10.3390/ijms25020949 PMC10815764

[216] Zhang K, Zhang E, Wu K, Cheng W, Wei S. Mitochondrial homeostasis: a key regulator in endometrial physiology and pathology. Drug Discov Today. 2025;30(12):104519. 10.1016/j.drudis.2025.104519. 41167388 10.1016/j.drudis.2025.104519

[217] Zhu Y, Liu W, Luo Z, Xiao F, Sun B. New insights into the roles of lactylation in cancer. Front Pharmacol. 2024;15:1412672. 10.3389/fphar.2024.1412672. 39502530 10.3389/fphar.2024.1412672 PMC11534861

[218] Lin J, Liu G, Chen L, Kwok HF, Lin Y. Targeting lactate-related cell cycle activities for cancer therapy. Semin Cancer Biol. 2022;86(Pt 3):1231–43. 10.1016/j.semcancer.2022.10.009. 36328311 10.1016/j.semcancer.2022.10.009

[219] Chen H, Li Y, Li H, Chen X, Fu H, Mao D, et al. NBS1 lactylation is required for efficient DNA repair and chemotherapy resistance. Nature. 2024;631(8021):663–9. 10.1038/s41586-024-07620-9. 38961290 10.1038/s41586-024-07620-9 PMC11254748

[220] Ni X, Lu CP, Xu GQ, Ma JJ. Transcriptional regulation and post-translational modifications in the glycolytic pathway for targeted cancer therapy. Acta Pharmacol Sin. 2024;45(8):1533–55. 10.1038/s41401-024-01264-1. 38622288 10.1038/s41401-024-01264-1 PMC11272797

[221] Chen J, Zhu Y, Wu C, Shi J. Engineering lactate-modulating nanomedicines for cancer therapy. Chem Soc Rev. 2023;52(3):973–1000. 10.1039/d2cs00479h. 36597879 10.1039/d2cs00479h

[222] Bie J, Li R, Li Y, Song C, Chen Z, Zhang T, et al. PKM2 aggregation drives metabolism reprograming during aging process. Nat Commun. 2024;15(1):5761. 10.1038/s41467-024-50242-y. 38982055 10.1038/s41467-024-50242-y PMC11233639

[223] Adem S, Rasul A, Riaz S, Sadiqa A, Ahmad M, Shahid Nazir M, et al. Pyruvate kinase modulators as a therapy target: an updated patent review 2018–2023. Expert Opin Ther Pat. 2024;34(10):953–62. 10.1080/13543776.2024.2403616. 39279560 10.1080/13543776.2024.2403616

[224] Liu Q, Li J, Li X, Zhang L, Yao S, Wang Y, et al. Advances in the understanding of the role and mechanism of action of PFKFB3‑mediated glycolysis in liver fibrosis (review). Int J Mol Med. 2024. 10.3892/ijmm.2024.5429. 39301662 10.3892/ijmm.2024.5429 PMC11448561

[225] Cannon CP, Pratley R, Dagogo-Jack S, Mancuso J, Huyck S, Masiukiewicz U, et al. Cardiovascular outcomes with Ertugliflozin in type 2 diabetes. N Engl J Med. 2020;383(15):1425–35. 10.1056/NEJMoa2004967. 32966714 10.1056/NEJMoa2004967

[226] Corbin KD, Dagogo-Jack S, Cannon CP, Cherney DZI, Cosentino F, Frederich R, et al. Cardiorenal outcomes by indices of liver steatosis and fibrosis in individuals with type 2 diabetes and atherosclerotic cardiovascular disease: Analyses from VERTIS CV, a randomized trial of the sodium-glucose cotransporter-2 inhibitor ertugliflozin. Diabetes Obes Metab. 2023;25(3):758–66. 10.1111/dom.14923. 36394384 10.1111/dom.14923

[227] Schaub JA, AlAkwaa FM, McCown PJ, Naik AS, Nair V, Eddy S, et al. SGLT2 inhibitors mitigate kidney tubular metabolic and mTORC1 perturbations in youth-onset type 2 diabetes. J Clin Invest. 2023. 10.1172/jci164486. 36637914 10.1172/JCI164486 PMC9974101

[228] Abbad L, Esteve E, Chatziantoniou C. Advances and challenges in kidney fibrosis therapeutics. Nat Rev Nephrol. 2025;21(5):314–29. 10.1038/s41581-025-00934-5. 39934355 10.1038/s41581-025-00934-5

[229] Lukey PT, Harrison SA, Yang S, Man Y, Holman BF, Rashidnasab A, et al. A randomised, placebo-controlled study of omipalisib (PI3K/mTOR) in idiopathic pulmonary fibrosis. Eur Respir J. 2019. 10.1183/13993003.01992-2018. 30765508 10.1183/13993003.01992-2018

[230] Kalyesubula M, Von Bank H, Davidson JW, Burhans MS, Becker MM, Aljohani A, et al. Stearoyl-CoA desaturase 1 deficiency drives saturated lipid accumulation and increases liver and plasma acylcarnitines. J Lipid Res. 2025;66(6):100824. 10.1016/j.jlr.2025.100824. 40350036 10.1016/j.jlr.2025.100824 PMC12173144

[231] Ratziu V, de Guevara L, Safadi R, Poordad F, Fuster F, Flores-Figueroa J, et al. Aramchol in patients with nonalcoholic steatohepatitis: a randomized, double-blind, placebo-controlled phase 2b trial. Nat Med. 2021;27(10):1825–35. 10.1038/s41591-021-01495-3. 34621052 10.1038/s41591-021-01495-3 PMC12165723

[232] Loomba R, Noureddin M, Kowdley KV, Kohli A, Sheikh A, Neff G, et al. Combination therapies including Cilofexor and Firsocostat for bridging fibrosis and cirrhosis attributable to NASH. Hepatology. 2021;73(2):625–43. 10.1002/hep.31622. 33169409 10.1002/hep.31622

[233] Loomba R, Mohseni R, Lucas KJ, Gutierrez JA, Perry RG, Trotter JF, et al. TVB-2640 (FASN inhibitor) for the treatment of nonalcoholic steatohepatitis: FASCINATE-1, a randomized, placebo-controlled phase 2a trial. Gastroenterology. 2021;161(5):1475–86. 10.1053/j.gastro.2021.07.025. 34310978 10.1053/j.gastro.2021.07.025

[234] Davis TM, Ting R, Best JD, Donoghoe MW, Drury PL, Sullivan DR, et al. Effects of fenofibrate on renal function in patients with type 2 diabetes mellitus: the Fenofibrate Intervention and Event Lowering in Diabetes (FIELD) Study. Diabetologia. 2011;54(2):280–90. 10.1007/s00125-010-1951-1. 21052978 10.1007/s00125-010-1951-1

[235] Shan B, Guo C, Zhou H, Chen J. Tanshinone IIA alleviates pulmonary fibrosis by modulating glutamine metabolic reprogramming based on [U-(13)C(5)]-glutamine metabolic flux analysis. J Adv Res. 2025;70:531–44. 10.1016/j.jare.2024.04.029. 38697470 10.1016/j.jare.2024.04.029 PMC11976427

[236] Yoon I, Kim S, Cho M, You KA, Son J, Lee C, et al. Control of fibrosis with enhanced safety via asymmetric inhibition of prolyl-tRNA synthetase 1. EMBO Mol Med. 2023;15(7):e16940. 10.15252/emmm.202216940. 37212275 10.15252/emmm.202216940 PMC10331583

[237] Jain A, Sharma BC, Mahajan B, Srivastava S, Kumar A, Sachdeva S, et al. L-ornithine L-aspartate in acute treatment of severe hepatic encephalopathy: a double-blind randomized controlled trial. Hepatology. 2022;75(5):1194–203. 10.1002/hep.32255. 34822189 10.1002/hep.32255

[238] Guo M, Liu D, Jiang Y, Chen W, Zhao L, Bao D, et al. Serum metabolomic profiling reveals potential biomarkers in systemic sclerosis. Metabolism. 2023;144:155587. 10.1016/j.metabol.2023.155587. 37156409 10.1016/j.metabol.2023.155587

[239] Pouleur AC, Menghoum N, Cumps J, Marino A, Badii M, Lejeune S, et al. Plasma myo-inositol elevation in heart failure: clinical implications and prognostic significance. Results from the BElgian and CAnadian MEtabolomics in HFpEF (BECAME-HF) research project. EBioMedicine. 2024;107:105264. 10.1016/j.ebiom.2024.105264. 39121579 10.1016/j.ebiom.2024.105264 PMC11363489

[240] Liu X, Zhao Y, Xie Y, Shi Y, Yang J, Du Y, et al. Characterization of lipid epi-metabolites/reactions as diagnostic biomarkers for idiopathic pulmonary fibrosis by Lipidepifind. Anal Chem. 2025;97(44):24285–94. 10.1021/acs.analchem.5c00277. 41105844 10.1021/acs.analchem.5c00277

[241] Cai W, Zhang H, Li Z, Cai M, Chen P, Guo N, et al. Potential biomarkers of idiopathic pulmonary fibrosis: metabonomics driven lipid profiling. J Transl Med. 2025;23(1):1010. 10.1186/s12967-025-06975-5. 40993738 10.1186/s12967-025-06975-5 PMC12462253

[242] Chalifoux O, Dagostino C, Li M, Trezza S, Grayson C, De Sa Tavares Russo M, et al. Accumulation of succinate in the blood is a potential early indicator of metabolic dysfunction-associated steatotic liver disease (MASLD). Free Radic Biol Med. 2025;241:220–35. 10.1016/j.freeradbiomed.2025.09.029. 40983198 10.1016/j.freeradbiomed.2025.09.029

[243] Huang Y, Li J, Song S, Du B, Cao Y, Wang Y, et al. Blood metabolic panels for identifying significant fibrosis and inflammation in patients with MASLD. Cell Rep Med. 2026;7(1):102522. 10.1016/j.xcrm.2025.102522. 41421352 10.1016/j.xcrm.2025.102522 PMC12866143

[244] Yang CR, Lin WJ, Shen PC, Liao PY, Dai YC, Hung YC, et al. Phenotypic and metabolomic characteristics of mouse models of metabolic associated steatohepatitis. Biomark Res. 2024;12(1):6. 10.1186/s40364-023-00555-9. 38195587 10.1186/s40364-023-00555-9 PMC10777576

[245] Vlahou A, Vanholder R. Urine as a source of biomarkers and biological knowledge in chronic kidney disease. Nat Rev Nephrol. 2026;22(1):69–84. 10.1038/s41581-025-01008-2. 41057598 10.1038/s41581-025-01008-2

[246] Heneberg P, Heneberg Šimčíková D. Untangling amino acid metabolism in renal diseases: mechanisms, dysregulation, and critical gaps. Clin Kidney J. 2026;19(2):sfaf380. 10.1093/ckj/sfaf380. 41641314 10.1093/ckj/sfaf380 PMC12865306

[247] Seubnooch P, Montani M, Dufour JF, Masoodi M. Spatial lipidomics reveals zone-specific hepatic lipid alteration and remodeling in metabolic dysfunction-associated steatohepatitis. J Lipid Res. 2024;65(9):100599. 10.1016/j.jlr.2024.100599. 39032559 10.1016/j.jlr.2024.100599 PMC11388789

[248] Pan S, Yin L, Liu J, Tong J, Wang Z, Zhao J, et al. Metabolomics-driven approaches for identifying therapeutic targets in drug discovery. MedComm (2020). 2024;5(11):e792. 10.1002/mco2.792. 39534557 10.1002/mco2.792 PMC11555024

[249] Wang X, Liu Y, Jiang C, Huang Z, Yan H, Wong SH, et al. TidyMass2: advancing LC-MS untargeted metabolomics through metabolite origin inference and metabolic feature-based functional module analysis. Nat Commun. 2026;17(1):1755. 10.1038/s41467-026-68464-7. 41545383 10.1038/s41467-026-68464-7 PMC12913914

[250] Mohanta SK, Heron C, Klaus-Bergmann A, Horstmann H, Brakenhielm E, Giannarelli C, et al. Metabolic and immune crosstalk in cardiovascular disease. Circ Res. 2025;136(11):1433–53. 10.1161/circresaha.125.325496. 40403115 10.1161/CIRCRESAHA.125.325496 PMC12286643

[251] Pająk B, Zieliński R, Priebe W. The impact of glycolysis and its inhibitors on the immune response to inflammation and autoimmunity. Molecules. 2024. 10.3390/molecules29061298. 38542934 10.3390/molecules29061298 PMC10975218

[252] Bhatt AN, Shenoy S, Munjal S, Chinnadurai V, Agarwal A, Vinoth Kumar A, et al. 2-deoxy-D-glucose as an adjunct to standard of care in the medical management of COVID-19: a proof-of-concept and dose-ranging randomised phase II clinical trial. BMC Infect Dis. 2022;22(1):669. 10.1186/s12879-022-07642-6. 35927676 10.1186/s12879-022-07642-6 PMC9351257

